# The Entropy Gain of Linear Systems and Some of Its Implications

**DOI:** 10.3390/e23080947

**Published:** 2021-07-24

**Authors:** Milan S. Derpich, Matias Müller, Jan Østergaard

**Affiliations:** 1 Department of Electronic Engineering, Universidad Técnica Federico Santa María, Av. España 1680, Valparaíso 2390123, Chile; 2 Department of Electronic Systems, Aalborg University, 9220 Aalborg, Denmark

**Keywords:** differential entropy rate, non-minimum phase linear time-invariant systems, entropy loss in linear filters, networked control, rate-distortion for non-stationary sources feedback capacity

## Abstract

We study the increase in per-sample differential entropy rate of random sequences and processes after being passed through a non minimum-phase (NMP) discrete-time, linear time-invariant (LTI) filter *G*. For LTI discrete-time filters and random processes, it has long been established by Theorem 14 in Shannon’s seminal paper that this entropy gain, G(G), equals the integral of $\log|G(e^{j\omega })|$. In this note, we first show that Shannon’s Theorem 14 does not hold in general. Then, we prove that, when comparing the input differential entropy to that of the entire (longer) output of *G*, the entropy gain equals G(G). We show that the entropy gain between equal-length input and output sequences is upper bounded by G(G) and arises if and only if there exists an output additive disturbance with finite differential entropy (no matter how small) or a random initial state. Unlike what happens with linear maps, the entropy gain in this case depends on the distribution of all the signals involved. We illustrate some of the consequences of these results by presenting their implications in three different problems. Specifically: conditions for equality in an information inequality of importance in networked control problems; extending to a much broader class of sources the existing results on the rate-distortion function for non-stationary Gaussian sources, and an observation on the capacity of auto-regressive Gaussian channels with feedback.

## 1. Introduction

**Figure 1:**
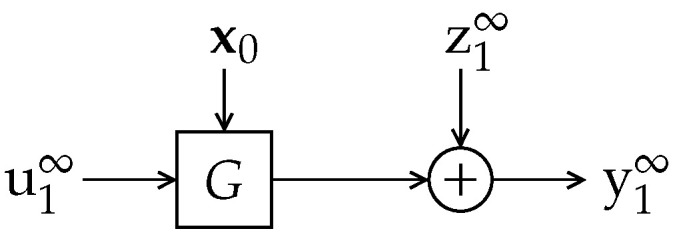
A causal, stable, linear and time-invariant system *G* with input and output processes, initial state, and output disturbance.

We study the difference between the differential entropy rate of a random process $u_{1}^{\infty }=\left\{u_{1},u_{2},\ldots \right\}$ entering a discrete-time *linear time-invariant* (LTI) system *G* and the differential entropy rate of its (possibly noisy) output $y_{1}^{\infty }$, as depicted in Figure 1.

Recall that the differential entropy rate of a random process $x_{1}^{\infty }$ is given by $\bar{h}\left(x_{1}^{\infty }\right)≜\lim_{n\to \infty }n^{-1}h\left(x_{1},x_{2},\ldots ,x_{n}\right)$, provided the limit exists, where $h\left(x_{1},\ldots ,x_{n}\right)=E\left[-\log f(x_{1},\ldots ,x_{n})\right]$ is the differential entropy of the ensemble $x_{1},\ldots ,x_{n}$ with *probability density function* (PDF) *f* [1]. The system *G* is supposed to satisfy the following:

**Assumption** **1.** *The LTI system G in Figure 1 is causal and stable and such that*

*1*. *G has a rational p-th order transfer function G(z) with m zeros ${\left\{\rho_{i}\right\}}_{i=1}^{m}$ outside the unit circle, i.e.,* non-minimum-phase *(NMP) zeros, where m∈{0,1,…,p}, indexed in non-increasing magnitude order, i.e., $|\rho_{1}\left|\geq \right|\rho_{2}\left|\geq \cdots \geq \right|\rho_{m}|>1$.*
*2*. *The unit-impulse response of G, say, $g_{0},g_{1},\ldots$ satisfies $|g_{0}|=1$.*

In this general setup, *G* may have a random initial state vector $x_{0}\in R^{p}$, p∈N, and a real-valued random output disturbance $z_{1}^{\infty }$. Our main purpose is to characterize the limit

(1) $$\begin{array}{c} G\left(G,x_{0},u_{1}^{\infty },z_{1}^{\infty }\right)≜\lim_{n\to \infty }\frac{1}{n}\left(h\left(y_{1}^{n}\right)-h\left(u_{1}^{n}\right)\right), \end{array}$$

evaluating the possible effect produced by $x_{0}$ and $z_{1}^{\infty }$. This difference can be interpreted as the *entropy gain* (entropy amplification or entropy boost) introduced by the filter *G* and (as apparent from the other variables in the argument of G) the statistics of $x_{0},u_{1}^{\infty },z_{1}^{\infty }$. We shall refer to the special case in which $x_{0}$ and $z_{1}^{\infty }$ are both zero (or deterministic) as the noise-less case, and write $G(G,0,u_{1}^{\infty },0)$ accordingly.

The earliest reference related to this problem corresponds to a noise-less continuous-time counterpart considered by Shannon. In his seminal 1948 paper [2], Shannon gave a formula for the change in differential entropy per degree of freedom that a continuous-time random process $u_{c}$, band-limited to a frequency range [0,B) (in Hz), experiences after passing through an LTI continuous-time filter $G_{c}$ (without considering a random initial state or an output disturbance). Such entropy per degree of freedom is defined in terms of uniformly taken samples as

(2) $$\begin{array}{cc} \bar{h}\left(u_{c}\right) & ≜\lim_{n\to \infty }\frac{1}{n}h\left(u_{c}\left(T\right),u_{c}\left(2T\right),\ldots ,u_{c}\left(nT\right)\right), \end{array}$$

with T≜1/(2B). In this formula, if the LTI filter has frequency response $G_{c}\left(\xi \right)$ (with ξ in Hz), then the resulting differential entropy rate of the output process $y_{c}$ is given by the following theorem:

**Theorem** **1** (Reference [2], Theorem 14)**.** *If an ensemble having an entropy $\bar{h}\left(u_{c}\right)$ per degree of freedom in band B is passed through a filter with characteristic $G_{c}\left(\xi \right)$ the output ensemble has an entropy*

(3) $$\begin{array}{c} \bar{h}\left(y_{c}\right)=\bar{h}\left(u_{c}\right)+\frac{2}{B}\int_{0}^{B}\log\left|G_{c}\left(\xi \right)\right|d\xi . \end{array}$$

Shannon arrived at (3) by arguing that an LTI filter can be seen as a linear operator that selectively scales its input signal along infinitely many frequencies, each of them representing an orthogonal component of the source. He then obtained the result by writing down the determinant of the Jacobian of this operator as the product of the squared frequency response magnitude of the filter over *n* frequency bands, applying logarithm, dividing by *n*, and then taking the limit as *n* tends to infinity.

**Remark** **1.** *There is a factor of two in excess in the integral on the* right-hand side *(RHS) of (3). To see this, consider a filter with a constant gain a over [0,B) (i.e., a simple multiplicative factor). In such case, the entropy rate of $y_{c}$ should exceed that of $u_{c}$ by $\log\left|a\right|$ [1]. However, (3) yields an entropy gain equal to $2\log\left|a\right|$. This error arises because the determinant of the Jacobian of the transformation is actually the product of $\left|G_{c}\right|$ over the n frequency bands considered in Shannon’s argument. Such excess factor of two is also present in the entropy losses appearing in Reference [2], Table 1.*

Theorem 14 in Reference [2] has found application in works ranging from traditional themes, such as linear prediction [3] and source coding [4], to molecular communication systems [5,6].

The available literature treating the phenomenon itself of the entropy gain (loss, boost, or amplification) induced by LTI systems seems to be rather scarce. This is not surprising given that (3) was published in Reference [2], Theorem 14, the work which gave birth to Information Theory.

The following publication concerned with this problem is Reference [7], following a time-domain analysis for the corresponding discrete-time problem. In this approach, one can obtain $y_{1}^{n}≜\left\{y\left(1\right),y\left(2\right),\ldots ,y\left(n\right)\right\}$ as a function of $u_{1}^{n}$, for every n∈N, and evaluate the difference between the limits $\bar{h}\left(y_{1}^{\infty }\right)$ and $\bar{h}\left(u_{1}^{\infty }\right)$, obtained by letting n→∞. More precisely, for an LTI discrete-time filter *G* with impulse response $g_{0}^{\infty }=\left\{g_{0},g_{1},\ldots \right\}$, we can write

(4) $$\begin{array}{c} y_{n}^{1}=\underset{G_{n}}{\underbrace{\left(\begin{array}{cccc} g_{0} & 0 & \cdots & 0\\ g_{1} & g_{0} & \cdots & 0\\ \vdots & \; & \ddots & \vdots \\ g_{n-1} & g_{n-2} & \cdots & g_{0} \end{array}\right)}}u_{n}^{1}, \end{array}$$

where we adopt the notation $y_{n}^{1}$ for column vectors to avoid the abuse of notation incurred by treating the sequence $y_{1}^{n}$ as a vector, and because, by writing $y_{n}^{1}$, it is easier to remember that its samples are ordered from top to bottom. $y_{n}^{1}≜{\left[y\left(1\right)\;y\left(2\right)\;\cdots \;y\left(n\right)\right]}^{T}$ and the random vector $u_{n}^{1}$ is defined likewise. From this, it is clear (see, e.g., the corollary after Theorem 8.6.4 in Reference [1]) that

(5) $$\begin{array}{c} h\left(y_{n}^{1}\right)=h\left(u_{n}^{1}\right)+\log\left|\det\left(G_{n}\right)\right|, \end{array}$$

where $\det(G_{n})$ (or simply $\det G_{n}$) stands for the determinant of $G_{n}$. This result is utilized in Reference [7] to show that no entropy gain is produced by a stable minimum phase LTI system *G* if and only if the first sample in its impulse response has unit magnitude.

In Reference [8], p. 568, the entropy gain of a discrete-time LTI system *G* (the noise-less version of the setup depicted in Figure 1) is found to be

(6) $$\begin{array}{c} \bar{h}\left(y_{1}^{\infty }\right)=\bar{h}\left(u_{1}^{\infty }\right)+\frac{1}{2\pi }\int_{-\pi }^{\pi }\log\left|G(e^{j\omega })\right|d\omega , \end{array}$$

where $y_{1}^{\infty }$ is the filter’s discrete-time output process (without the effect of random initial state or an output disturbance) and

(7) $$\begin{array}{c} \bar{h}\left(y_{1}^{\infty }\right)≜\lim_{n\to \infty }\frac{1}{n}h\left(y_{1}^{n}\right). \end{array}$$

This result was obtained starting from the fact that, for a Gaussian stationary process $u_{1}^{\infty }$ with *power spectral density* (PSD) $S_{u}\left(e^{j\omega }\right)$, $\bar{h}\left(u_{1}^{\infty }\right)=\frac{1}{2\pi }\;\;\int_{-\pi }^{\pi }S_{u}\left(e^{j\omega }\right)d\omega$. If $u_{1}^{\infty }$ enters a discrete-time LTI system with frequency response $G(e^{j\omega })$, then the PSD of its output $y_{1}^{\infty }$ is $S_{y}\left(e^{j\omega }\right)=S_{u}\left(e^{j\omega }\right){\left|G(e^{j\omega })\right|}^{2}$; thus, it is argued that (6) follows for Gaussian stationary inputs. Then, Reference [8] extends the result for non-Gaussian inputs with a proof sketch which uses a time-domain relation, like (4), to point out that the filter is a linear operator and, as such, the differential entropy of its output exceeds that of its input by a quantity that is independent of the input distribution. (It is worth noting that (6) is the discrete-time equivalent of (3) (without its wrong factor of 2), which follows directly from the correspondence between sampled band-limited continuous-time systems and discrete-time systems.)

It is in Reference [9], Section II-C, where, for the first time, it is shown that, for a stationary Gaussian input $u_{1}^{\infty }$, the full entropy gain predicted by (6) takes place if the system output $y_{1}^{\infty }$ is contaminated by an additive output disturbance of length *p* and positive definite covariance matrix, where *p* is the order of G(z).

The integral $\frac{1}{2\pi }\int_{-\pi }^{\pi }\log\left|G(e^{j\omega })\right|d\omega$ can be related to the structure of the filter *G*. It is well known (from Jensen’s formula) that if *G* has a causal and stable rational transfer function G(z) and an impulse response with its first sample $g_{0}≜\lim_{z\to \infty }G\left(z\right)$, then

(8) $$\begin{array}{c} \frac{1}{2\pi }\int_{-\pi }^{\pi }\log\left|G(e^{j\omega })\right|d\omega =\log\left|g_{0}\right|+\sum_{i:\left|\rho_{i}\right|>1}\log\left|\rho_{i}\right|, \end{array}$$

where $\left\{\rho_{i}\right\}$ are the zeros of G(z) (see, e.g., References [10,11]). This provides a straightforward formula to evaluate $\frac{1}{2\pi }\int_{-\pi }^{\pi }\log\left|G(e^{j\omega })\right|d\omega$ of a given LTI filter with rational transfer function G(z). When combined with (6), this equation also reveals that if the entropy gain $G(u_{1}^{\infty },y_{1}^{\infty })$ is negative (i.e., if it corresponds to an entropy loss), then $|g_{0}|<1$ (with the corresponding change of variables, this is the case in all the examples given by Shannon in Reference [2], Table 1). More importantly, (8) allows us to concentrate, without loss of generality, on LTI systems G(z), whose first impulse-response sample has unit magnitude, as required by Assumption 1. Under the latter condition, (8) shows that the entropy gain is greater than zero if and only if G(z) has zeros outside the unit disk $D≜\{\rho \in C:\left|\rho \right|\leq 1\}$. A system with the latter property is said to be *non-minimum phase* (NMP); conversely, a system with all its zeros inside D is said to be *minimum phase* (MP) [11].

### 1.1. Main Contributions of this Paper

The main contributions of this paper can be summarized as follows:

1. Our first main result is showing that (6) and (3) do not hold for a large class of continuous-time filters and inputs. To see this, notice that
  (9) $$\begin{array}{c} |g_{0}|=1⟹|\det\left(G_{n}\right)|=1,\;\forall n\in N, \end{array}$$
  which, in view of (5), is equivalent to $h\left(y_{n}^{1}\right)=h\left(u_{n}^{1}\right),\;\forall n\in N$. In turn, this implies that $\bar{h}\left(y_{1}^{\infty }\right)-\bar{h}\left(u_{1}^{\infty }\right)=0$, regardless of whether G(z) (i.e., the polynomial $g_{0}+g_{1}z^{-1}+\cdots$) has zeros with magnitude greater than one (choose, for example, $g_{0}=1,\;g_{1}=2$, and $g_{k}=0$ for k≥2). This reveals that (4) holds if and only G(z) is MP. But (6) and (3) are equivalent (correcting for the in excess factor of 2 discussed in Remark 1); thus, Theorem 14 in Reference [2] also does not hold for a class of continuous-time filters. However, the transfer function $G_{c}\left(s\right)$ of a band-limited continuous-time filter $G_{c}$ is defined only for imaginary values of *s* (because the bilateral Laplace transform of sin(t)/t converges only on the imaginary axis), so one cannot classify such filters as MP or NMP. Instead, we consider a class of continuous-time filters limited to the frequencies in the band [0,B), where B>0 is in [Hz], defined by having a unit-impulse response of the form
  (10) $$\begin{array}{c} g\left(t\right)≜\sum_{k=0}^{\eta }g_{k}\phi_{k}\left(t\right), \end{array}$$
  for some absolutely summable sequence of real-valued coefficients ${\left\{g_{i}\right\}}_{i=0}^{\eta }$, η=1,2,…, where the sinc functions
  (11) $$\begin{array}{cc} \phi_{k}\left(t\right) & ≜\frac{\sin(2\pi B[t-k/(2B)])}{\pi [t-k/(2B)]}. \end{array}$$
  Since every such *g* satisfies g(k/(2B))=0 for k<0, it makes sense to refer to such filters as “sample-wise causal”. For this class of band-limited filters, we show that Theorem 14 holds if and only if the *z*-transform of ${\left\{g_{i}\right\}}_{i=0}^{\eta }$ is MP:

**Theorem** **2.** *Suppose $G_{c}$ is a low-pass continuous-time filter with unit-impulse response as in (10). Let the continuous-time random input of $G_{c}$ be*

(12) $$\begin{array}{c} u_{c}\left(t\right)=\frac{1}{2B}\sum_{k=1}^{\infty }u\left(k\right)\phi_{k}\left(t\right), \end{array}$$

*for some random sequence ${\left\{u\left(k\right)\right\}}_{k=1}^{\infty }$, with $\phi_{k}$ as in (11), and denote its output as $y_{c}$. Then,*

(13) $$\begin{array}{c} \bar{h}\left(y_{c}\right)-\bar{h}\left(u_{c}\right)=\log\left|g_{0}\right|=\log\left|\frac{1}{B}\int_{0}^{B}ℜ\left\{G_{c}\left(\xi \right)\right\}d\xi \right|\overset{\left(a\right)}{\leq }\frac{1}{B}\int_{0}^{B}\log\left|G_{c}\left(\xi \right)\right|d\xi , \end{array}$$

*with equality in (a) if and only if the polynomial $g_{0}+g_{1}z^{-1}+g_{2}z^{-2}\cdots$ has no roots outside the unit circle.*

2. We show that $\frac{1}{2\pi }\;\;\int_{-\pi }^{\pi }\log\left|G(e^{j\omega })\right|d\omega$ actually corresponds to the entropy gain introduced by *G* but considering the new notion of effective differential entropy rate of $y_{1}^{\infty }$ proposed in this paper, defined next.
  **Definition** **1** (The Effective Differential Entropy)**.** *Let $y\in R^{\ell }$ be a random vector. If y can be written as a linear transformation y=Su, for some $u\in R^{n}$ (n≤ℓ) with bounded differential entropy, $S\in R^{\ell \times n}$, then the effective differential entropy of y is defined as*
  (14) $$\begin{array}{c} \overset{˘}{h}\left(y\right)≜h\left(Ay\right), \end{array}$$
  *where $S=A^{T}TC$ is an SVD for S, with $T\in R^{n\times n}$.*
  We can now state our second main result, the proof of which is in Appendix A:
  **Theorem** **3.** *Let $u_{1}^{\infty }$ be the input of an LTI system G with transfer function G(z) without zeros on the unit circle and with an absolutely summable unit impulse response ${\left\{g_{i}\right\}}_{i=0}^{\eta -1}$, with η=∞ if G has an infinite impulse response. Denote the output of G as $y_{1}^{\infty }$. Suppose $\left|h(u_{1}^{n})\right|<\infty$ for every finite n. Then,*
  (15) $$\begin{array}{c} \lim_{n\to \infty }\frac{1}{n}\left(\overset{˘}{h}\left(y_{1}^{n+\eta }\left(u_{1}^{n}\right)\right)-h\left(u_{1}^{n}\right)\right)=\frac{1}{2\pi }\;\;\int_{-\pi }^{\pi }\log\left|G(e^{j\omega })\right|d\omega , \end{array}$$
  *where $y_{1}^{n+\eta }\left(u_{1}^{n}\right)$ denotes the entire response of G to the input $u_{1}^{n}$.*
  Theorem 3 states that, when considering the full-length output of a system, the effective entropy gain is introduced by the system itself.
  Section 4 provides a geometrical description of the phenomenon behind Definition 1 and Theorem 3.
We show that $\frac{1}{2\pi }\int_{-\pi }^{\pi }\log\left|G(e^{j\omega })\right|d\omega$ is a tight upper bound to the entropy gain of *G* (as defined in (1)), when the output is contaminated by some additional additive signal, such as a random initial state (represented by $x_{0}$ in Figure 1) or an output disturbance (such as $z_{1}^{\infty }$ in Figure 1), with sufficiently many degrees of freedom (a condition formally stated in Assumption 2 below). Moreover, we show that an entropy gain equal to the latter upper bound can appear even when these disturbances or random initial state have infinitesimally small variances. To the best of our knowledge, the latter phenomenon has been discussed in the literature first (and only) in Reference [9], Section II-C, for Gaussian stationary inputs and an LTI filter. We go beyond the latter result by explicitly and fully characterizing the entropy gain of LTI systems for a large class of not necessarily Gaussian nor stationary random input. We refer to this class as *entropy-balanced* processes, formally specified in the following definition:
  **Definition** **2.** *A random process ${\left\{v\left(k\right)\right\}}_{k=1}^{\infty }$ is said to be entropy balanced if the following two conditions are satisfied:*
  *(i)*  *Its sample variances $\sigma_{v(n)}^{2}$ are finite for finite n and*
    (16) $$\begin{array}{c} \lim_{n\to \infty }\frac{1}{n}\log\left(\sigma_{v(n)}^{2}\right)=0. \end{array}$$
  *(ii)*  *For every ν∈N and for every sequence of matrices ${\left\{\Phi_{n}\right\}}_{n=\nu +1}^{\infty }$, $\Phi_{n}\in R^{(n-\nu )\times n}$ with orthonormal rows,*
    (17) $$\begin{array}{cc} \lim_{n\to \infty }\frac{1}{n} & \left(h\left(\Phi_{n}v_{n}^{1}\right)-h\left(v_{n}^{1}\right)\right)=0. \end{array}$$
  The second condition guarantees that projecting an entropy-balanced process onto any subspace having finitely fewer dimensions yields a process with the same differential entropy rate.
  The entropy gain induced by finite-length output disturbances is characterized by our next theorem.
  **Theorem** **4.** *In the system of Figure 1, let G satisfy Assumption 1 and suppose that $u_{1}^{\infty }$ is entropy balanced. Suppose the random output disturbance $z_{1}^{\infty }$ is such that z(i)=0,∀i>κ, and that $|h(z_{1}^{\kappa })|<\infty$. Let $\bar{\kappa }≜\min\left\{\kappa ,m\right\}$, where m is the number of NMP zeros of G(z). Then,*
  (18) $$\sum_{i=m-\bar{\kappa }+1}^{m}\log|\rho_{i}|\leq {\lim\sup}_{n\to \infty }\frac{1}{n}\left(h\left(y_{n}^{1}\right)-h\left(u_{n}^{1}\right)\right)\leq \sum_{i=1}^{\bar{\kappa }}\log\left|\rho_{i}\right|\overset{\left(a\right)}{\leq }\frac{1}{2\pi }\;\;\int_{-\pi }^{\pi }\log\left|G(e^{j\omega })\right|d\omega$$
  *with equality in (a) if and only if κ≥m.*
  The proof is presented in Section 6.4, and we provide geometrical insight explaining the phenomenon underlying Definition 2 and Theorem 4 in Section 5.1.
We illustrate the relevance of the results summarized above by applying them to three problems in three areas, namely:
  (a) *Networked Control:* We show that equality holds in the inequality stated in Reference [12], Lemma 3.2 (a fundamental piece for the performance limitation results further developed in Reference [13]), under very general conditions. In addition, we extend the validity of a related equality for the perfect-feedback case, given by Reference [14], Theorem 14, for Gaussian signals, to the much larger class of entropy-balanced processes.
  (b) *The rate-distortion function for non-stationary Gaussian sources:* This problem has been previously solved in References [15,16,17]. We provide a simpler proof based upon the results described above. This proof extends the result stated in References [16,17] to a broader class of non-stationary sources.
  (c) *Gaussian channel capacity with feedback:* We show that capacity results based on using a short random sequence as channel input and relying on a feedback filter which boosts the entropy rate of the end-to-end channel noise (such as the one proposed in Reference [9]), crucially depend upon the complete absence of any additional disturbance anywhere in the system. Specifically, we show that the information rate of such capacity-achieving schemes drops to zero in the presence of any such additional disturbance. As a consequence, the relevance of characterizing the robust (i.e., in the presence of disturbances) feedback capacity of Gaussian channels, which appears to be a fairly unexplored problem, becomes evident.

### 1.2. Paper Outline

The remainder of this paper begins with some necessary definitions and preliminary results in Section 2. It continues with our detailed exposition in Section 3 of why Shannon’s reasoning fails to yield the right expression for the entropy gain. We present an intuitive discussion leading to the definition of effective differential entropy in Section 4, which is ended by the proof of Theorem 3. Section 5 gives a geometric interpretation of how an arbitrarily small additive perturbation is able to boost the differential entropy rate of the process coming out of an NMP LTI filter. This exposition helps understanding and justifies the introduction of *entropy-balanced* random processes, which are also characterized there. Section 6 and Section 7 contain our results for the entropy gain produced by an output disturbance and a random initial state, respectively. Our illustrative application results are presented in Section 8, followed by our conclusions in Section 9. Except when presented right after a statement or in its own section, all proofs are given in Appendix B.

## 2. Preliminaries

### 2.1. Notation

The sets of natural, real and complex numbers are denoted N, R, and C, respectively. For a complex *x*, ℜ{x} is the real part of *x*. For a set S, the indicator function $1_{S}\left(x\right)$ equals 1 if x∈S and 0 otherwise. For any LTI system *G*, the transfer function G(z) corresponds to the *z*-transform of the impulse response $g_{0},\;g_{1},\ldots$, i.e., $G\left(z\right)=\sum_{i=0}^{\infty }g_{i}z^{-i}$. For a transfer function G(z), we denote by $G_{n}\in R^{n\times n}$ the lower triangular Toeplitz matrix having ${\left[g_{0}\;\cdots \;g_{n-1}\right]}^{T}$ as its first column. We write $x_{1}^{n}$ as a shorthand for the sequence $\left\{x_{1},\ldots ,x_{n}\right\}$, and, when convenient, we write $x_{1}^{n}$ in vector form as $x_{n}^{1}≜{\left[x_{1}\;x_{2}\;\cdots \;x_{n}\right]}^{T}$, where ${\left(\right)}^{T}$ denotes transposition. Random scalars (vectors) are denoted using non-italic characters, such as x (non-italic and boldface characters, such as x). The notation x⫫y means x and y are independent. If x and z are conditionally independent given y, we write x⟷y⟷z. For matrices, we use upper-case boldface symbols, such as A. We write $\lambda_{i}\left(A\right)$ to denote the *i*-th eigenvalue of A sorted in increasing magnitude. If $A\in C^{m\times n}$, $A^{H}$ is its conjugate transpose, and $\sigma_{i}\left(A\right)≜\sqrt{\lambda_{i}\left(A^{H}A\right)}$, if m≥n, and $\sigma_{i}\left(A\right)≜\sqrt{\lambda_{i}\left(A\;A^{H}\right)}$, if m<n. We define $\sigma_{\min}\left(A\right)≜\sigma_{1}\left(A\right)$ and $\sigma_{\max}\left(A\right)≜\sigma_{\min\{m,n\}}\left(A\right)$. The term $A_{i,j}$ denotes the entry in the intersection between the *i*-th row and the *j*-th column. If $A\in C^{m\times n}$, then $A^{T}$ and $A^{*}$ denote the transpose and conjugate transpose of A, respectively. We write ${\left[A\right]}_{i_{2}}^{i_{1}}$, with $1\leq i_{1}\leq i_{2}\leq m$, to refer to the matrix formed by selecting the rows $i_{1}$ to $i_{2}$ of A. Likewise, for $1\leq j_{1}\leq j_{2}\leq n$, $\overset{j_{1}\;j_{2}}{\overset{⎴}{A}}$ is the matrix built with columns $j_{1}$ to $j_{2}$ of A. The expression ${}^{m_{1}}\;{\left[A\right]}_{m_{2}}$ corresponds to the square sub-matrix along the main diagonal of A, with its top-left and bottom-right corners on $A_{m_{1},m_{1}}$ and $A_{m_{2},m_{2}}$, respectively. A diagonal matrix whose entries are the elements in a set D (wherein elements may be repeated) is denoted as diagD. If $A\in R^{n\times m_{1}}$ and $B\in R^{n\times m_{2}}$, we write $\left[A|B\right]\in R^{n\times (m_{1}+m_{2})}$ to denote the augmented matrix built by placing the columns of A followed by those of B.

### 2.2. Mutual Information and Differential Entropy

Let x, y, and z be random variables with joint PDF $f_{x,y,z}$, and marginal PDFs $f_{x}$, $f_{y}$, and $f_{z}$, respectively. The mutual information between x and y is defined as $I\left(x;y\right)≜\int f_{x,y}\left(x,y\right)\log\left(\frac{f_{x,y}\left(x,y\right)}{f_{x}\left(x\right)f_{y}\left(y\right)}\right)dxdy$. The conditional mutual information between x and y given z is defined as $I\left(x;y|z\right)≜\int f_{x,y,z}\left(x,y,z\right)\log\left(\frac{f_{x,y|z}\left(x,y|z\right)}{f_{x|z}\left(x|z\right)f_{y|z}\left(y|z\right)}\right)dxdydz$, where $f_{x,y|z}$ is the joint PDF of x and y given z, and $f_{x|z}$, $f_{y|z}$ are defined likewise. The conditional differential entropy of x given y is defined as $h\left(x|y\right)≜-\int f_{x,y}\left(x,y\right)\log\left(f_{x|y}\left(x|y\right)\right)dxdy$.

From these definitions, it is easy to verify the following properties Reference [1], Sections 2.4–2.6 and 8.4–8.6:

- Shift invariance: for every deterministic function *f*,
  (19) $$\begin{array}{c} h(x+f(y)|y)=h(x|y). \end{array}$$
- Non-negativity:
  (20) $$\begin{array}{c} I(x;y)\geq 0, \end{array}$$
  with equality if and only if x and y are independent.
- Chain Rule:
  (21) $$\begin{array}{c} I(x;y,z)=I(x;y)+I(x;z|y). \end{array}$$
- Relationship with entropy:
  (22) $$\begin{array}{c} I(x;y)=h(x)-h(x|y)=h(y)-h(y|x). \end{array}$$

### 2.3. System Model and Assumptions

Consider the discrete-time system depicted in Figure 1. In this setup, the block *G* satisfies Assumption 1.

It is worth noting that there is no loss of generality in considering $g_{0}=1$, since one can otherwise write G(z) as $G^{\prime }\left(z\right)=g_{0}\cdot \left(G\left(z\right)/g_{0}\right)$; thus, the entropy gain introduced by $G^{\prime }\left(z\right)$ would be $\log|g_{0}|$ plus the entropy gain due to $G\left(z\right)/g_{0}$ (in agreement with (6)), which has an impulse response with its first sample equal to 1.

The following assumption is made about the output disturbance $z_{1}^{\infty }$:

**Assumption** **2.** *The disturbance $z_{1}^{\infty }$ is independent of $u_{1}^{\infty }$ and belongs to a κ-dimensional linear subspace, for some finite κ∈N. This subspace is spanned by the κ orthonormal columns of a matrix $\Phi \in R^{|N|\times \kappa }$ (where |N| stands for the countably infinite size of N), such that $|h(\Phi^{T}z_{\infty }^{1})|<\infty$. Moreover, $z_{\infty }^{1}=\Phi s_{\kappa }^{1}$, where the random vector $s_{\kappa }^{1}≜\Phi^{T}z_{\infty }^{1}$ has finite differential entropy, its covariance matrix $K_{s_{\kappa }^{1}}$ satisfies $\lambda_{\max}\left(K_{s_{\kappa }^{1}}\right)<\infty$, and it is independent of $u_{\infty }^{1}$.*

## 3. Revisiting Theorem 14 in Reference Shannon et al.

In this section, after presenting the proof of Theorem 2, we develop Shannon’s approach into a more detailed and formal exposition. This allows us to explain why, for part of the continuous-time filters considered in Theorem 2, the approach chosen by Shannon to prove Theorem 14 in Reference [2] is unable to predict the correct value for the entropy gain.

### 3.1. Proof of Theorem 2

To begin with, the Fourier transform of $\phi_{k}$ is

(23) $$\begin{array}{c} \Phi_{k}\left(\xi \right)≜\int_{-\infty }^{\infty }\phi_{k}\left(t\right)e^{-j2\pi \xi t}dt=1_{\left[-B,B\right]}\left(\xi \right)e^{-j2\pi \xi k/(2B)}. \end{array}$$

It is easy to verify that the functions $\phi_{k}$ satisfy the following orthogonality property:

(24) $$\begin{array}{c} \int_{-\infty }^{\infty }\phi_{k}\left(t\right)\phi_{i}\left(t\right)dt=\left\{\begin{array}{cc} 2B & ,\;k=i\\ 0 & ,\;k\neq i \end{array}\right. \end{array}$$

and

(25) $$\begin{array}{c} \phi_{k}\left(-t\right)=\phi_{-k}\left(t\right). \end{array}$$

Notice that $u\left(k\right)=u_{c}\left(\frac{k}{2B}\right)$, k∈N.

The output of $G_{c}$ sampled at time t=ℓ/(2B), ℓ∈N, is

(26) $$\begin{array}{c} y\left(\ell \right)≜y_{c}\left(\frac{\ell }{2B}\right)=\int_{-\infty }^{\infty }g\left(\tau \right)u_{c}\left(\frac{\ell }{2B}-\tau \right)d\tau \end{array}$$

(27) $$\begin{array}{cc} & =\frac{1}{2B}\sum_{k=1}^{\infty }\sum_{i=0}^{\eta }g_{i}u\left(k\right)\int_{-\infty }^{\infty }\phi_{i}\left(\tau \right)\phi_{k}\left(\frac{\ell }{2B}-\tau \right)d\tau \end{array}$$

(28) $$\begin{array}{cc} & =\frac{1}{2B}\sum_{k=1}^{\infty }\sum_{i=0}^{\eta }g_{i}u\left(k\right)\int_{-\infty }^{\infty }\phi_{i}\left(\tau \right)\phi_{\ell -k}\left(\tau \right)d\tau \end{array}$$

(29) $$\begin{array}{cc} & =\sum_{i=0}^{\eta }g_{i}u\left(\ell -i\right), \end{array}$$

with u(k)=0 for k≤0. This means that the output samples $y_{1}^{\infty }$ are the discrete-time convolution between $u_{1}^{\infty }$ and the filter coefficients ${\left\{g_{i}\right\}}_{i=0}^{\eta }$. Therefore, the matrix relation (4) holds. We then obtain that $\bar{h}\left(y_{c}\right)=\bar{h}\left(u_{c}\right)+\log\left|g_{0}\right|$.

The frequency response of $G_{c}$ is given by

(30) $$\begin{array}{c} G_{c}\left(\xi \right)=\int_{-\infty }^{\infty }g\left(t\right)e^{-j2\pi \xi t}dt=\sum_{k=0}^{\eta }g_{k}\Phi_{k}\left(\xi \right)=\sum_{k=0}^{\eta }g_{k}e^{-j\pi \xi k/B}, \end{array}$$

where ξ is in [Hz]. This means that

(31) $$\begin{array}{c} g_{0}=\frac{1}{2B}\int_{-B}^{B}G_{c}\left(\xi \right)d\xi =\frac{1}{B}\int_{0}^{B}ℜ\left\{G_{c}\left(\xi \right)\right\}d\xi , \end{array}$$

where the last equality holds because $G_{c}\left(\xi \right)$ is conjugate symmetric. Thus, the entropy gain introduced by $G_{c}$ is the right-hand side of (13), concluding the proof.   □

### 3.2. Formalizing Shannon’s Argument

In the approach followed by Shannon, it is argued that the entropy gain is the limit as n→∞ of $n^{-1}\sum_{r=0}^{n-1}\log\left|G_{c}\left(\xi_{r}\right)\right|$ over uniformly spaced frequencies $\xi_{0},\ldots ,\xi_{n-1}$. Here, we show that this summation corresponds to $\log|\det({\tilde{G}}_{n})|$, where ${\tilde{G}}_{n}$ is an *n*-by-*n* Toeplitz circulant matrix. Moreover, the sequences of Hermitian matrices ${\left\{G_{n}G_{n}^{*}\right\}}_{n=1}^{\infty }$ and ${\left\{{\tilde{G}}_{n}{\tilde{G}}_{n}^{*}\right\}}_{n=1}^{\infty }$ are asymptotically equivalent (as defined in Reference [18], Section 2.3), which would yield $\lim_{n\to \infty }n^{-1}\log|\det\left(G_{n}\right)|=\lim_{n\to \infty }n^{-1}\log\left|\det\left({\tilde{G}}_{n}\right)\right|$ if the eigenvalues of $G_{n}G_{n}^{*}$ were bounded between constants $0<\zeta_{m}<\zeta_{M}<\infty$ for all n∈N. However, if G(z) (the *z*-transform of ${\left\{g_{k}\right\}}_{k=0}^{\infty }$) has NMP zeros, then $G_{n}G_{n}^{*}$ has eigenvalues tending to zero exponentially as n→∞, which precludes these two limits to coincide.

To prove the above claims, we first apply the change of variable ω≜πξ/B, with which (30) becomes

(32) $$\begin{array}{c} G_{c}\left(B\omega /\pi \right)=G\left(e^{j\omega }\right)≜\sum_{k=0}^{\eta }g_{k}e^{-j\omega k}, \end{array}$$

where $G(e^{j\omega })$ is the frequency response of the discrete-time filter *G* with unit-impulse response ${\left\{g_{i}\right\}}_{i=1}^{\eta }$ and ω is in radians per second. Now, following Shannon’s approach, we uniformly sample $G(e^{j\omega })$ at *n* frequencies

(33) $$\begin{array}{c} \omega_{r}≜\left\{\begin{array}{cc} r\frac{2\pi }{n} & ,\;r/n\leq 0.5\\ r\frac{2\pi }{n}-2\pi & ,\;r/n>0.5 \end{array}\right.\;\;\;\;,\;r=0,1,\ldots ,n-1, \end{array}$$

which, from (32), yields the spectral samples

(34) $$\begin{array}{cc} G(e^{-j\omega_{r}}) & =\sum_{k=0}^{\eta }g_{k}e^{-j\frac{2\pi }{n}rk}. \end{array}$$

We will cast the reason why (3) fails to coincide with the correct expression for the entropy gain provided by (5) as a disagreement between the asymptotic behavior of the logarithm of the determinant of two sequences of asymptotically equivalent matrices. For that purpose, since (34) coincides with Reference [18], Equation 4.34, we have that the spectral samples ${\left\{G(e^{-j\omega_{r}})\right\}}_{r=0}^{n-1}$ are the eigenvalues of the Toeplitz circulant matrix (Reference [18], Chapter 3)

(35) $$\begin{array}{cc} {\tilde{G}}_{n}≜\left(\begin{array}{cccc} {\tilde{g}}_{n,0} & {\tilde{g}}_{n,n-1} & \cdots & {\tilde{g}}_{n,1}\\ {\tilde{g}}_{n,1} & \ddots & \ddots & \vdots \\ \vdots & \ddots & \ddots & {\tilde{g}}_{n,n-1}\\ {\tilde{g}}_{n,n-1} & \ddots & \ddots & {\tilde{g}}_{n,0} \end{array}\right)\; & =U_{n}^{*}\mathrm{diag}\{G\left(e^{-j\omega_{0}}\right),\ldots ,G\left(e^{-j\omega_{n-1}}\right)\}U_{n}, \end{array}$$

where $U_{n}\in C^{n\times n}$ is the *n*-point *discrete Fourier transform* (DFT) matrix, defined as

(36) $$\begin{array}{c} {\left[U_{n}\right]}_{k,r}≜\frac{1}{\sqrt{n}}e^{-j\frac{2\pi }{n}kr},\;\;\;\;k,r=0,1,\ldots ,n-1. \end{array}$$

From Reference [18], Lemma 4.5, ${\tilde{g}}_{n,k}≜\sum_{i\in N_{0}:k+ni\leq \eta }g_{k+in}$, corresponding to the (possibly) aliased impulse response $g_{0},g_{1},\ldots ,g_{\eta }$ as a result of sampling in frequency.

We can now see that the discrepancy between the entropy gain predicted by (3) and (5) is the disagreement between the following limits:

(37a) $$\begin{array}{cc} \lim_{n\to \infty }\frac{1}{2n}\log\left(\det\left(G_{n}G_{n}^{*}\right)\right) & \overset{\left(31\right)}{=}\log\left|\frac{1}{B}\int_{0}^{B}ℜ\left\{G_{c}\left(\xi \right)\right\}d\xi \right|, \end{array}$$

(37b) limn→∞12nlog(det(G˜nG˜n*))=()1B∫0Blog|Gc(ξ)|dξ,

where, due to (8), the expressions on both right-hand sides differ if and only if G(z) has NMP zeros. According to Reference [18], Lemma 4.6, the sequences ${\left\{G_{n}\right\}}_{n=1}^{\infty }$ and ${\left\{{\tilde{G}}_{n}\right\}}_{n=1}^{\infty }$ are asymptotically equivalent, which is written as $G_{n}∼{\tilde{G}}_{n}$. Then, from Reference [18], Theorem 2.1, the Hermitian matrices $G_{n}G_{n}^{*}∼{\tilde{G}}_{n}{\tilde{G}}_{n}^{*}$, which, from Reference [18], Theorem 2.4, implies that

(38) $$\begin{array}{c} \lim_{n\to \infty }\frac{1}{n}\sum_{i=1}^{n}f\left(\lambda_{i}\left(G_{n}G_{n}^{*}\right)\right)=\lim_{n\to \infty }\frac{1}{n}\sum_{i=1}^{n}f\left(\lambda_{i}\left({\tilde{G}}_{n}{\tilde{G}}_{n}^{*}\right)\right), \end{array}$$

for any function *f* continuous over a finite interval $\left[\zeta_{m},\zeta_{M}\right]$ such that

(39) $$\begin{array}{c} \zeta_{m}\leq \lambda_{i}\left(G_{n}G_{n}^{*}\right),\lambda_{i}\left({\tilde{G}}_{n}{\tilde{G}}_{n}^{*}\right)\leq \zeta_{M},\;\;\;\;i=1,2,\ldots ,n,\;\;n=1,2,\ldots . \end{array}$$

However, when G(z) has *m* NMP zeros, Lemma 7 (in Section 6.3) establishes that there are exactly *m* eigenvalues of $G_{n}$ that tend to zero exponentially as n→∞. Crucially, log(·) is discontinuous at 0, which precludes the limits in (37) from coinciding.

## 4. The Effective Differential Entropy

Theorem 3 establishes that the effective differential entropy rate of the entire or complete output of an LTI system exceeds that of the (shorter) input sequence by the RHS of (15). This section provides a geometrical interpretation of this problem and intuition about the *effective differential entropy* already introduced in Definition 1.

Consider the random vectors $u≜{\left[u_{1}\;u_{2}\right]}^{T}$ and $y≜{\left[y_{1}\;y_{2}\;y_{3}\right]}^{T}$ related via

(40) $$\begin{array}{c} \left[\begin{array}{c} y_{1}\\ y_{2}\\ y_{3} \end{array}\right]=\underset{≜{\overset{˘}{G}}_{2}}{\underbrace{\left(\begin{array}{cc} 1 & 0\\ 2 & 1\\ 0 & 2 \end{array}\right)}}\left[\begin{array}{c} u_{1}\\ u_{2} \end{array}\right]. \end{array}$$

Suppose u is uniformly distributed over [0,1]×[0,1]. Applying the conventional definition of differential entropy of a random sequence, we would have that

(41) $$\begin{array}{c} h(y_{1},y_{2},y_{3})=h\left(y_{1},y_{2}\right)+h\left(y_{3}|y_{1},y_{2}\right)=-\infty \end{array}$$

because $y_{3}$ is a deterministic function of $y_{1}$ and $y_{2}$:

$$y_{3}=\left[0\;\;2\right]{\left[u_{1}\;u_{2}\right]}^{T}=\left[0\;\;2\right]{\left(\begin{array}{cc} 1 & 0\\ 2 & 1 \end{array}\right)}^{-1}\left[\begin{array}{c} y_{1}\\ y_{2} \end{array}\right].$$

**Figure 2:**
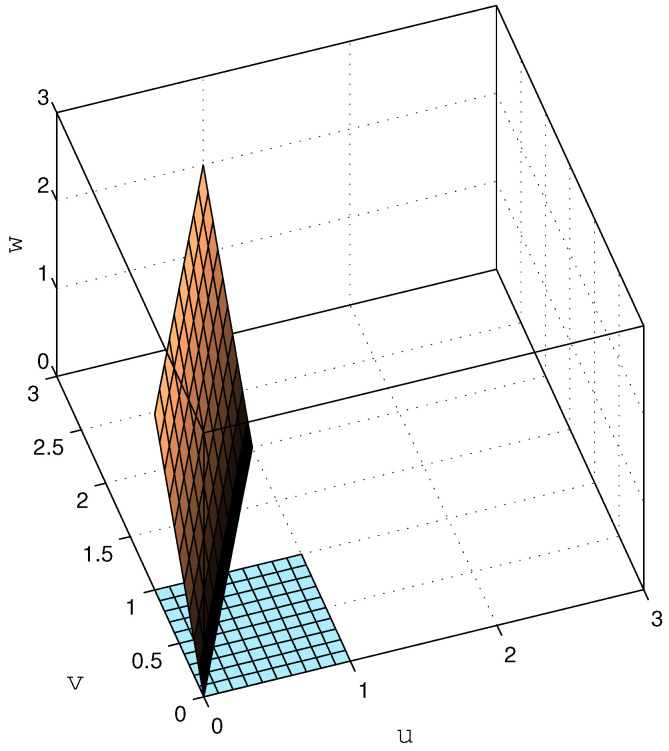
Support of u (laying in the u-v plane) compared to that of $y=\overset{˘}{G}u$ (the rhombus in $R^{3}$).

In other words, the problem lies in that, although the output is a three-dimensional vector, it only has two degrees of freedom, i.e., it is restricted to a 2-dimensional subspace of $R^{3}$. This is illustrated in Figure 2, where the set [0,1]×[0,1] is shown (coinciding with the u-v plane), together with its image through ${\overset{˘}{G}}_{2}$ (as defined in (40)).

As can be seen in this figure, the image of the square ${\left[0,1\right]}^{2}$ through ${\overset{˘}{G}}_{2}$ is a 2-dimensional rhombus over which $\left\{y_{1},y_{2},y_{3}\right\}$ distributes uniformly. Since the intuitive notion of differential entropy of an ensemble of random variables relates to the size of the region spanned by the associated random vector (and determines how difficult it is to compress it in a lossy fashion with a given precision), one could argue that the differential entropy of $\left\{y_{1},y_{2},y_{3}\right\}$, far from being −∞, should be somewhat larger than that of $\left\{u_{1},u_{2}\right\}$ (since the rhombus ${\overset{˘}{G}}_{2}{\left[0,1\right]}^{2}$ has a larger area than ${\left[0,1\right]}^{2}$). So, what does it mean that (and why should) $h(y_{1},y_{2},y_{3})=-\infty$? Simply put, the differential entropy relates to the volume spanned by the support of the probability density function. For y in our example, the latter (three-dimensional) volume is clearly zero.

From the above discussion, the comparison between the differential entries of $y\in R^{3}$ and $u\in R^{2}$ of our previous example should take into account that y actually lives in a two-dimensional subspace of $R^{3}$. Indeed, since the multiplication by a unitary matrix does not alter differential entries, we could consider the differential entropy of

(42) $$\begin{array}{c} \left[\begin{array}{c} \tilde{y}\\ 0 \end{array}\right]≜\left(\begin{array}{c} \overset{˘}{Q}\\ {\bar{q}}^{T} \end{array}\right)y, \end{array}$$

where ${\overset{˘}{Q}}^{T}$ is the 3×2 matrix with orthonormal rows in the SVD of ${\overset{˘}{G}}_{2}$

(43) $$\begin{array}{c} {\overset{˘}{G}}_{2}={\overset{˘}{Q}}^{T}\overset{˘}{D}\;\overset{˘}{R}, \end{array}$$

and $\bar{q}$ is a unit-norm vector orthogonal to the rows of $\overset{˘}{Q}$ (and thus orthogonal to y, as well). We are now able to compute the differential entropy in $R^{2}$ for $\tilde{y}$, corresponding to the rotated version of y such that its support is now aligned with $R^{2}$.

The preceding discussion motivates the use of a modified version of the notion of differential entropy for a random vector $y\in R^{n}$ which considers the number of dimensions actually spanned by y instead of its length.

It is worth mentioning that Shannon’s differential entropy of a vector $y\in R^{\ell }$, whose support’s *ℓ*-volume is greater than zero, arises from considering it as the difference between its (absolute) entropy and that of a random variable uniformly distributed over an *ℓ*-dimensional, unit-volume region of $R^{\ell }$. More precisely, if in this case the *probability density function* (PDF) of $y={\left[y_{1}\;y_{2}\;\cdots \;y_{\ell }\right]}^{T}$ is Riemann integrable, then [1], Thm. 9.3.1,

(44) $$\begin{array}{c} h\left(y\right)=\lim_{\Delta \to 0}\left[H(y^{\Delta })+\ell \log\Delta \right], \end{array}$$

where $y^{\Delta }$ is the discrete-valued random vector resulting when y is quantized using an *ℓ*-dimensional uniform quantizer with *ℓ*-cubic quantization cells with volume $\Delta^{\ell }$. However, if we consider a variable y whose support belongs to an *n*-dimensional subspace of $R^{\ell }$, n<ℓ (i.e., $y=Su=A^{T}TCu$, as in Definition 1), then the entropy of its quantized version in $R^{\ell }$, say $H_{\ell }\left(y^{\Delta }\right)$, is distinct from $H_{n}\left({\left(Ay\right)}^{\Delta }\right)$, the entropy of Ay in $R^{n}$. Moreover, it turns out that, in general,

(45) $$\begin{array}{c} \lim_{\Delta \to 0}\left(H_{\ell }\left(y^{\Delta }\right)-H_{n}\left({\left(Ay\right)}^{\Delta }\right)\right)\neq 0, \end{array}$$

despite the fact that A has orthonormal rows. Thus, the definition given by (44) does not yield consistent results for the case wherein a random vector has a support’s dimension (i.e., its number of degrees of freedom) smaller that its length (The mentioned inconsistency refers to (45).), which reveals that the asymptotic behavior $H_{\ell }\left(y^{\Delta }\right)$ changes if y is rotated. (If this were not the case, then we could redefine (44) replacing *ℓ* by *n*, in a spirit similar to the one behind Renyi’s *d*-dimensional entropy [19].) To see this, consider the case in which u∈R distributes uniformly over [0,1] and $y={\left[1\;1\right]}^{T}u/\sqrt{2}$. Clearly, y distributes uniformly over the unit-length segment connecting the origin with the point $\left(1,1\right)/\sqrt{2}$. Then,

(46) $$\begin{array}{c} H_{2}\left(y^{\Delta }\right)=-\left\lfloor \frac{1}{\Delta \sqrt{2}}\right\rfloor \Delta \sqrt{2}\log\left(\Delta \sqrt{2}\right)-\left(1-\left\lfloor \frac{1}{\Delta \sqrt{2}}\right\rfloor \sqrt{2}\Delta \right)\log\left(1-\left\lfloor \frac{1}{\Delta \sqrt{2}}\right\rfloor \sqrt{2}\Delta \right). \end{array}$$

On the other hand, since, in this case, Ay=u, we have that

(47) $$\begin{array}{c} H_{1}\left({\left(Ay\right)}^{\Delta }\right)=H_{1}\left(u^{\Delta }\right)=-\left\lfloor \frac{1}{\Delta }\right\rfloor \Delta \log\Delta -\left(1-\left\lfloor \frac{1}{\Delta }\right\rfloor \Delta \right)\log\left(1-\left\lfloor \frac{1}{\Delta }\right\rfloor \Delta \right). \end{array}$$

Thus, the *d*-dimensional entropy would not generally be equal to the effective differential entropy, that is:

(48) $$\begin{array}{c} \lim_{\Delta \to 0}\left(H_{1}\left({\left(Ay\right)}^{\Delta }\right)-H_{2}\left(y^{\Delta }\right)\right)=\lim_{\Delta \to 0}\left(\left\lfloor \frac{1}{\Delta \sqrt{2}}\right\rfloor \Delta \sqrt{2}\log\left(\Delta \sqrt{2}\right)-\left\lfloor \frac{1}{\Delta }\right\rfloor \Delta \log\Delta \right)=\log\sqrt{2}. \end{array}$$

The latter example further illustrates why the notion of effective entropy is appropriate in the setup considered in this section, where the effective dimension of the random sequences does not coincide with their length (it is easy to verify that the effective entropy of y does not change if one rotates y in $R^{\ell }$).

We finish this section with an example to illustrate the usefulness of the notion of effective differential entropy beyond the context of entropy gain.

### Application Example: Shannon Lower Bound

The rate-distortion function (RDF) R(D) is the infimum, among all codes, of the expected number of bits per sample necessary to reconstruct a given random source with distortion not greater than *D* [1]. Let the source and reconstruction be the vectors $x_{\ell }^{1}$ and $x_{\ell }^{1}+v_{\ell }^{1}$, respectively, and suppose the distortion is assessed using the *mean-squared error* (MSE) $d\left(v_{\ell }^{1}\right)≜E[∥v_{\ell }^{1}{∥}^{2}]$. Then, restricting our attention to uniquely-decodable codes Reference [1], p. 105), the *Shannon Lower Bound* (SLB) [20] establishes that

(49) $$\begin{array}{c} \ell R\left(D\right)\geq h\left(x_{\ell }^{1}\right)-\max_{d(v_{\ell }^{1})\leq D}h\left(v_{\ell }^{1}\right), \end{array}$$

provided $h(x_{\ell }^{1})$ is bounded. Therefore, if $x_{\ell }^{1}$ is the entire forced response of an FIR filter *G* of order *p* to an input $u_{1}^{n}$, then ℓ=n+p and $h(x_{\ell }^{1})$ is minus infinity, which precludes one from using (49). We will show next that, in this case, the SLB can still be stated by using the effective differential entropy $\overset{˘}{h}\left(x_{\ell }^{1}\right)$ instead of $h(x_{\ell }^{1})$. Following Definition 1, we can write the source vector as $x_{\ell }^{1}=A^{T}TCu_{n}^{1}$, where $A\in R^{n\times \ell }$ has orthonormal rows, $T\in R^{n\times n}$ is diagonal with non-negative entries, and $C\in R^{n\times n}$ is unitary. Let $H≜\left[A^{T}\;|\;{\bar{A}}^{T}\right]\in R^{\ell \times \ell }$ be a unitary matrix, which means that $\bar{A}A^{T}=0_{p\times \ell }$. Then,

(50) $$\begin{array}{cc} \ell R(D) & \overset{\left(a\right)}{\geq }I\left(x_{\ell }^{1};x_{\ell }^{1}+v_{\ell }^{1}\right), \end{array}$$

(51) =(a)I(Hxℓ1;Hxℓ1+Hvℓ1),

(52) =(a)I(Axℓ1;Axℓ1+Hvℓ1),

(53) =(a)I(Axℓ1;Axℓ1+Avℓ1,A¯vℓ1),

(54) $$\begin{array}{cc} & \overset{\left(21\right)}{=}I\left(Ax_{\ell }^{1};Ax_{\ell }^{1}+Av_{\ell }^{1}\right),+I\left(Ax_{\ell }^{1};\bar{A}v_{\ell }^{1}\;|\;Ax_{\ell }^{1}+Av_{\ell }^{1}\right), \end{array}$$

(55) $$\begin{array}{cc} & \overset{\left(20\right)}{\geq }I\left(Ax_{\ell }^{1};Ax_{\ell }^{1}+Av_{\ell }^{1}\right), \end{array}$$

(56) $$\begin{array}{cc} & \overset{\left(22\right)}{=}h\left(Ax_{\ell }^{1}\right)-h\left(Ax_{\ell }^{1}|Ax_{\ell }^{1}+Av_{\ell }^{1}\right), \end{array}$$

(57) $$\begin{array}{cc} & \overset{\left(19\right)}{=}h\left(Ax_{\ell }^{1}\right)-h\left(Av_{\ell }^{1}|Ax_{\ell }^{1}+Av_{\ell }^{1}\right), \end{array}$$

(58) $$\begin{array}{cc} & \overset{\left(b\right)}{\geq }h\left(Ax_{\ell }^{1}\right)-h\left(Av_{\ell }^{1}\right), \end{array}$$

(59) ≥(a)h(Axℓ1)−maxwn1:d(wn1)≤Dh(wn1),

(60) $$\begin{array}{cc} & \overset{\left(c\right)}{=}\overset{˘}{h}\left(x_{\ell }^{1}\right)-\max_{w_{n}^{1}:d\left(w_{n}^{1}\right)\leq D}h\left(w_{n}^{1}\right), \end{array}$$

where (a) stems from Reference [1], Theorems 5.4.1 and 5.5.1 and Equations (10).58-10.61, (b) holds because conditioning does not increase entropy and (c) is from the definition of effective differential entropy.

## 5. Entropy-Balanced Processes: Geometric Interpretation and Properties

In the first part of this section, we provide a geometric interpretation of the effect that a non-minimum phase LTI system has on its input random process. This will give an intuitive meaning to the notion of an entropy-balanced random process (introduced in Definition 2 above) and provide insights into why and how the entropy gain defined in (1) arises as a consequence of an output random disturbance or a random initial state (the themes of Section 6 and Section 7, respectively).

The second part of this section identifies several entropy-balanced processes and establishes two properties satisfied by this class of processes.

### 5.1. Geometric Interpretation

We begin our discussion with a simple example.

**Example** **1.** *Suppose that G in Figure 1 is a finite impulse response (FIR) filter with impulse response $g_{0}=1,\;g_{1}=2,\;g_{i}=0,\;\forall i\geq 2$. Notice that this choice yields G(z)=(z−2)/z; thus, G(z) has one non-minimum phase zero, at z=2. The associated matrix $G_{n}$ for n=3 is*

$$G_{3}=\left(\begin{array}{ccc} 1 & 0 & 0\\ 2 & 1 & 0\\ 0 & 2 & 1 \end{array}\right),$$

**Figure 3:**
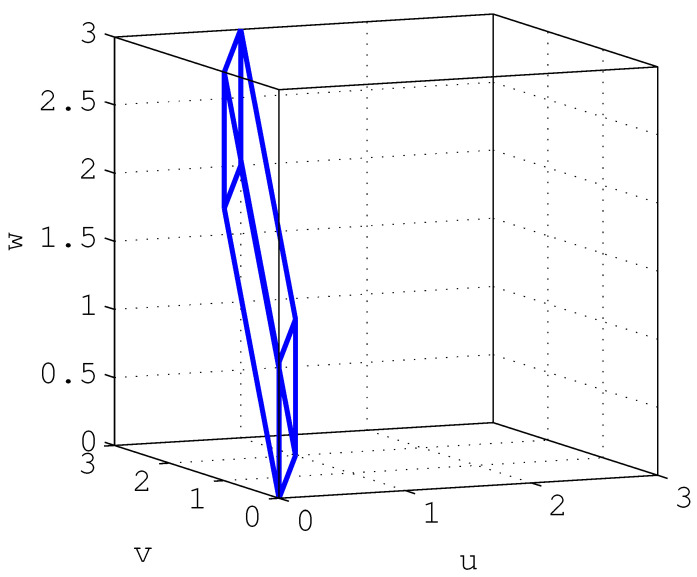
Image of the cube ${\left[0,1\right]}^{3}$ through the square matrix with columns ${\left[1\;2\;0\right]}^{T}$, ${\left[0\;1\;2\right]}^{T}$, and ${\left[0\;0\;1\right]}^{T}$.

*whose determinant is clearly one (indeed, all its eigenvalues are 1). Hence, as discussed in the introduction, $h\left(G_{3}u_{3}^{1}\right)=h\left(u_{3}^{1}\right)$; thus, $G_{3}$ (and $G_{n}$, in general) does not introduce an entropy gain by itself. However, an interesting phenomenon becomes evident by looking at the SVD of $G_{3}$, given by $G_{3}=Q_{3}^{T}D_{3}R_{3}$, where $Q_{3}$ and $R_{3}$ are unitary matrices, and $D_{3}≜\mathrm{diag}\left\{d_{1},d_{2},d_{3}\right\}$. In this case, $D_{3}=\mathrm{diag}\left\{0.19394,\;1.90321,\;2.70928\right\}$; thus, one of the singular values of $G_{3}$ is much smaller than the others (although the product of all singular values yields 1, as expected). As will be shown in Section 6, for a stable G(z), such uneven distribution of singular values arises only when G(z) has non-minimum phase zeros. The effect of this can be visualized by looking at the image of the cube ${\left[0,1\right]}^{3}$ through $G_{3}$, shown in Figure 3.*

*If the input $u_{3}^{1}$ were uniformly distributed over this cube (of unit volume), then $G_{3}u_{3}^{1}$ would distribute uniformly over the unit-volume parallelepiped depicted in Figure 3; hence, $h\left(G_{3}u_{3}^{1}\right)=h\left(u_{3}^{1}\right)$.*

*Now, if we add to $G_{3}u_{3}^{1}$ a disturbance $z_{3}^{1}=\Phi s$, with scalar s uniformly distributed over [−0.5,0.5] independent of $u_{3}^{1}$, and with $\Phi \in R^{3\times 1}$, the effect would be to “thicken” the support over which the resulting random vector $y_{3}^{1}=G_{3}u_{3}^{1}+z_{3}^{1}$ is distributed, along the direction pointed by Φ. If Φ is aligned with the direction along which the support of $G_{3}u_{3}^{1}$ is thinnest (given by $q_{3,1}$, the first row of $Q_{3}$), then the resulting support would have its volume significantly increased, which can be associated with a large increase in the differential entropy of $y_{3}^{1}$ with respect to $u_{3}^{1}$. Indeed, a relatively small variance of s and an approximately aligned Φ would still produce a significant entropy gain.*

The above example suggests that the entropy gain from $u_{n}^{1}$ to $y_{n}^{1}$ appears as a combination of two factors. The first of these is the uneven way in which the random vector $G_{n}u_{n}^{1}$ is distributed over $R^{n}$. The second factor is the alignment of the disturbance vector $z_{n}^{1}$ with respect to the span of the subset ${\left\{q_{n,i}\right\}}_{i\in \Omega_{n}}$ of columns of $Q_{n}$, associated with the smallest singular values of $G_{n}$, indexed by the elements in the set $\Omega_{n}$. As we shall discuss in the next section, if *G* has *m* non-minimum phase zeros, then, as *n* increases, there will be *m* singular values of $G_{n}$ going to zero exponentially. Since the product of the singular values of $G_{n}$ equals 1 for all *n*, it follows that $\prod_{i\notin \Omega_{n}}d_{n,i}$ must grow exponentially with *n*, where $d_{n,i}$ is the *i*-th diagonal entry of $D_{n}$. This implies that $G_{n}u_{n}^{1}$ expands with *n* along the span of ${\left\{q_{n,i}\right\}}_{i\notin \Omega_{n}}$, compensating its shrinkage along the span of ${\left\{q_{n,i}\right\}}_{i\in \Omega_{n}}$, thus keeping $h\left(G_{n}u_{n}^{1}\right)=h\left(u_{n}^{1}\right)$ for all *n*. Thus, as *n* grows, any small disturbance distributed over the span of ${\left\{q_{n,i}\right\}}_{i\in \Omega_{n}}$, added to $G_{n}u_{n}^{1}$, will keep the support of the resulting distribution from shrinking along this subspace. Consequently, the expansion of $G_{n}u_{n}^{1}$ with *n* along the span of ${\left\{q_{n,i}\right\}}_{i\notin \Omega_{n}}$ is no longer compensated, yielding an entropy increase proportional to $\log(\prod_{i\notin \Omega_{n}}d_{n,i})$.

The above analysis allows one to anticipate a situation in which no entropy gain would take place even when some singular values of $G_{n}$ tend to zero as n→∞. Since the increase in entropy is made possible by the fact that, as *n* grows, the support of the distribution of $G_{n}u_{n}^{1}$ shrinks along the span of ${\left\{q_{n,i}\right\}}_{i\in \Omega_{n}}$, no such entropy gain should arise if the support of the distribution of the input $u_{n}^{1}$ expands accordingly along the directions pointed by the rows ${\left\{r_{n,i}\right\}}_{i\in \Omega_{n}}$ of $R_{n}$.

An example of such situation can be easily constructed as follows: Let G(z) in Figure 1 have non-minimum phase zeros and suppose that $u_{1}^{\infty }$ is generated as $G^{-1}{\tilde{u}}_{1}^{\infty }$, where ${\tilde{u}}_{1}^{\infty }$ is an i.i.d. random process with bounded entropy rate. Since the determinant of $G_{n}^{-1}$ equals 1 for all *n*, we have that $h\left(u_{n}^{1}\right)=h\left({\tilde{u}}_{n}^{1}\right)$, for all *n*. On the other hand, $y_{n}^{1}=G_{n}G_{n}^{-1}{\tilde{u}}_{n}^{1}+z_{n}^{1}={\tilde{u}}_{n}^{1}+z_{n}^{1}$. Since $z_{n}^{1}={\left[\Phi \right]}_{n}^{1}s_{\kappa }^{1}$ for some finite κ (recall Assumption 2), it is easy to show that $\lim_{n\to \infty }\frac{1}{n}h\left(y_{n}^{1}\right)=\lim_{n\to \infty }\frac{1}{n}h\left({\tilde{u}}_{n}^{1}\right)=\lim_{n\to \infty }\frac{1}{n}h\left(u_{n}^{1}\right)$; thus, no entropy gain appears.

The preceding discussion reveals that the entropy gain produced by *G* in the situation shown in Figure 1 **depends on the distribution of the input and on the support and distribution of the disturbance**. This stands in stark contrast with the well known fact that the increase in differential entropy produced by an invertible linear operator depends only on its Jacobian, and not on the statistics of the input [2]. We have also seen that the distribution of a random process along the different directions within the Euclidean space which contains it plays a key role, as well. This motivates the need to specify a class of random processes which distribute more or less evenly over all directions. This is precisely the intuitive meaning of an entropy-balanced process.

The following section identifies a large family of processes belonging to this class, as well as two properties which greatly expands this family.

### 5.2. Characterization of Entropy-Balanced Processes

We have defined the notion of an “entropy-balanced” process in Section 1.1. In words, the first condition in this definition allows one to guarantee that the orthogonal projection of an entropy-balanced process onto any ν-dimensional linear subspace has a differential entropy whose magnitude remains bounded or grows at most sub-linearly with *n*. The second condition states that the projection of an entropy-balanced process $v_{1}^{\infty }$ onto any linear subspaces having ν fewer dimensions has the same differential entropy rate as the original process. This condition is equivalent to requiring that every unitary transformation on $v_{1}^{n}$ yields a random sequence $y_{1}^{n}$ such that $\lim_{n\to \infty }\frac{1}{n}h\left(y_{n-\nu +1}^{n}|y_{1}^{n-\nu }\right)=0$. This property of the resulting random sequence $y_{1}^{n}$ means that one cannot predict its last ν samples with arbitrary accuracy by using its previous n−ν samples, even if *n* goes to infinity.

We now characterize a large family of entropy-balanced random processes and establish some of their properties. Although intuition may suggest that most random processes (such as i.i.d. or stationary processes) should be entropy balanced, that statement seems rather difficult to prove. In the following, we show that the entropy-balanced condition is met by i.i.d. processes with per-sample *probability density function* (PDF) being uniform, piece-wise constant or Gaussian. It is also shown that adding to an entropy-balanced process an independent random processes independent of the former yields another entropy-balanced process, and that filtering an entropy-balanced process by a stable and minimum phase filter yields an entropy-balanced process, as well. The proofs can be found in Appendix B.

**Lemma** **1.** *Let $u_{1}^{\infty }$ be a Gaussian random process with independent elements having positive and bounded variance, i.e., there exist $0<{\overset{ˇ}{\sigma }}^{2}\leq {\hat{\sigma }}^{2}<\infty$ such that ${\overset{ˇ}{\sigma }}^{2}\leq \sigma_{u(n)}^{2}\leq {\hat{\sigma }}^{2}$, n∈N. Then, $u_{1}^{\infty }$ is entropy balanced.*

**Lemma** **2.** *Let $u_{1}^{\infty }$ be a random process with independent elements satisfying Condition i) in Definition 2, in which each $u_{i}$ is distributed according to a (possibly different) piece-wise constant PDF such that each interval where this PDF is constant has measure less than θ and greater than ϵ, for some constants 0<ϵ<θ<∞. Then, $u_{1}^{\infty }$ is entropy balanced.*

**Lemma** **3.** *Let $u_{1}^{\infty }$ and $v_{1}^{\infty }$ be mutually independent random processes. If $u_{1}^{\infty }$ is entropy balanced, and $w_{1}^{\infty }≜u_{1}^{\infty }+v_{1}^{\infty }$ satisfies $\sigma_{w(n)}^{2}<\infty$ for finite n and $\lim_{n\to \infty }n^{-1}\log\left(\sigma_{w(n)}^{2}\right)=0$, then $w_{1}^{\infty }$ is also entropy balanced.*

The proof of Lemma 3 is on page 33. The working behind this lemma can be interpreted intuitively by noting that adding to a random process another independent random process can only increase the “spread” of the distribution of the former, which tends to balance the entropy of the resulting process along all dimensions in Euclidean space. In addition, it follows from Lemma 3 that all i.i.d. processes having a per-sample PDF which can be constructed by convolving uniform, piece-wise constant or Gaussian PDFs as many times as required are entropy balanced. It also implies that one can have non-stationary processes which are entropy balanced, since Lemma 3 imposes no requirements for the process $v_{1}^{\infty }$.

The next lemma related to the properties of entropy-balanced processes shows that filtering by a stable and minimum phase LTI filter preserves the entropy balanced condition of its input.

**Lemma** **4.** *Let $u_{1}^{\infty }$ be an entropy-balanced process and G an LTI stable and minimum-phase filter. Then, the output $w_{1}^{\infty }≜Gu_{1}^{\infty }$ is also an entropy-balanced process.*

This result implies that any stable moving-average auto-regressive process constructed from entropy-balanced innovations is also entropy balanced, provided the coefficients of the averaging and regression correspond to a stable MP filter.

The last lemma of this section states a crucial property of entropy-balanced processes (the proof is in Appendix B, page 34).

**Lemma** **5.** *Let $u_{1}^{\infty }$ be an entropy balanced process. Consider a disturbance $z_{1}^{\infty }$ satisfying Assumption 2 and define $y_{1}^{\infty }≜u_{1}^{\infty }+z_{1}^{\infty }$. Then, $\lim_{n\to \infty }n^{-1}\left(h\left(y_{1}^{n}\right)-h\left(u_{1}^{n}\right)\right)=0$.*

We finish this section by pointing out two examples of processes which are non-entropy-balanced, namely the output of a NMP-filter to an entropy-balanced input and the output of an unstable filter to an entropy-balanced input. The first of these cases plays a central role in the next section.

## 6. Entropy Gain Due to External Disturbances

In this section, we formalize the ideas which were qualitatively outlined in the previous section. Specifically, for the system shown in Figure 1 we will characterize the entropy gain $G(G,x_{0},u_{1}^{\infty },z_{1}^{\infty })$ defined in (1) for the case in which the initial state $x_{0}$ is zero (or deterministic) and there exists a random disturbance of (possibly infinite length) $z_{1}^{\infty }$ which satisfies Assumption 2.

### 6.1. Input Disturbances Do Not Produce Entropy Gain

In this section, we show that random disturbances satisfying Assumption 2, when added to the *input* $u_{1}^{\infty }$ (i.e., before *G*), do not introduce entropy gain. This result can be obtained from Lemma 6, as stated in the following theorem:

**Theorem** **5** (Input Disturbances do not Introduce Entropy Gain)**.** *Let G and $z_{1}^{\infty }$ satisfy Assumptions 1 and 2, respectively. Suppose that $u_{1}^{\infty }$ is entropy balanced and consider the output*

(61) $$\begin{array}{c} y_{1}^{\infty }=G\;\left(u_{1}^{\infty }+z_{1}^{\infty }\right). \end{array}$$

*Then,*

(62) $$\begin{array}{c} \lim_{n\to \infty }\frac{1}{n}\left(h\left(y_{1}^{n}\right)-h\left(u_{1}^{n}\right)\right)=0 \end{array}$$

**Proof.**  From Lemma 5, the differential entropy rate of $u_{1}^{\infty }$ equals that of $u_{1}^{\infty }+z_{1}^{\infty }$. The proof is completed by recalling that *G* yields no entropy gain for its input $u_{1}^{\infty }+z_{1}^{\infty }$ because it corresponds to the noise-less scenario.    □

### 6.2. The Entropy Gain Introduced by Output Disturbances when *G* is MP is Zero

The results from the previous section yield the following corollary, which states that an LTI system with transfer function G(z) without zeros outside the unit circle (i.e., an MP transfer function) cannot introduce entropy gain.

**Corollary** **1** (Minimum Phase Filters do not Introduce Entropy Gain)**.** *Consider the system shown in Figure 1 wherein the input $u_{1}^{\infty }$ is an entropy-balanced random process and the output disturbance $z_{1}^{\infty }$ satisfies Assumption 2. Besides Assumption 1, suppose that G(z) is minimum phase. Then,*

(63) $$\begin{array}{c} \lim_{n\to \infty }\frac{1}{n}\left(h\left(y_{1}^{n}\right)-h\left(u_{1}^{n}\right)\right)=0. \end{array}$$

**Proof.**  Since G(z) is minimum phase and stable, the result follows directly from Lemmas 4 and 5.    □

### 6.3. The Entropy Gain Introduced by Output Disturbances when G(z) is NMP

We show here that the entropy gain of an LTI system with transfer function G(z) and an output disturbance is at most the sum of the logarithm of the magnitude of the zeros of G(z) outside the unit circle.

The following lemma will be instrumental for that purpose.

**Lemma** **6.** *Consider the system in Figure 1, and suppose $z_{1}^{\infty }$ satisfies Assumption 2, and that the input process $u_{1}^{\infty }$ is entropy balanced. Let $G_{n}=Q_{n}^{T}D_{n}R_{n}$ be the SVD of $G_{n}$, where $D_{n}=\mathrm{diag}\left\{d_{n,1},\ldots ,d_{n,n}\right\}$ are the singular values of $G_{n}$, with $d_{n,1}\leq d_{n,2}\leq \cdots \leq d_{n,n}$, such that $|\det G_{n}|=1$∀n. Let m be the number of these singular values which tend to zero exponentially as n→∞. Then,*

(64) $$\begin{array}{c} \lim_{n\to \infty }\frac{1}{n}\left(h\left(y_{1}^{n}\right)-h\left(u_{1}^{n}\right)\right)=\lim_{n\to \infty }\frac{1}{n}\left(-\sum_{i=1}^{m}\log d_{n,i}+h\left({\left[D_{n}\right]}_{m}^{1}R_{n}u_{n}^{1}+{\left[Q_{n}\right]}_{m}^{1}z_{n}^{1}\right)\right). \end{array}$$

The proof of this lemma can be found on page 34, in Appendix B.

Lemma 6 leaves the need to characterize the asymptotic behavior of the singular values of $G_{n}$. This is accomplished in the following lemma, which relates these singular values to the zeros of G(z). It is a generalization of the unnumbered lemma in the proof of Reference [16], Theorem 1 (restated in Appendix C as Lemma A3), which holds for FIR transfer functions, to the case of *infinite-impulse response* (IIR) transfer functions (i.e., transfer functions having poles).

**Lemma** **7.** *For a transfer function G(z) satisfying Assumption 1, where its zeros ${\left\{\rho_{i}\right\}}_{i=1}^{p}$ satisfy $|\rho_{1}\left|\geq \cdots \geq \right|\rho_{m}\left|>1\geq \right|\rho_{m+1}\left|\geq \cdots \geq \right|\rho_{p}|$. Then,*

(65) $$\begin{array}{c} \lambda_{l}\left(G_{n}G_{n}^{T}\right)=\left\{\begin{array}{cc} \alpha_{n,l}^{2}{\left|\rho_{l}\right|}^{-2n} & ,\mathrm{if}\;l\leq m,\\ \alpha_{n,l}^{2} & ,otherwise\;, \end{array}\right. \end{array}$$

*where the elements in the sequence $\left\{\alpha_{n,l}\right\}$ are positive and increase or decrease at most polynomially with n.*

(The proof of this lemma can be found in Appendix B, page 36).

Lemma 6 also precisely formulates the geometric idea outlined in Section 5.1. To see this, notice that no entropy gain is obtained if the output disturbance vector $z_{n}^{1}$ becomes orthogonal (with probability 1) to the space spanned by the first *m* columns of $Q_{n}$ sufficiently fast as n→∞. Recalling from Assumption 2 that

(66) $$\begin{array}{c} z_{n}^{1}={\left[\Phi \right]}_{n}^{1}s_{\kappa }^{1}, \end{array}$$

where the matrix Φ has κ orthonormal columns of infinite length, such orthogonality condition can be formally stated by defining

(67) $$\begin{array}{c} \kappa_{n}≜\mathrm{rank}({\left[Q_{n}\right]}_{m}^{1}{\left[\Phi \right]}_{n}^{1}) \end{array}$$

(68) $$\begin{array}{c} {\hat{\kappa }}_{\infty }≜\underset{n\to \infty }{\lim\sup}\kappa_{n} \end{array}$$

(69) $$\begin{array}{c} {\overset{ˇ}{\kappa }}_{\infty }≜\underset{n\to \infty }{\lim\inf}\kappa_{n} \end{array}$$

as $\hat{\kappa }=0$.

If this were the case, then the disturbance would not be able fill the subspace along which $G_{n}u_{n}^{1}$ is shrinking exponentially. Indeed, if $\kappa_{n}=0$ for all *n*, then $h\left({\left[D_{n}\right]}_{m}^{1}R_{n}u_{n}^{1}+{\left[Q_{n}\right]}_{m}^{1}z_{n}^{1}\right)=h\left({}^{1}\;{\left[D_{n}\right]}_{m}{\left[R_{n}\right]}_{m}^{1}u_{n}^{1}\right)=\sum_{i=1}^{m}\log d_{n,i}+h\left({\left[R_{n}\right]}_{m}^{1}u_{n}^{1}\right)$, and the latter sum cancels out the one on the RHS of (64), while $\lim_{n\to \infty }\frac{1}{n}h\left({\left[R_{n}\right]}_{m}^{1}u_{n}^{1}\right)=0$ since $u_{1}^{\infty }$ is entropy balanced. On the contrary (and loosely speaking), if the projection of the support of $z_{n}^{1}$ onto the subspace spanned by the first *m* rows of $Q_{n}$ is of dimension *m* (i.e., if $\kappa_{n}=m$) for all *n*, then $h({\left[D_{n}\right]}_{m}^{1}R_{n}u_{n}^{1}+{\left[Q_{n}\right]}_{m}^{1}z_{n}^{1})$ remains bounded for all *n*, and the entropy limit of the sum $\lim_{n\to \infty }\frac{1}{n}\left(-\sum_{i=1}^{m}\log d_{n,i}\right)$ on the RHS of (64) yields the largest possible entropy gain. Notice that $-\sum_{i=1}^{m}\log d_{n,i}=\sum_{i=m+1}^{n}\log d_{n,i}$ (because $\det(G_{n})=1$); thus, this entropy gain stems from the uncompensated expansion of $G_{n}u_{n}^{1}$ along the space spanned by the rows of ${\left[Q_{n}\right]}_{n}^{m+1}$. Beyond these extreme cases (i.e., for general values of $\overset{ˇ}{\kappa }$ and $\hat{\kappa }$), the following theorem provides tight bounds on the entropy gain.

**Theorem** **6.** *In the system of Figure 1, suppose that $u_{1}^{\infty }$ is entropy balanced, and that G(z) and $z_{1}^{\infty }$ satisfy Assumptions 1 and 2, respectively, where the zeros ${\left\{\rho_{i}\right\}}_{i=1}^{p}$ of G(z) satisfy $|\rho_{1}\left|\geq \cdots \geq \right|\rho_{m}\left|>1\geq \right|\rho_{m+1}\left|\geq \cdots \geq \right|\rho_{p}|$. For each n∈N, let $Q_{n}^{T}\in R^{n\times n}$ be the unitary matrix holding the left singular vectors of $G_{n}\in R^{n\times n}$ (as in Lemma 6), where $G_{n}$ is as defined in (4).*

*1*. *Then,*
  (70) $$\begin{array}{c} 0\leq \underset{n\to \infty }{\lim\inf}\frac{1}{n}\left(h\left(y_{1}^{n}\right)-h\left(u_{1}^{n}\right)\right)\leq \underset{n\to \infty }{\lim\sup}\frac{1}{n}\left(h\left(y_{1}^{n}\right)-h\left(u_{1}^{n}\right)\right)\leq \sum_{i=1}^{{\hat{\kappa }}_{\infty }}\log\left|\rho_{i}\right|\overset{\left(8\right)}{\leq }\frac{1}{2\pi }\;\;\int_{-\pi }^{\pi }\log\left|G(e^{j\omega })\right|d\omega . \end{array}$$
*The bounds on both extremes are tight. Moreover, the lower bound is reached if ${\hat{\kappa }}_{\infty }=0$.*
*2*. *If ${\lim\inf}_{n\to \infty }\frac{1}{n}\log(\sigma_{\min}\left({\left[Q_{n}\right]}_{m}^{1}{\left[\Phi \right]}_{n}^{1}\right)=0$, then*
  (71) $$\begin{array}{c} \sum_{i=m-{\overset{ˇ}{\kappa }}_{\infty }+1}^{m}\log\left|\rho_{i}\right|\leq \underset{n\to \infty }{\lim\inf}\frac{1}{n}\left(h\left(y_{1}^{n}\right)-h\left(u_{1}^{n}\right)\right). \end{array}$$
*Thus, the rightmost upper bound in (70) is achieved if ${\overset{ˇ}{\kappa }}_{\infty }=m$.*

**Proof.**  See Appendix B, page 37.    □

The next technical result is very useful for finding conditions under which the requirements of point 2 in Theorem 6 are satisfied (the proof is in Appendix B, page 39).

**Lemma** **8.** *Let F be an FIR LTI causal system of order m such that the m zeros of F(z) are NMP, and $F_{n}=Q_{n}^{T}D_{n}R_{n}$ be an SVD for $F_{n}$, for every n∈{m,m+1,…}. For each κ∈{1,…,n}, define*

(72) $$\begin{array}{c} \kappa_{n}≜\mathrm{rank}\left(\overset{1\;\;\kappa }{\overset{⎴}{{\left[Q_{n}\right]}_{m}^{1}}}\right), \end{array}$$

*and $\bar{\kappa }≜\min\left\{m,\kappa \right\}$. Then,*

(73) $$\begin{array}{c} \lim_{n\to \infty }\sigma_{\min}\left(\overset{1\;\;\kappa }{\overset{⎴}{{\left[Q_{n}\right]}_{m}^{1}}}\right)>0, \end{array}$$

*and $\lim_{n\to \infty }\kappa_{n}=\bar{\kappa }$.*

Now, we can prove Theorem 4.

### 6.4. Proof of Theorem 4

Factorize G(z) as $G\left(z\right)=F\left(z\right)\tilde{G}\left(z\right)$, where $\tilde{G}\left(z\right)$ is stable and minimum phase and F(z) is a stable FIR transfer function with all the *m* non-minimum-phase zeros of G(z). Letting ${\tilde{u}}_{n}^{1}≜{\tilde{G}}_{n}u_{n}^{1}$, we have that $h\left(y_{n}^{1}\right)=h\left(F_{n}{\tilde{u}}_{n}^{1}+z_{n}^{1}\right)$, $h\left({\tilde{u}}_{n}^{1}\right)=h\left(u_{n}^{1}\right)$, and that ${\tilde{u}}_{1}^{\infty }$ is entropy balanced (from Lemma 4). Thus,

(74) $$\begin{array}{c} h\left(y_{n}^{1}\right)-h\left(u_{n}^{1}\right)=h\left(G_{n}u_{n}^{1}+z_{n}^{1}\right)-h\left(u_{n}^{1}\right)=h\left(F_{n}{\tilde{u}}_{n}^{1}+z_{n}^{1}\right)-h\left({\tilde{u}}_{n}^{1}\right). \end{array}$$

This means that the entropy gain of *G* due to the output disturbance $z_{1}^{\infty }$ corresponds to the entropy gain of *F* due to the same output disturbance.

Clearly, $u_{1}^{\infty }$, F(z), and $z_{1}^{\infty }$ satisfy the assumptions of Theorem 6 with $\Phi ={\left[\;I_{\kappa }\;|\;0\;\right]}^{T}$ (see Assumption 2). Therefore,

(75) $$\begin{array}{c} {\left[Q_{n}\right]}_{m}^{1}{\left[\Phi \right]}_{n}^{1}=\overset{1\;\;\kappa }{\overset{⎴}{{\left[Q_{n}\right]}_{m}^{1}}}. \end{array}$$

Combining this with Lemma 8, it readily follows that, for every κ≥1, the condition in point 2 of Theorem 6 is met, and also $\lim_{n\to \infty }\kappa_{n}=\bar{\kappa }$. The proof is then completed by substituting ${\lim\inf}_{n\to \infty }\kappa_{n}={\lim\sup}_{n\to \infty }\kappa_{n}=\bar{\kappa }$ into (70) and (71).

## 7. Entropy Gain Due to a Random Initial State

Here, we analyze the scenario illustrated by Figure 1 for the case in which there exists a random initial state $x_{0}$ independent of the input $u_{1}^{\infty }$, and zero (or deterministic) output disturbance.

The treatment of an initial state of the LTI system *G* requires one to first define an internal model for it. For this purpose, in this section, we consider the state-space realization of *G* in the Kalman canonical form, given by

(76a) $$\begin{array}{c} x\left(k\right)≜\left[\begin{array}{c} x_{co}\left(k\right)\\ x_{\bar{c}o}\left(k\right)\\ x_{c\bar{o}}\left(k\right)\\ x_{\bar{c}\bar{o}}\left(k\right), \end{array}\right]=\left(\begin{array}{cccc} A_{co} & A_{12} & 0 & 0\\ 0 & A_{\bar{c}o} & 0 & 0\\ A_{31} & A_{32} & A_{c\bar{o}} & A_{34}\\ 0 & A_{42} & 0 & A_{\bar{c}\bar{o}} \end{array}\right)\left[\begin{array}{c} x_{co}\left(k-1\right)\\ x_{\bar{c}o}\left(k-1\right)\\ x_{c\bar{o}}\left(k-1\right)\\ x_{\bar{c}\bar{o}}\left(k-1\right), \end{array}\right]+\left[\begin{array}{c} b_{co}\\ 0\\ b_{c\bar{o}}\\ 0 \end{array}\right]u\left(k\right) \end{array}$$

(76b) $$\begin{array}{c} y(k)=\left[\begin{array}{cccc} c_{co}^{T} & c_{\bar{c}o}^{T} & 0 & 0 \end{array}\right]x\left(k-1\right)+u\left(k\right), \end{array}$$

(see, e.g., Reference [21] or Reference [22], Chapter 6) where the column state vectors $x_{co}\left(k\right)$, $x_{\bar{c}o}\left(k\right)$, $x_{c\bar{o}}\left(k\right)$, $x_{\bar{c}\bar{o}}\left(k\right)$ are, respectively, *controllable and observable*, *non-controllable and observable*, *controllable and non-observable*, and *non-controllable and non-observable*. There is no loss of generality in choosing this state-space representation, because every state-space representation consistent with a rational transfer function G(z) can be written in this form (Reference [22], Theorem 6.7).

Since our interest is on the effect of the random initial state of *G* on its output, we only need to consider the observable subsystem within (76) and without its input, given by

(77a) $$\begin{array}{cc} x_{o}\left(k\right)≜\left[\begin{array}{c} x_{co}\left(k\right)\\ x_{\bar{c}o}\left(k\right) \end{array}\right] & =\underset{A_{o}}{\underbrace{\left(\begin{array}{cc} A_{co} & A_{12}\\ 0 & A_{\bar{c}o} \end{array}\right)}}\left[\begin{array}{c} x_{co}\left(k-1\right)\\ x_{\bar{c}o}\left(k-1\right) \end{array}\right], \end{array}$$

(77b) $$\begin{array}{cc} \tilde{y}\left(k\right) & =\underset{c_{o}^{T}}{\underbrace{\left[c_{co}^{T}\;\;c_{\bar{c}o}^{T}\right]}}x_{o}\left(k-1\right), \end{array}$$

where $\tilde{y}$ is the natural response of *G* to its initial state $x_{o}\left(0\right)$ and $x_{co}\in R^{p}$ and $x_{\bar{c}o}\in R^{q}$. We shall decompose $\tilde{y}$ as

(78) $$\begin{array}{c} {\tilde{y}}_{1}^{\infty }={\left[{\tilde{y}}_{\bar{c}o}\right]}_{1}^{n}+{\left[{\tilde{y}}_{co}\right]}_{1}^{\infty }, \end{array}$$

where ${\tilde{y}}_{\bar{c}o}$ and ${\tilde{y}}_{co}$ are the natural responses of *G* to initial states ${\left[0_{1\times p}\;\;x_{\bar{c}o}{\left(0\right)}^{T}\right]}^{T}$ and ${\left[x_{co}{\left(0\right)}^{T}\;\;0_{1\times q}\right]}^{T}$, respectively. The natural response component ${\tilde{y}}_{co}$ can be generated by the following minimal state-space representation of G(z), without the effect of its input u:

(79) $$\begin{array}{c} \underset{x_{co}\left(k\right)}{\underbrace{\left[\begin{array}{c} x_{co,1}\left(k\right)\\ x_{co,2}\left(k\right)\\ x_{co,3}\left(k\right)\\ \vdots \\ x_{co,p}\left(k\right) \end{array}\right]}}=\underset{A_{co}}{\underbrace{\left(\begin{array}{ccccc} b_{1} & b_{2} & b_{3} & \cdots & b_{p}\\ 1 & 0 & 0 & \cdots & 0\\ 0 & 1 & 0 & \ddots & \vdots \\ \vdots & \ddots & \ddots & \ddots & 0\\ 0 & \cdots & 0 & 1 & 0 \end{array}\right)}}\underset{x_{co}}{\underbrace{\left[\begin{array}{c} x_{co,1}\left(k-1\right)\\ x_{co,2}\left(k-1\right)\\ x_{co,3}\left(k-1\right)\\ \vdots \\ x_{co,p}\left(k-1\right) \end{array}\right],}} \end{array}$$

(80) $$\begin{array}{c} {\tilde{y}}_{co}\left(k\right)=\underset{a^{T}}{\underbrace{\left[a_{1}\;\;a_{2}\;\;\cdots \;\;a_{p}\right]}}x_{co}\left(k-1\right)+\underset{p^{T}}{\underbrace{\left[b_{1}\;\;b_{2}\;\;\cdots \;\;b_{p}\right]}}x_{co}\left(k-1\right). \end{array}$$

Now, we can state and prove the main result of this section:

**Theorem** **7.** *Suppose G satisfies Assumption 1 and $u_{1}^{\infty }$ is entropy balanced. Assume that $x_{o}\left(0\right)$ (the observable part of the initial state of G) is independent of the input $u_{1}^{\infty }$, $|h(x_{o}\left(0\right))|<\infty$ and that $\mathrm{tr}\{K_{x_{o}\left(0\right)}\}<\infty$. Then,*

(81) $$\begin{array}{c} \lim_{n\to \infty }\frac{1}{n}\left(h\left(y_{1}^{n}\right)-h\left(u_{1}^{n}\right)\right)=\sum_{i=1}^{m}\log\left|\rho_{i}\right|. \end{array}$$

**Proof.**  Both *G* and $u_{1}^{\infty }$ satisfy the conditions of Theorem 6. Thus, as in its statement, we write $G\left(z\right)=F\left(z\right)\tilde{G}\left(z\right)$, where $\tilde{G}\left(z\right)$ is stable and minimum phase and F(z) is a stable FIR transfer function with only the *m* non-minimum-phase zeros of G(z).

Defining $w_{n}^{1}≜{\tilde{G}}_{n}u_{n}^{1}$, we have

(82) $$\begin{array}{c} y_{n}^{1}=F_{n}{\tilde{G}}_{n}u_{n}^{1}+{\tilde{y}}_{n}^{1}=F_{n}w_{n}^{1}+{\tilde{y}}_{n}^{1}, \end{array}$$

(83) $$\begin{array}{c} h(w_{n}^{1})=h(u_{n}^{1}), \end{array}$$

and ${\tilde{y}}_{n}^{1}⫫w_{n}^{1}$. In addition, the fact that *G* is stable guarantees that the sample second moment of ${\tilde{y}}_{1}^{\infty }$ decays exponentially, which means that ${\tilde{y}}_{1}^{\infty }$ satisfies Assumption 2. Thus, the conditions of Lemma 6 are met considering $G_{n}=F_{n}$, where now $F_{n}=Q_{n}^{T}D_{n}R_{n}$ is the SVD for $F_{n}$, and $d_{n,1}\leq d_{n,2}\leq \cdots \leq d_{n,n}$. Consequently, the proof would be completed if we can show that $\lim_{n\to \infty }\frac{1}{n}h\left({\left[D_{n}\right]}_{m}^{1}R_{n}w_{n}^{1}+{\left[Q_{n}\right]}_{m}^{1}{\tilde{y}}_{n}^{1}\right)=0$. But all the involved variables have bounded variance, while $R_{n}$ is unitary, ${\left[Q_{n}\right]}_{m}^{1}$ has orthonormal rows and the entries of ${\left[D_{n}\right]}_{m}^{1}$ decay exponentially with *n*. This implies that $\lim_{n\to \infty }\frac{1}{n}h\left({\left[D_{n}\right]}_{m}^{1}R_{n}w_{n}^{1}+{\left[Q_{n}\right]}_{m}^{1}{\tilde{y}}_{n}^{1}\right)\leq 0$. Therefore, it is only left to prove that

(84) $$\begin{array}{c} \lim_{n\to \infty }\frac{1}{n}h\left({\left[D_{n}\right]}_{m}^{1}R_{n}w_{n}^{1}+{\left[Q_{n}\right]}_{m}^{1}{\tilde{y}}_{n}^{1}\right)\geq 0. \end{array}$$

Recalling (78), let us decompose ${\left[{\tilde{y}}_{co}\right]}_{n}^{1}$ so that

(85) $$\begin{array}{cc} {\tilde{y}}_{n}^{1} & =F_{n}{\tilde{P}}_{n}x_{co}\left(0\right)+P_{n}x_{co}\left(0\right)+{\left[{\tilde{y}}_{\bar{c}o}\right]}_{n}^{1}, \end{array}$$

where ${\tilde{P}}_{n},P_{n}\in R^{n\times (p+q)}$, the sequences $F_{n}{\tilde{P}}_{n}x_{co}\left(0\right)$ and $P_{n}x_{co}\left(0\right)$, respectively, are the natural responses of $\tilde{G}$ and *F* to the controllable and observable initial state $x_{co}$, and ${\left[{\tilde{y}}_{\bar{c}o}\right]}_{n}^{1}$ is the natural response of *G* to the non-controllable and observable initial state $x_{\bar{c}o}\left(0\right)$. Then,

(86) $$\begin{array}{cc} h({\left[D_{n}\right]}_{m}^{1}R_{n}w_{n}^{1}+{\left[Q_{n}\right]}_{m}^{1}{\tilde{y}}_{n}^{1}) & \overset{\left(a\right)}{\geq }h\left({\left[Q_{n}\right]}_{m}^{1}{\tilde{y}}_{n}^{1}\right)\overset{\left(85\right)}{=}h\left({\left[Q_{n}\right]}_{m}^{1}\left(F_{n}{\tilde{P}}_{n}x_{co}\left(0\right)+P_{n}x_{co}\left(0\right)+{\left[{\tilde{y}}_{\bar{c}o}\right]}_{n}^{1}\right)\right), \end{array}$$

(87) $$\begin{array}{c} \overset{\left(b\right)}{=}h\left({\left[Q_{n}\right]}_{m}^{1}\left(F_{n}{\tilde{P}}_{n}x_{co}\left(0\right)+P_{n}x_{co}\left(0\right)\right)\;|\;x_{\bar{c}o}\left(0\right)\right)=h\left({\left[Q_{n}\right]}_{m}^{1}\left(F_{n}{\tilde{P}}_{n}+P_{n}\right)x_{co}\left(0\right)\;|\;x_{\bar{c}o}\left(0\right)\right), \end{array}$$

where (a) is from the entropy-power inequality [1] and (b) holds because conditioning does not increase entropy and ${\left[{\tilde{y}}_{\bar{c}o}\right]}_{n}^{1}$ is a deterministic function of $x_{\bar{c}o}\left(0\right)$. Let the SVD of ${\left[Q_{n}\right]}_{m}^{1}\left(F_{n}{\tilde{P}}_{n}+P_{n}\right)$ be

(88) $$\begin{array}{c} {\left[Q_{n}\right]}_{m}^{1}\left(F_{n}{\tilde{P}}_{n}+P_{n}\right)=S_{n}T_{n}H_{n},\;\;\;\;n=m,m+1,\ldots , \end{array}$$

where $S_{n}\in R^{m\times m}$ is unitary, $T_{n}=\mathrm{diag}\left\{t_{1},t_{2},\ldots ,t_{m}\right\}$ holds the singular values of ${\left[Q_{n}\right]}_{m}^{1}\left(F_{n}{\tilde{P}}_{n}+P_{n}\right)$ and $H_{n}\in R^{m\times p}$ has orthonormal rows. Substituting this SVD into (87) we obtain

(89) $$\begin{array}{c} h\left({\left[D_{n}\right]}_{m}^{1}R_{n}w_{n}^{1}+{\left[Q_{n}\right]}_{m}^{1}{\tilde{y}}_{n}^{1}\right)\geq h\left(S_{n}T_{n}H_{n}x_{co}\left(0\right)\;|\;x_{\bar{c}o}\left(0\right)\right)=\log\left(\det\left(T_{n}\right)\right)+h\left(H_{n}x_{co}\left(0\right)\;|\;x_{\bar{c}o}\left(0\right)\right). \end{array}$$

This last differential entropy is bounded because $|h(x_{o})|<\infty$ and $\mathrm{tr}\{K_{x_{o}}\}<\infty$, which implies (thanks to Proposition A1) that $|h(H_{n}x_{co},x_{\bar{c}o})|<\infty$, and by the chain rule of entropy,

(90) $$\begin{array}{c} h\left(H_{n}x_{co},x_{\bar{c}o}\right)=h\left(x_{\bar{c}o}\right)+h\left(H_{n}x_{co}\left(0\right)\;|\;x_{\bar{c}o}\left(0\right)\right), \end{array}$$

so $|h(H_{n}x_{co}\left(0\right)\;|\;x_{\bar{c}o}\left(0\right))|<\infty$ because $|h(x_{\bar{c}o}\left(0\right))|<\infty$ (again from Proposition A1). Thus, in view of (89) and (84), all that remains to prove is that

(91) $$\begin{array}{c} \lim_{n\to \infty }\sigma_{\min}\left({\left[Q_{n}\right]}_{m}^{1}\left(F_{n}{\tilde{P}}_{n}+P_{n}\right)\right)>0, \end{array}$$

For that purpose, notice that ${\left[Q_{n}\right]}_{m}^{1}\left(F_{n}{\tilde{P}}_{n}+P_{n}\right)={}^{1}{\left[D_{n}\right]}_{m}{\left[R_{n}\right]}_{m}^{1}{\tilde{P}}_{n}+{\left[Q_{n}\right]}_{m}^{1}P_{n}$. Therefore, from Lemma A4 (in Appendix C), it follows that (91) holds if

(92) $$\begin{array}{c} \lim_{n\to \infty }\sigma_{\min}\left({\left[Q_{n}\right]}_{m}^{1}P_{n}\right)>0, \end{array}$$

and

(93) $$\begin{array}{c} \lim_{n\to \infty }\sigma_{\max}\left({}^{1}{\left[D_{n}\right]}_{m}{\left[R_{n}\right]}_{m}^{1}{\tilde{P}}_{n}\right)=0. \end{array}$$

To prove (93), recall that the entries in the diagonal matrix ${}^{1}{\left[D_{n}\right]}_{m}$ decay exponentially with *n*. On the other hand, the rows of ${\left[R_{n}\right]}_{m}^{1}$ are orthonormal. Finally, the fact that $\tilde{G}$ is stable implies that the p+q columns of ${\tilde{P}}_{n}$ have norms which are bounded for all *n*. These three observations readily yield that (93) holds.

To prove that (92) holds, write the rational transfer function of *G* (described by (80)) as

(94) $$\begin{array}{c} G\left(z\right)=\frac{1+a_{1}z^{-1}+\cdots +a_{p}z^{-p}}{1+b_{1}z^{-1}+\cdots +b_{p}z^{-p}}=\underset{F(z)}{\underbrace{\left(1+f_{1}z^{-1}+\cdots +f_{m}z^{-m}\right)}}\underset{\tilde{G}\left(z\right)}{\underbrace{\frac{1+{\tilde{a}}_{1}z^{-1}+\cdots +{\tilde{a}}_{\tilde{m}}z^{-\tilde{m}}}{1+b_{1}z^{-1}+\cdots +b_{p}z^{-p}}}}, \end{array}$$

where $\tilde{m}≜p-m$. The coefficients in the numerator of G(z) are related to those of F(z) and $\tilde{G}\left(z\right)$ by the convolution

(95) $$\begin{array}{c} a_{i}=\sum_{j=0}^{m}f_{j}{\tilde{a}}_{i-j},\;\;\;\;i=1,\ldots ,p, \end{array}$$

where ${\tilde{a}}_{0}=f_{0}=1$.

Denote the natural response of *F* (up to time *n*) to its initial state $x_{F}\left(0\right)$ (which is a linear function of $x_{co}\left(0\right)$) as

(96) $$\begin{array}{c} {\ddot{y}}_{n}^{1}≜P_{n}x_{co}\left(0\right). \end{array}$$

Let ${\tilde{w}}_{n}^{1}≜{\tilde{P}}_{n}x_{co}\left(0\right)$ be the natural response of $\tilde{G}$ to its initial state $x_{co}\left(0\right)$. Following the structure of (80), $\tilde{w}\left(k\right)$ can be written as

(97) $$\begin{array}{c} \tilde{w}\left(k\right)=\left[{\tilde{a}}_{1}\;\;\cdots \;\;{\tilde{a}}_{\tilde{m}}\;\;0\;\;\cdots \;\;0\right]x_{co}\left(k-1\right)+p^{T}x_{co}\left(k-1\right),\;\;\;\;k=1,2,\ldots , \end{array}$$

where $x_{co}$ satisfies (79). Considering the following minimal state-space representation of *F*

(98) $$\begin{array}{c} x_{F}\left(k\right)≜\left[\begin{array}{c} x_{F,1}\left(k\right)\\ x_{F,2}\left(k\right)\\ x_{F,3}\left(k\right)\\ \vdots \\ x_{F,m}\left(k\right) \end{array}\right]=\underset{A_{F}}{\underbrace{\left(\begin{array}{ccccc} 0 & 0 & \cdots & \cdots & 0\\ 1 & 0 & 0 & \cdots & 0\\ 0 & 1 & 0 & \ddots & \vdots \\ \vdots & \ddots & \ddots & \ddots & 0\\ 0 & \cdots & 0 & 1 & 0 \end{array}\right)}}\left[\begin{array}{c} x_{F,1}\left(k-1\right)\\ x_{F,2}\left(k-1\right)\\ x_{F,3}\left(k-1\right)\\ \vdots \\ x_{F,m}\left(k-1\right) \end{array}\right]+\left[\begin{array}{c} 1\\ 0\\ 0\\ \vdots \\ 0 \end{array}\right]w\left(k\right), \end{array}$$

(99) $$\begin{array}{c} {\tilde{y}}_{co}\left(k\right)=\underset{c_{F}^{T}}{\underbrace{\left[f_{1}\;\;f_{2}\;\;\cdots \;\;f_{m}\right]}}x_{F}\left(k-1\right)+\tilde{w}\left(k\right), \end{array}$$

it can be seen that the natural response of *F* to its own initial state $x_{F}\left(0\right)$ can be written as

(100) $$\begin{array}{c} \ddot{y}\left(k\right)={\tilde{y}}_{co}\left(k\right)-\tilde{w}\left(k\right)-“\mathrm{the}\;\mathrm{effect}\;\mathrm{of}\;f_{1},\ldots ,f_{k-1}”. \end{array}$$

But, from (80) and (95),

(101) $$\begin{array}{cc} {\tilde{y}}_{co}\left(k\right) & ={\tilde{a}}^{T}\mathrm{diag}{\left\{\left[f_{1}\;\;\cdots \;\;f_{m}\right]\right\}}_{\tilde{m}+1}x_{co}\left(k-1\right)+\underset{\tilde{w}\left(k\right)}{\underbrace{\left[{\tilde{a}}_{1}\;\;\cdots \;\;{\tilde{a}}_{\tilde{m}}\;\;0\;\;\cdots \;\;0\right]x_{co}\left(k-1\right)+p^{T}x_{co}\left(k-1\right)}}, \end{array}$$

where $\tilde{a}≜\left[1\;\;\tilde{a}\;\;\cdots \;\;{\tilde{a}}_{\tilde{m}}\right]$, and

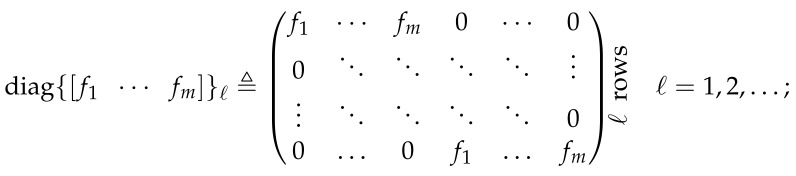
(102)
therefore,

(103) $$\begin{array}{c} \ddot{y}\left(1\right)={\tilde{a}}^{T}\mathrm{diag}{\left\{\left[f_{1}\;\;f_{2}\;\;\cdots \;\;f_{m}\right]\right\}}_{\tilde{m}+1}x_{co}\left(0\right), \end{array}$$

(104) $$\begin{array}{c} \ddot{y}\left(2\right)={\tilde{a}}^{T}\mathrm{diag}{\left\{\left[0\;\;\;f_{2}\;\;\cdots \;\;f_{m}\right]\right\}}_{\tilde{m}+1}A_{co}x_{co}\left(0\right), \end{array}$$

(105) $$\begin{array}{cc} & ={\tilde{a}}^{T}\mathrm{diag}{\left\{\left[f_{2}\;\;\cdots \;\;f_{m}\;\;\;0\right]\right\}}_{\tilde{m}+1}x_{co}\left(0\right) \end{array}$$

(106) $$\begin{array}{cc} & \vdots \end{array}$$

(107) $$\begin{array}{c} \ddot{y}\left(m\right)={\tilde{a}}^{T}\mathrm{diag}{\left\{\left[0\;\;\;\cdots \;\;\;0\;\;f_{m}\right]\right\}}_{\tilde{m}+1}A_{co}^{m-1}x_{co}, \end{array}$$

(108) $$\begin{array}{cc} & ={\tilde{a}}^{T}\mathrm{diag}{\left\{\left[f_{m}\;\;0\;\;\;\cdots \;\;\;0\right]\right\}}_{\tilde{m}+1}x_{co}\left(0\right), \end{array}$$

with $\ddot{y}\left(k\right)=0$ for k>m. Therefore,

(109) $$\begin{array}{c} {\ddot{y}}_{m}^{1}=Ex_{co}\left(0\right)=\underset{E}{\underbrace{\left[M\;|\;N\right]}}x_{co}\left(0\right), \end{array}$$

where $M\in R^{m\times (p-m)}$ and $N\in R^{m\times m}$ is a lower anti-triangular Toeplitz matrix with ${\tilde{a}}_{\tilde{m}}f_{m}$ along its main anti diagonal.

This implies that $P_{n}={\left[E^{T}\;|\;0_{p\times (n-m)}\right]}^{T}$ and

(110) $$\begin{array}{c} \sigma_{\min}\left(E\right)>0. \end{array}$$

Thus, resuming the reasoning before (94), we have that

(111) $$\begin{array}{c} {\left[Q_{n}\right]}_{m}^{1}P_{n}={}^{1}\;{\left[Q_{n}^{\left(p\right)}\right]}_{m}E. \end{array}$$

It then follows from (110) and Lemma 8 that

(112) $$\begin{array}{c} \lim_{n\to \infty }\sigma_{\min}\left({\left[Q_{n}\right]}_{m}^{1}P_{n}\right)=\lim_{n\to \infty }\sigma_{\min}\left({}^{1}\;{\left[Q_{n}\right]}_{m}E\right).>0. \end{array}$$

Hence, (91) is satisfied. Substituting (91) into (89) and the latter into (84) yields

(113) $$\begin{array}{c} \lim_{n\to \infty }\frac{1}{n}h\left({\left[D_{n}\right]}_{m}^{1}R_{n}w_{n}^{1}+{\left[Q_{n}\right]}_{m}^{1}{\bar{y}}_{n}^{1}\right)=0. \end{array}$$

The proof is completed by invoking Lemma (6).   □

Theorem 7 allows us to formalize the effect that the presence or absence of a random initial state has on the entropy gain using arguments similar to those utilized in Section 6.

## 8. Some Implications

The purpose of this section is to illustrate how the results obtained in the previous section can be applied to other problems. To do so, we present next some of the implications of these results on three different problems previously addressed in the literature, namely finding the rate-distortion function for non-stationary processes, an inequality in networked control theory, and the feedback capacity of Gaussian stationary channels. The common feature in these three problems is that, in all of them, non-minimum phase transfer functions play a role (either explicitly or implicitly).

### 8.1. Networked Control

**Figure 4:**
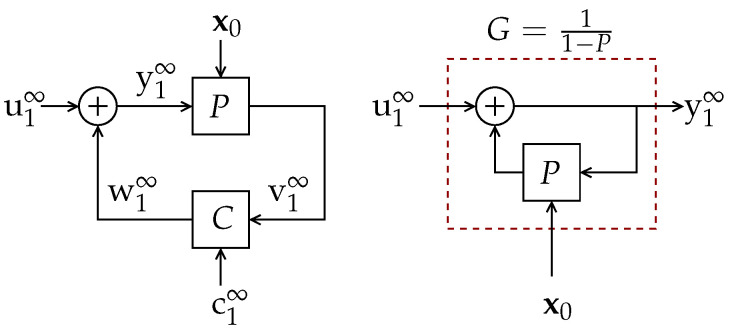
(**Left**): LTI system *P* within a noisy feedback loop. (**Right**): equivalent system when the feedback channel is noiseless and has unit gain.

The analysis developed in Reference [13] considers an LTI system *P* within a noisy feedback loop, as the one depicted in Figure 4. In this scheme, *C* represents a causal feedback channel which combines the output of *P* with an exogenous (noise) random process $c_{1}^{\infty }$ to generate its output. The process $c_{1}^{\infty }$ is assumed independent of the initial state of *P*, represented by the random vector $x_{0}$, which has finite differential entropy.

For this system, it is shown in Reference [13], Theorem 4.2, that

(114a) $$\begin{array}{c} \bar{h}\left(y_{1}^{\infty }\right)\geq \bar{h}\left(u_{1}^{\infty }\right)+\lim_{n\to \infty }\frac{1}{n}I\left(x_{0};y_{1}^{n}\right), \end{array}$$

where $I(x_{0};y_{1}^{n})$ is the mutual information (see Reference [1], Section 8.5) between $x_{0}$ and $y_{1}^{n}$, with equality if w is a deterministic function of v. Furthermore, it is shown in Reference [12], Lemma 3.2, that, if $|h(x_{0})|<\infty$ and the steady state variance of system *P* remains asymptotically bounded as k→∞, then

(114b) $$\begin{array}{c} \lim_{n\to \infty }\frac{1}{n}I\left(x_{0};y_{1}^{n}\right)\geq \sum_{p_{i}:\left|p_{i}\right|>1}\log\left|p_{i}\right|, \end{array}$$

where $\left\{p_{i}\right\}$ are the poles of *P*. Thus, for the (simplest) case in which w=v, the output $y_{1}^{\infty }$ is the result of filtering $u_{1}^{\infty }$ by a filter $G=\frac{1}{1-P}$ (as shown in Figure 4 right), and the resulting entropy rate of $y_{1}^{\infty }$ will exceed that of $u_{1}^{\infty }$ only if there is a random initial state with bounded differential entropy (see (114a)). Moreover, if w=v and G(z) is stable, (114) (as well as Reference [13], Lemma 4.3) implies that this entropy gain is lower bounded by the *right-hand side* (RHS) of (8), which is greater than zero if and only if *G* is NMP. However, both [12,13] do not provide conditions under which this lower bound is reached.

In Reference [14], Theorem 14, it is shown that, when there is perfect feedback (i.e., when v=w), as in Figure 4 right, with *P* being the concatenation of a stabilizing LTI controller and an LTI plant, and assuming $u_{1}^{\infty }$ is Gaussian i.i.d. and a Gaussian initial state, then

(115) $$\begin{array}{c} \bar{h}\left(y_{1}^{\infty }\right)-\bar{h}\left(u_{1}^{\infty }\right)=\sum_{p_{i}:\left|p_{i}\right|>1}\log\left|p_{i}\right|. \end{array}$$

Notice that this implies reaching equality in both (114a) and (114b).

By using the results obtained in Section 7 we show next that equality holds in (114b) provided the feedback channel satisfies the following assumption:

**Assumption** **3.** *The feedback channel in Figure 4 can be written as*

(116) $$\begin{array}{c} w=ABv+BF(c), \end{array}$$

*where:*

*1*. *A and B are stable rational transfer functions such that AB is biproper, ABP has the same unstable poles as P, and the feedback AB stabilizes the plant P.*
*2*. *F is any (possibly non-linear) operator such that $\tilde{c}≜F\left(c\right)$ has finite variance $\sigma_{\tilde{c}\left(n\right)}^{2}$ for finite n, $\lim_{n\to \infty }n^{-1}\log\left(\sigma_{\tilde{c}\left(n\right)}^{2}\right)=0$, and*
*3*. *$c_{1}^{\infty }⫫x_{0}$.*

We also extend Reference [14], Theorem 14, to situations including a feedback channel satisfying Assumption 3. For the perfect-feedback case, this extends the validity of (115) to a much larger class of distributions for $u_{1}^{\infty }$.

**Figure 5:**
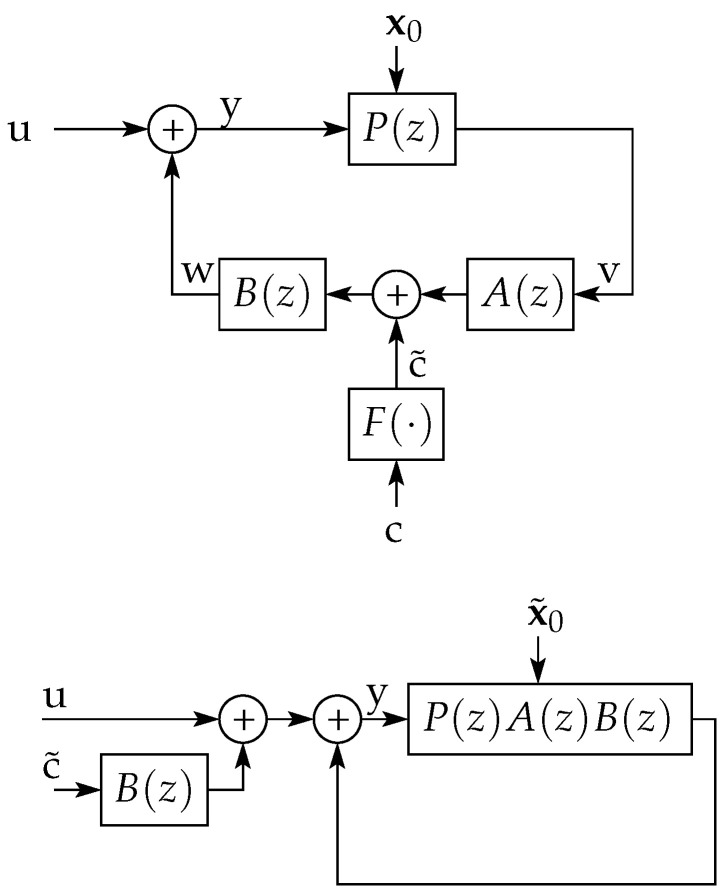
(**Top**): The class of feedback channels described by Assumption 3. (**Bottom**): an equivalent form.

An illustration of the class of feedback channels satisfying this assumption is depicted on top of Figure 5. Trivial examples of channels satisfying Assumption 5 are a Gaussian additive channel preceded and followed by linear operators [23]. Indeed, when *F* is an LTI system with a strictly causal transfer function, the feedback channel that satisfies Assumption 3 is widely known as a *noise shaper with input pre and post filter*, used in, e.g., References [24,25,26,27].

**Theorem** **8.** *In the networked control system of Figure 4, suppose that the feedback channel satisfies Assumption 3, that the plant P(z) has poles ${\left\{p_{i}\right\}}_{i}^{p}$, and that the input $u_{1}^{\infty }$ is entropy balanced. If the random initial states of AB and P, namely $s_{0}\in R^{q}$ and $x_{0}\in R^{p}$, respectively, are independent, have finite variance and $|h(x_{0})|<\infty$, then*

(117a) $$\begin{array}{c} \lim_{n\to \infty }\frac{1}{n}I\left(x_{0};y_{1}^{n}\right)=\sum_{\left|p_{i}\right|>1}\log\left|p_{i}\right|. \end{array}$$

*Moreover,*

(117b) $$\begin{array}{c} \lim_{n\to \infty }\frac{1}{n}\left(h\left(y_{1}^{n}\right)-h\left({\tilde{u}}_{1}^{n}\right)\right)=\sum_{\left|p_{i}\right|>1}\log\left|p_{i}\right|, \end{array}$$

*where $\tilde{u}≜u+B\tilde{c}$ (see Figure 5 bottom).*

**Proof.**  Let P(z)=N(z)/G(z) and T(z)≜A(z)B(z)=Γ(z)/Θ(z). We will first show that the output $y_{n}^{1}$ can be written as

(118) $$\begin{array}{c} y_{n}^{1}=G_{n}{\tilde{G}}_{n}{\tilde{u}}_{n}^{1}+G_{n}{\tilde{P}}_{n}{\left[x_{0}^{T}\;\;s_{0}^{T}\right]}^{T}+P_{n}x_{0}, \end{array}$$

where $\tilde{G}$ is the stable LTI system with biproper and MP transfer function

(119) $$\begin{array}{c} \tilde{G}\left(z\right)≜\frac{\Theta (z)}{\Theta (z)G(z)+N(z)\Gamma (z)}, \end{array}$$

with $s_{0}\in R^{q}$, $x_{0}\in R^{p}$ and ${\left[x_{0}^{T}\;\;s_{0}^{T}\right]}^{T}$ being the random initial states of *T*, *G*, and $\tilde{G}$, respectively, and

(120) $$\begin{array}{c} \tilde{u}≜u+B\tilde{c} \end{array}$$

(see Figure 5 bottom). The matrices ${\tilde{P}}_{n}\in R^{n\times p}$ and $P_{n}\in R^{n\times (p+q)}$. From Figure 5, it is clear that the transfer function from $\tilde{u}$ to y is $G\left(z\right)\frac{\Theta (z)}{\Theta (z)G(z)+N(z)\Gamma (z)}$, validating the first term on the RHS of (118). In addition, it is evident that the initial state of $\tilde{G}$ is a linear combination of $x_{0}$ and $s_{0}$, justifying the term ${\tilde{P}}_{n}{\left[x_{0}^{T}\;\;s_{0}^{T}\right]}^{T}$ as the natural response of $\tilde{G}$. Thus, it is only left to prove that the initial state of *G* is $x_{0}$. For that purpose, let $G\left(z\right)=1-\sum_{i=1}^{p}g_{i}z^{-i}$ and $N\left(z\right)=\sum_{i=1}^{p}n_{i}z^{-i}$. Define the following variables:

(121) $$\begin{array}{c} o≜\frac{1}{G}y,\;\;\;\;w≜No. \end{array}$$

Then, the recursion corresponding to P(z) is

(122) $$\begin{array}{c} o_{k}=\sum_{i=1}^{p}g_{i}o_{k-i}+y_{k},\;\;\;\;k\geq 1, \end{array}$$

(123) $$\begin{array}{c} w_{k}=\sum_{i=1}^{p}n_{i}o_{k-i},\;\;\;\;k\geq 1. \end{array}$$

This reveals that the initial state of P(z) corresponds to

(124) $$\begin{array}{c} x_{0}=\left[o_{1-p}\;\;o_{2-p}\;\;\cdots \;\;o_{0}\right]. \end{array}$$

But, from (121), o is also the output of $\tilde{G}$ to the input $\tilde{u}$, and

(125) $$\begin{array}{cc} y_{k} & =o_{k}-\sum_{i=1}^{p}f_{i}o_{k-i},\;\;\;\;k\geq 1, \end{array}$$

which means that the initial state of *G* is $x_{0}$.

Now, using (118), we have that

(126) $$\begin{array}{c} I(x_{0};y_{n}^{1})=h\left(y_{n}^{1}\right)-h\left(y_{n}^{1}|x_{0}\right), \end{array}$$

(127) $$\begin{array}{cc} & =h\left(y_{n}^{1}\right)-h\left(F_{n}\left[{\tilde{G}}_{n}{\tilde{u}}_{n}^{1}+{\tilde{P}}_{n}s_{0}\right]\right), \end{array}$$

(128) $$\begin{array}{cc} & =h\left(F_{n}{\tilde{u}}_{n}^{1}+P_{n}x_{0}\right)-h\left({\tilde{u}}_{n}^{1}\right), \end{array}$$

where the first equality is because $s_{0}⫫x_{0}$ and ${\bar{u}}_{n}^{1}≜{\tilde{G}}_{n}{\tilde{u}}_{n}^{1}+{\tilde{P}}_{n}s_{0}$. The last equality holds since the first sample of the unit-impulse response of *G* is 1. Since $u_{1}^{\infty }$ is entropy balanced, $\tilde{G}\left(z\right)$ is biproper, stable, and MP, and both ${\tilde{c}}_{1}^{\infty }$ and ${\tilde{P}}_{n}s_{0}$ have finite variance, it follows from Lemmas 3 and 4 that ${\bar{u}}_{1}^{\infty }$ is entropy balanced, as well. Thus, the proof of the first claim is completed by direct application of Theorem 7.

For the second claim,

(129) $$\begin{array}{c} h\left(y_{n}^{1}\right)-h\left({\tilde{u}}_{n}^{1}\right)\overset{\left(a\right)}{=}h\left(y_{n}^{1}\right)-h\left({\tilde{G}}_{n}{\tilde{u}}_{n}^{1}\right)=h\left(y_{n}^{1}\right)-h\left({\bar{u}}_{n}^{1}\right)+\left(h\left({\tilde{G}}_{n}{\tilde{u}}_{n}^{1}\right)-h\left({\tilde{u}}_{n}^{1}\right)\right), \end{array}$$

where (a) holds because the first sample of the unit-impulse response of $\tilde{G}$ is ${\tilde{g}}_{0}=\lim_{z\to \infty }\tilde{G}\left(z\right)=1$. Then,

(130) $$\begin{array}{c} \lim_{n\to \infty }\frac{1}{n}\left(h\left(y_{n}^{1}\right)-h\left({\tilde{u}}_{n}^{1}\right)\right)=\lim_{n\to \infty }\frac{1}{n}\left(h\left(y_{n}^{1}\right)-h\left({\bar{u}}_{n}^{1}\right)\right)+\lim_{n\to \infty }\frac{1}{n}\left(h\left({\tilde{G}}_{n}{\tilde{u}}_{n}^{1}\right)-h\left({\tilde{u}}_{n}^{1}\right)\right), \end{array}$$

(131) $$\begin{array}{cc} & \overset{\left(a\right)}{=}\lim_{n\to \infty }\frac{1}{n}\left(h\left(y_{n}^{1}\right)-h\left({\bar{u}}_{n}^{1}\right)\right), \end{array}$$

(132) $$\begin{array}{cc} & \overset{\left(b\right)}{=}\sum_{\left|p_{i}\right|>1}\log\left|p_{i}\right|, \end{array}$$

where (a) holds because $\tilde{G}\tilde{u}$ is entropy balanced (from Lemma 4), and ${\tilde{P}}_{n}s_{0}$ has finite variance, allowing us to apply Proposition A3. In turn, (b) follows from (128) and (117a). This completes the proof.   □

**Remark** **2.** *If A(z) had poles outside the unit circle, then Theorem 8 can still be applied by associating those poles to P.*

**Remark** **3.** *Under the conditions of Theorem 8, one has that if either $\bar{h}\left(u_{1}^{\infty }\right)$ or $\bar{h}\left({\tilde{c}}_{1}^{\infty }\right)$ exists, then the other entropy rate exists too. In that case, if c⫫u and defining $\bar{c}≜B\tilde{c}$, (117) yields*

(133) $$\begin{array}{c} \bar{h}\left(y_{1}^{\infty }\right)-\bar{h}\left(u_{1}^{\infty }\right)-\bar{h}\left({\bar{c}}_{1}^{\infty }\right)=\lim_{n\to \infty }\frac{1}{n}I\left(x_{0};y_{1}^{n}\right)=\sum_{\left|p_{i}\right|>1}\log\left|p_{i}\right|, \end{array}$$

*revealing that the gap in (114a) is exactly $\bar{h}\left({\bar{c}}_{1}^{\infty }\right)$. In addition, in the perfect-feedback scenario, Theorem 8 extends the validity of (115) from the Gaussian i.i.d. *u* and Gaussian $x_{0}$ considered in Reference [14], Theorem 14, to an entropy-balanced *u* and an $x_{0}$ with finite variance and finite differential entropy.*

### 8.2. Rate Distortion Function for Non-Stationary Processes

In this section, we obtain a simpler proof of a result by Gray, Hashimoto and Arimoto [15,16,17], which compares the rate distortion function (RDF) of a non-stationary auto-regressive Gaussian process $x_{1}^{\infty }$ (of a certain class to be defined shortly) to that of a corresponding stationary version, under MSE distortion. Our proof is based upon the ideas developed in the previous sections, and extends the class of non-stationary sources for which the results in References [15,16,17] are valid.

To be more precise, let ${\left\{a_{i}\right\}}_{i=1}^{\infty }$ and ${\left\{{\tilde{a}}_{i}\right\}}_{i=1}^{\infty }$ be, respectively, the impulse responses of two linear time-invariant filters *A* and $\tilde{A}$ with rational transfer functions

(134) $$\begin{array}{cc} A(z) & =\frac{z^{M}}{\prod_{i=1}^{M}\left(z-p_{i}\right)}, \end{array}$$

(135) $$\begin{array}{cc} \tilde{A}\left(z\right) & =\frac{z^{M}}{\prod_{i=1}^{M}\left|p_{i}^{*}\right|\left(z-1/p_{i}^{*}\right)}, \end{array}$$

where $\left|p_{i}\right|>1$, ∀i=1,…,M. From these definitions, it is clear that A(z) is unstable, $\tilde{A}\left(z\right)$ is stable, and

(136) $$\begin{array}{c} |A\left(e^{j\omega }\right)\left|=\right|\tilde{A}\left(e^{j\omega }\right)|,\;\;\;\;\;\forall \omega \in \left[-\pi ,\pi \right]. \end{array}$$

Notice also that $\lim_{\left|z\right|\to \infty }A\left(z\right)=1$ and $\lim_{\left|z\right|\to \infty }\tilde{A}\left(z\right)=1/\prod_{i=1}^{M}\left|p_{i}\right|$; thus,

(137) $$\begin{array}{ccc} a_{0}=1, & \; & {\tilde{a}}_{0}=\prod_{i=1}^{M}{\left|p_{i}\right|}^{-1}. \end{array}$$

**Figure 6:**
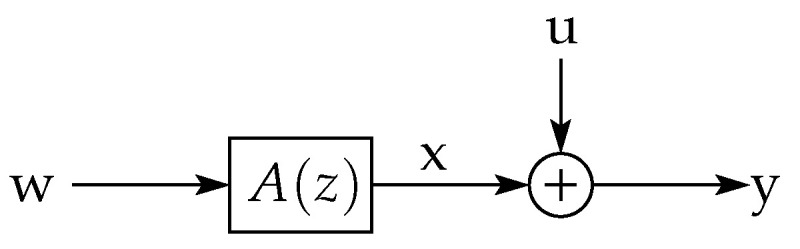
Block diagram representation of how the non-stationary source $x_{1}^{\infty }$ is built and then reconstructed as y=x+u.

Consider the non-stationary random sequence (source) $x_{1}^{\infty }$ and the asymptotically stationary source ${\tilde{x}}_{1}^{\infty }$ generated by passing a stationary Gaussian process $w_{1}^{\infty }$ through A(z) and $\tilde{A}\left(z\right)$, respectively, which can be written as

(138) $$\begin{array}{c} x_{n}^{1}=A_{n}w_{1}^{n},\;\;\;\;n=1,\ldots , \end{array}$$

(139) $$\begin{array}{c} {\tilde{x}}_{n}^{1}={\tilde{A}}_{n}w_{1}^{n},\;\;\;\;n=1,\ldots . \end{array}$$

(A block-diagram associated with the construction of x is presented in Figure 6.)

Define the rate-distortion functions for these two sources as

(140) $$\begin{array}{ccc} R_{x}\left(D\right)≜\lim_{n\to \infty }R_{x,n}\left(D\right),\; & R_{x,n}\left(D\right) & ≜\min\frac{1}{n}I\left(x_{1}^{n};x_{1}^{n}+u_{1}^{n}\right), \end{array}$$

(141) $$\begin{array}{ccc} R_{\tilde{x}}\left(D\right)≜\lim_{n\to \infty }R_{\tilde{x},n}\left(D\right),\; & R_{\tilde{x},n}\left(D\right) & ≜\min\frac{1}{n}I\left({\tilde{x}}_{1}^{n};{\tilde{x}}_{1}^{n}+{\tilde{u}}_{1}^{n}\right), \end{array}$$

where, for each *n*, the minima are taken over all the conditional probability density functions $f_{u_{1}^{n}|x_{1}^{n}}$ and $f_{{\tilde{u}}_{1}^{n}|{\tilde{x}}_{1}^{n}}$ yielding $E[∥u_{n}^{1}{∥}^{2}]/n\leq D$ and $E[∥{\tilde{u}}_{n}^{1}{∥}^{2}]/n\leq D$, respectively.

The above rate-distortion functions have been characterized in References [15,16,17] for the case in which $w_{1}^{\infty }$ is an i.i.d. Gaussian process. In particular, it is explicitly stated in References [16,17] that, for that case,

(142) $$\begin{array}{c} R_{x}\left(D\right)-R_{\tilde{x}}\left(D\right)=\frac{1}{2\pi }\;\;\int_{-\pi }^{\pi }\log|A^{-1}\left(e^{j\omega }\right)|d\omega =\sum_{i=1}^{M}\log\left|p_{i}\right|. \end{array}$$

We will next provide an alternative and simpler proof of this result, and extend its validity for general (not-necessarily stationary) Gaussian $w_{1}^{\infty }$, using the entropy gain properties of non-minimum phase filters established in Section 6. Indeed, the approach in References [15,16,17] is based upon asymptotically-equivalent Toeplitz matrices in terms of the signals’ covariance matrices. This restricts $w_{1}^{\infty }$ to be Gaussian and i.i.d. and A(z) to be an all-pole unstable transfer function, and then, the only non-stationarity allowed is that arising from unstable poles. For instance, a cyclo-stationary innovation followed by an unstable filter A(z) would yield a source which cannot be treated using Gray and Hashimoto’s approach. By contrast, the reasoning behind our proof lets $w_{1}^{\infty }$ be any entropy-balanced Gaussian process with bounded differential entropy rate, and then let the source be Aw, with A(z) having unstable poles (and possibly zeros and stable poles, as well).

The statement is as follows:

**Theorem** **9.** *Let $w_{1}^{\infty }$ be any Gaussian entropy-balanced process with bounded differential entropy rate, and let $x_{1}^{\infty }$ and ${\tilde{x}}_{1}^{\infty }$ be as defined in (138) and (139), respectively. Then, (142) holds.*

**Figure 7:**
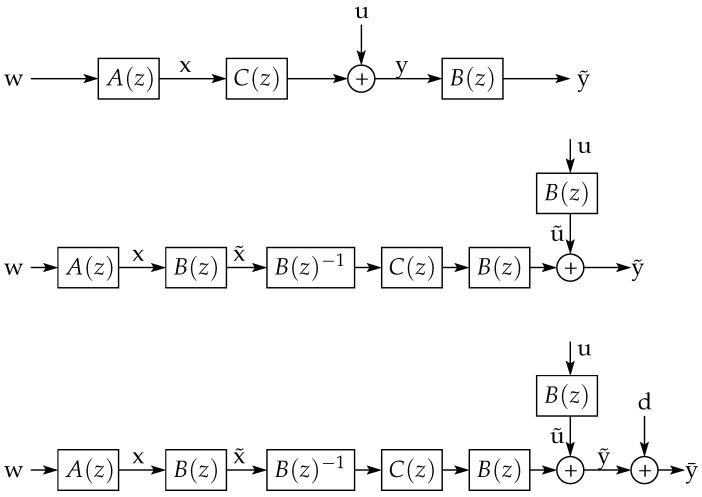
Block-diagram representation of the changes of variables in the proof of Theorem 9.

Thanks to the ideas developed in the previous sections, it is possible to give an intuitive outline of the proof of this theorem (given in Appendix B, page 40) by using a sequence of block diagrams. More precisely, consider the diagrams shown in Figure 7.

In the top diagram in this figure, suppose that y=Cx+u realizes the RDF for the non-stationary source x. The sequence u is independent of x, and the linear filter C(z) is such that the error (y−x)⫫y (a necessary condition for minimum MSE optimality). The filter B(z) is the Blaschke product of A(z) (see (A83) in Appendix B) (a stable, NMP filter with unit frequency response magnitude such that $\tilde{x}=Bx$).

If one moves the filter B(z) towards the source, then the middle diagram in Figure 7 is obtained. By doing this, the stationary source $\tilde{x}$ appears with an additive error signal $\tilde{u}$ that has the same asymptotic variance as u, reconstructed as $\tilde{y}=C\tilde{x}+\tilde{u}$. From the invertibility of B(z), it also follows that the mutual information rate between $\tilde{x}$ and $\tilde{y}$ equals that between x and y. Thus, the channel $\tilde{y}=C\tilde{x}+\tilde{u}$ has the same rate and distortion as the channel y=Cx+u.

However, if one now adds a short disturbance d to the error signal $\tilde{u}$ (as depicted in the bottom diagram of Figure 7), then the resulting additive error term $\bar{u}=\tilde{u}+d$ will be independent of $\tilde{x}$ and will have the same asymptotic variance as $\tilde{u}$. Nonetheless, the differential entropy rate of $\bar{u}$ will exceed that of $\tilde{u}$ by the RHS of (142). This will make the mutual information rate between $\tilde{x}$ and $\bar{y}$ to be less than that between $\tilde{x}$ and $\tilde{y}$ by the same amount. Hence, $R_{\tilde{x}}\left(D\right)$ is at most $R_{x}\left(D\right)-\sum_{i=1}^{m}\log\left|p_{i}\right|$. A similar reasoning can be followed to prove that $R_{x}\left(D\right)-R_{\tilde{x}}\left(D\right)\leq \sum_{i=1}^{m}\log\left|p_{i}\right|$.

### 8.3. The Feedback Channel Capacity of (Non-White) Gaussian Channels

Consider a non-white additive Gaussian channel of the form

(143) $$\begin{array}{c} y_{k}=x_{k}+z_{k}, \end{array}$$

where the input x is subject to the power constraint

(144) $$\begin{array}{c} \lim_{n\to \infty }\frac{1}{n}E[∥x_{n}^{1}{∥}^{2}]\leq P, \end{array}$$

and $z_{1}^{\infty }$ is a stationary Gaussian process.

The feedback information capacity of this channel is realized by a Gaussian input x, and is given by

(145) $$\begin{array}{c} C_{\mathrm{FB}}=\lim_{n\to \infty }\max_{K_{x_{n}^{1}}:\frac{1}{n}\mathrm{tr}\left\{K_{x_{n}^{1}}\right\}\leq P}I\left(x_{n}^{1};y_{n}^{1}\right), \end{array}$$

where $K_{x_{n}^{1}}$ is the covariance matrix of $x_{n}^{1}$, and, for every k∈N, the input $x_{k}$ is allowed to depend upon the channel outputs $y_{1}^{k-1}$ (since there exists a causal, noise-less feedback channel with one-step delay).

**Figure 8:**
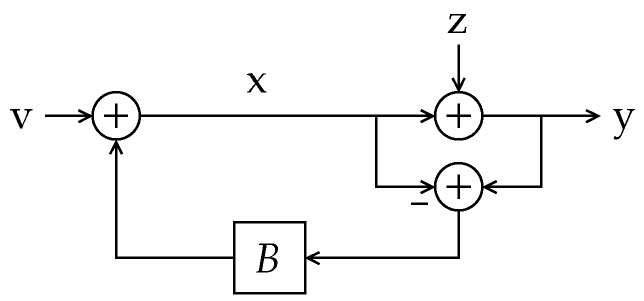
Block diagram representation a non-white Gaussian channel y=x+z and the coding scheme considered in Reference [9].

In Reference [9], it was shown that if z is an auto-regressive moving-average process of *M*-th order, then $C_{\mathrm{FB}}$ can be achieved by the scheme shown in Figure 8. In this system, *B* is a strictly causal and stable finite-order filter and $v_{1}^{\infty }$ is Gaussian with $v_{k}=0$ for all k>M and such that $v_{n}^{1}$ is Gaussian with a positive-definite covariance matrix $K_{v_{M}^{1}}$.

Here, we use the ideas developed in Section 6 to show that **the information rate achieved by the capacity-achieving scheme proposed in Reference [9] drops to zero if there exists any additive disturbance of length at least *M* and finite differential entropy affecting the output, no matter how small**.

To see this, notice that, in this case, and for all n>M,

(146) $$\begin{array}{c} I\left(x_{1}^{n};y_{1}^{n}\right)=I\left(v_{1}^{M};y_{1}^{n}\right)=h\left(y_{n}^{1}\right)-h\left(y_{n}^{1}|v_{n}^{1}\right), \end{array}$$

(147) $$\begin{array}{cc} & =h\left(y_{n}^{1}\right)-h\left(\left(I_{n}+B_{n}\right)z_{n}^{1}+v_{n}^{1}|v_{M}^{1}\right), \end{array}$$

(148) $$\begin{array}{cc} & =h\left(y_{n}^{1}\right)-h\left(\left(I_{n}+B_{n}\right)z_{n}^{1}|v_{M}^{1}\right), \end{array}$$

(149) $$\begin{array}{cc} & =h\left(y_{n}^{1}\right)-h\left(\left(I_{n}+B_{n}\right)z_{n}^{1}\right)=h\left(y_{n}^{1}\right)-h\left(z_{n}^{1}\right), \end{array}$$

(150) $$\begin{array}{cc} & =h\left(\left(I_{n}+B_{n}\right)z_{n}^{1}+v_{n}^{1}\right)-h\left(z_{n}^{1}\right), \end{array}$$

since $\det(I_{n}+B_{n})=1$. From Theorem 4, this gap between differential entries is precisely the entropy gain introduced by $I_{n}+B_{n}$ to an input $z_{n}^{1}$ when the output is affected by the disturbance $v_{M}^{1}$. Thus, from Theorem 4, the capacity of this scheme will correspond to $\frac{1}{2\pi }\;\;\int_{-\pi }^{\pi }\log\left|1+B(e^{j\omega })\right|d\omega =\sum_{\left|\rho_{i}\right|>1}\log\left|\rho_{i}\right|$, where ${\left\{\rho_{i}\right\}}_{i=1}^{M}$ are the zeros of 1+B(z), which is precisely the result stated in Reference [9], Theorem 4.1.

However, if the output is now affected by an additive disturbance $d_{1}^{\infty }$ not passing through B(z) such that $d_{k}=0$, ∀k>M and $|h(d_{M}^{1})|<\infty$, with $d_{1}^{\infty }⫫\left(v_{1}^{M},z_{1}^{\infty }\right)$, then we will have

(151) $$\begin{array}{c} y_{n}^{1}=v_{n}^{1}+\left(I_{n}+B_{n}\right)z_{n}^{1}+d_{n}^{1}. \end{array}$$

In this case,

(152) $$\begin{array}{c} I(x_{1}^{n};y_{1}^{n})=I\left(v_{1}^{M};y_{1}^{n}\right)=h\left(y_{n}^{1}\right)-h\left(y_{n}^{1}|v_{n}^{1}\right), \end{array}$$

(153) $$\begin{array}{cc} & =h\left(y_{n}^{1}\right)-h\left(\left(I_{n}+B_{n}\right)z_{n}^{1}+v_{n}^{1}+d_{n}^{1}|v_{M}^{1}\right), \end{array}$$

(154) $$\begin{array}{cc} & =h\left(y_{n}^{1}\right)-h\left(\left(I_{n}+B_{n}\right)z_{n}^{1}+d_{n}^{1}|v_{M}^{1}\right), \end{array}$$

(155) $$\begin{array}{cc} & =h\left(y_{n}^{1}\right)-h\left(\left(I_{n}+B_{n}\right)z_{n}^{1}+d_{n}^{1}\right). \end{array}$$

But $\lim_{n\to \infty }\frac{1}{n}\left(h\left(\left(I_{n}+B_{n}\right)z_{n}^{1}+v_{n}^{1}+d_{n}^{1}\right)-h\left(\left(I_{n}+B_{n}\right)z_{n}^{1}+d_{n}^{1}\right)\right)=0,$ which follows directly from applying Theorem 4 to each of the differential entries. Notice that this result holds irrespective of how small the power of the disturbance may be.

Thus, the capacity-achieving scheme proposed in Reference [9] (and further studied in Reference [28]), although of groundbreaking theoretical importance, would yield zero rate in any practical situation, since in every physically implemented scheme, signals are unavoidably affected by some amount of noise.

## 9. Conclusions

We have provided an intuitive explanation and a rigorous characterization of the entropy gain of a *linear time-invariant* (LTI) system, defined as the difference between the differential entropy rates of its output and input random signals. The continuous-time version of this problem, considered by Shannon in Theorem 14 of his 1948 landmark paper, involves an LTI system $G_{c}$ band limited to *B* [Hz]. For this scenario, we restricted our attention to systems such that the samples of its unit-impulse response, taken ${\left(2B\right)}^{-1}$ seconds apart, correspond to the unit-impulse response $g_{0},g_{1},\ldots$ of a causal and stable discrete-time system *G*. We show that the entropy gain in this case is $\log|g_{0}|$, which implies that, for this class of systems, Shannon’s Theorem 14 holds if and only if $G_{c}$ has a corresponding discrete-time *G* that is *minimum phase* (MP).

For the discrete-time case, we introduced a new notion referred to as effective differential entropy, which quantifies the amount of uncertainty in vector signals that are confined to subspaces of lower dimensionality than that of the signals themselves. (Note that this is not possible by the conventional notion of differential entropy, which simply diverges to minus infinity.) It turns out that the difference in effective differential entropy rate between an *n*-length input to an LTI discrete-time system with frequency response $G(e^{j\omega })$, and its full length output, as *n* tends to infinity, equals $\frac{1}{2\pi }\;\;\int_{-\pi }^{\pi }\left|G(e^{j\omega })\right|d\omega$.

When comparing input and output sequences of equal length, our analysis revealed that, in the absence of external random disturbances, the entropy gain of a discrete-time LTI system *G* with unit-impulse response $g_{0},g_{1},\ldots$ is simply $\log|g_{0}|$. An entropy gain greater than $\log|g_{0}|$ can be obtained only if a random signal is added to the output of *G* and if such output process has statistical properties that make it susceptible to the added random signal. In order to characterize the role of *G*, its input has been assumed to be *entropy balanced* (EB), a notion introduced herein. Crucially, the differential entropy rate of an EB process is not susceptible to random signals. EB processes constitute a large family that includes Gaussian processes with bounded, non-vanishing variance. We also show that (i) the sum of an EB process and any bounded variance process is EB, too, and (ii) passing an EB process by a stable MP filter yields an EB process. When the input is EB, we show that if *G* has NMP zeros $\rho_{1},\rho_{2},\ldots ,\rho_{m}$, then the largest possible entropy gain is $|g_{0}|+\sum_{i=1}^{m}\log\left|\rho_{i}\right|$, which equals $\frac{1}{2\pi }\;\;\int_{-\pi }^{\pi }\left|G(e^{j\omega })\right|d\omega$. This upper bound is achieved by adding a finite-length output disturbance with finite variance and bounded differential entropy if and only if its length is at least *m*, no matter how tiny its variance may be. The same entropy gain is also obtained if *G* has a random initial state with bounded differential entropy and finite variance.

We used these fundamental insights about when the entropy gain occurs in order to establish a new and more general proof of the quadratic rate-distortion function for non-stationary Gaussian sources. Moreover, we demonstrated that the information rate of the capacity-achieving scheme proposed in Reference [9] for the auto regressive Gaussian channel with feedback drops to zero in the presence of any additive disturbance in the channel input or output of sufficient (finite) length, no matter how small it may be. This has crucial implications in any physical setup, where noise is unavoidable.

## Data Availability

Not applicable.

## Appendix A. Proof of Theorem 3

The total length of the output *ℓ*, will grow with the length *n* of the input, if *G* is FIR, and will be infinite, if *G* is IIR. Letting η+1 be the length of the impulse response of *G* in the FIR case, we define the *output-length function*

(A1) $$\begin{array}{c} \ell \left(n\right)≜\mathrm{length}\;\mathrm{of}\;y\;\mathrm{when}\;\mathrm{input}\;\mathrm{is}\;u_{n}^{1}=\left\{\begin{array}{cc} n+\eta & ,\;\mathrm{if}\;G\;\mathrm{is}\;\mathrm{FIR},\\ \infty & ,\;\mathrm{if}\;G\;\mathrm{is}\;\mathrm{IIR}. \end{array}\right. \end{array}$$

It is also convenient to define the sequence of matrices ${\left\{{\overset{˘}{G}}_{n}\right\}}_{n=1}^{\infty }$, where ${\overset{˘}{G}}_{n}\in R^{\ell (n)\times n}$ is Toeplitz with ${\left[{\overset{˘}{G}}_{n}\right]}_{i,j}=0,\forall i<j$, ${\left[{\overset{˘}{G}}_{n}\right]}_{i,j}=g_{i-j},\forall i\geq j$. This allows one to write the *entire* output $y_{1}^{\ell }$ of a causal LTI filter *G* with impulse response ${\left\{g_{k}\right\}}_{k=0}^{\eta }$ to an input $u_{1}^{\infty }$ as

(A2) $$\begin{array}{c} y_{\ell (n)}^{1}\left(u_{n}^{1}\right)={\overset{˘}{G}}_{n}u_{n}^{1}. \end{array}$$

Let the SVD of ${\overset{˘}{G}}_{n}$ be ${\overset{˘}{G}}_{n}={\overset{˘}{Q}}_{n}^{T}{\overset{˘}{D}}_{n}{\overset{˘}{R}}_{n}$, where ${\overset{˘}{Q}}_{n}\in R^{n\times \ell (n)}$ has orthonormal rows, ${\overset{˘}{D}}_{n}\in R^{n\times n}$ is diagonal with positive elements, and ${\overset{˘}{R}}_{n}\in R^{n\times n}$ is unitary.

The effective differential entropy of $y_{1}^{\ell (n)}\left(u_{1}^{n}\right)$ exceeds the differential entropy of $u_{1}^{n}$ by

(A3) $$\begin{array}{c} \overset{˘}{h}\left(y_{\ell (n)}^{1}\left(u_{n}^{1}\right)\right)-h\left(u_{n}^{1}\right)=h\left({\overset{˘}{Q}}_{n}{\overset{˘}{G}}_{n}u_{n}^{1}\right)-h\left(u_{n}^{1}\right)=h\left({\overset{˘}{D}}_{n}{\overset{˘}{R}}_{n}u_{n}^{1}\right)-h\left(u_{n}^{1}\right)=\log\det\left({\overset{˘}{D}}_{n}\right). \end{array}$$

The determinant of $D_{n}$ can be related to that of ${\overset{˘}{G}}_{n}^{T}{\overset{˘}{G}}_{n}$ by noticing that

(A4) $$\begin{array}{c} {\overset{˘}{G}}_{n}^{T}{\overset{˘}{G}}_{n}={\left({\overset{˘}{Q}}_{n}^{T}{\overset{˘}{D}}_{n}{\overset{˘}{R}}_{n}\right)}^{T}\left({\overset{˘}{Q}}_{n}^{T}{\overset{˘}{D}}_{n}{\overset{˘}{R}}_{n}\right)={\overset{˘}{R}}_{n}^{T}{\overset{˘}{D}}_{n}{\overset{˘}{Q}}_{n}{\overset{˘}{Q}}_{n}^{T}{\overset{˘}{D}}_{n}{\overset{˘}{R}}_{n}={\overset{˘}{R}}_{n}^{T}{\overset{˘}{D}}_{n}^{2}{\overset{˘}{R}}_{n}. \end{array}$$

Since ${\overset{˘}{R}}_{n}$ is unitary, it follows that $\det{\overset{˘}{D}}_{n}^{2}=\det{\overset{˘}{G}}_{n}^{T}{\overset{˘}{G}}_{n}$, which from (A3) means that

(A5) $$\begin{array}{c} \overset{˘}{h}\left(y_{\ell (n)}^{1}\left(u_{n}^{1}\right)\right)-h\left(u_{n}^{1}\right)=\frac{1}{2}\log\left(\det\left({\overset{˘}{G}}_{n}^{T}{\overset{˘}{G}}_{n}\right)\right). \end{array}$$

The product $H_{n}≜{\overset{˘}{G}}_{n}^{T}{\overset{˘}{G}}_{n}$ is a symmetric Toeplitz matrix, with its first column, ${\left[h_{0}\;h_{1}\;\cdots \;h_{n-1}\right]}^{T}$, given by

$h_{i}=\sum_{k=0}^{n}g_{k}g_{k-i}$. Thus, the sequence ${\left\{h_{i}\right\}}_{i=0}^{n-1}$ corresponds to the samples 0 to n−1 of those resulting from the complete convolution $g*g^{-}$, even when the filter *G* is IIR, where $g^{-}$ denotes the time-reversed (possibly infinitely long) response *g*. Consequently, and since G(z) has no zeros on the unit circle, and *g* is absolutely summable, we can use the Grenander and Szegö’s theorem [29], and Reference [18], Theorem 4.2, to obtain that

(A6) $$\begin{array}{c} \lim_{n\to \infty }\log\left(\det{\left({\overset{˘}{G}}_{n}^{T}{\overset{˘}{G}}_{n}\right)}^{1/n}\right)=\frac{1}{2\pi }\;\;\int_{-\pi }^{\pi }\log{\left|G(e^{j\omega })\right|}^{2}d\omega . \end{array}$$

In order to finish the proof, we divide (A5) by *n*, take the limit as n→∞, and replace (A6) in the latter.

## Appendix B. Proofs of Results Stated in the Previous Sections

**Proof** **of Lemma 1.** Let $\sigma_{u(k)}^{2}$ be the variance of u(k). Thus, $h\left(u_{n}^{1}\right)=\frac{1}{2}\log({\left(2\pi e^{}\right)}^{n}\det(\mathrm{diag}$${\left\{\sigma_{u(k)}^{2}\right\}}_{k=1}^{n}))$. Let $y_{n}^{\nu +1}≜\Phi_{n}u_{n}^{1}$. Then, $K_{y_{n}^{\nu +1}}=\Phi_{n}\mathrm{diag}{\left\{\sigma_{u(k)}^{2}\right\}}_{k=1}^{n}\Phi_{n}^{T}$. As a consequence,

(A7) $$\begin{array}{c} h\left(y_{n}^{\nu +1}\right)=\frac{1}{2}\log\left({\left(2\pi e^{}\right)}^{\left[n-\nu \right]}\det\left(\Phi_{n}\mathrm{diag}{\left\{\sigma_{u(k)}^{2}\right\}}_{k=1}^{n}\Phi_{n}^{T}\right)\right). \end{array}$$

But from the Courant-Fischer theorem [30],

(A8) $$\begin{array}{c} \log\left(\det(\mathrm{diag}{\left\{\sigma_{u(k)}^{2}\right\}}_{k=1}^{n})\right)-\nu \log\left({\hat{\sigma }}^{2}\right)\leq \log\left(\det(\Phi_{n}\mathrm{diag}{\left\{\sigma_{u(k)}^{2}\right\}}_{k=1}^{n}\Phi_{n}^{T})\right)\leq \log\left(\det(\mathrm{diag}{\left\{\sigma_{u(k)}^{2}\right\}}_{k=1}^{n})\right)-\nu \log\left({\overset{ˇ}{\sigma }}^{2}\right); \end{array}$$

thus, $\lim_{n\to \infty }\frac{1}{n}\left(h\left(y_{n}^{\nu +1}\right)-h\left(u_{n}^{1}\right)\right)=0$, satisfying Condition ii) in Definition 2. Adding to this the fact that, in this case, $\sigma_{u(n)}^{2}\leq {\hat{\sigma }}^{2}<\infty$ for all *n*, Condition i) in Definition 2 is satisfied, as well, completing the proof.   □

**Proof** **of Lemma 2.** Let ${\left\{b_{i,\ell }\right\}}_{\ell =1}^{\infty }$ be the intervals (bins) in R where the sample u(i) has constant PDF. Define the discrete random process $c_{1}^{\infty }$, where c(i)=ℓ if and only if $u\left(i\right)\in b_{i,\ell }$. Let $y_{n}^{\nu +1}≜\Phi_{n}u_{n}^{1}$ where $\Phi_{n}\in R^{(n-\nu )\times n}$ has orthonormal rows. Then,

(A9) $$\begin{array}{c} h(y_{n}^{\nu +1})=h\left(y_{n}^{\nu +1}|c_{n}^{1}\right)+I\left(c_{n}^{1};y_{n}^{\nu +1}\right) \end{array}$$

(A10) $$\begin{array}{cc} & \leq h\left(y_{n}^{\nu +1}|c_{n}^{1}\right)+I\left(c_{n}^{1};u_{n}^{1}\right), \end{array}$$

where the inequality is due to the fact that $u_{n}^{1}$ and $y_{n}^{\nu +1}$ are deterministic functions of $u_{n}^{1}$; hence, $c_{n}^{1}⟷u_{n}^{1}⟷y_{n}^{\nu +1}$. Subtracting $h(u_{n}^{1})$ from (A9) we obtain

(A11) $$\begin{array}{c} h\left(y_{n}^{\nu +1}\right)-h\left(u_{n}^{1}\right)\leq h\left(y_{n}^{\nu +1}|c_{n}^{1}\right)+I\left(c_{n}^{1};u_{n}^{1}\right)-h\left(u_{n}^{1}\right) \end{array}$$

(A12) $$\begin{array}{cc} & =h\left(y_{n}^{\nu +1}|c_{n}^{1}\right)-h\left(u_{n}^{1}|c_{n}^{1}\right). \end{array}$$

Hence,

(A13) $$\begin{array}{c} \lim_{n\to \infty }\frac{1}{n}\left(h\left(y_{n}^{\nu +1}\right)-h\left(u_{n}^{1}\right)\right)\leq \lim_{n\to \infty }\frac{1}{n}\left(h\left(y_{n}^{\nu +1}|c_{n}^{1}\right)-h\left(u_{n}^{1}|c_{n}^{1}\right)\right)=0, \end{array}$$

where the last equality follows from Lemma A1 (in Appendix C) whose conditions are met because, given $c_{n}^{1}$, the sequence $u_{n}^{1}$ has independent entries each of them distributed uniformly over a possibly different interval with finite and positive measure. The opposite inequality is obtained by following the same steps as in the proof of Lemma A1, from (A124) onwards, which completes the proof.   □

**Proof** **of Lemma 3.** Let $y_{n}^{1}≜{\left[\Psi_{n}^{T}|\Phi_{n}^{T}\right]}^{T}w_{n}^{1}$, where ${\left[\Psi_{n}^{T}|\Phi_{n}^{T}\right]}^{T}\in R^{n\times n}$ is a unitary matrix and where $\Psi_{n}\in R^{\nu \times n}$ and $\Phi_{n}\in R^{(n-\nu )\times n}$ have orthonormal rows. Then,

(A14) $$\begin{array}{c} h(y_{n}^{\nu +1})=h\left(y_{n}^{1}\right)-h\left(y_{\nu }^{1}|y_{n}^{\nu +1}\right)=h\left(w_{n}^{1}\right)-h\left(y_{\nu }^{1}|y_{n}^{\nu +1}\right). \end{array}$$

We can lower bound $h(y_{\nu }^{1}|y_{n}^{\nu +1})$ as follows:

(A15) h(yν1|ynν+1)=(c)h(Ψnun1+Ψnvn1|Φnun1+Φnvn1)

(A16) $$\begin{array}{cc} & \overset{\left(a\right)}{\geq }h\left(\Psi_{n}u_{n}^{1}+\Psi_{n}v_{n}^{1}\;|\;\Phi_{n}u_{n}^{1}+\Phi_{n}v_{n}^{1}\;,\;v_{n}^{1}\right) \end{array}$$

(A17) =(c)h(Ψnun1|Φnun1+Φnvn1,vn1)

(A18) =(c)h(Ψnun1|Φnun1,vn1)

(A19) $$\begin{array}{cc} & \overset{\left(b\right)}{=}h\left(\Psi_{n}u_{n}^{1}\;|\;\Phi_{n}u_{n}^{1}\right) \end{array}$$

(A20) $$\begin{array}{cc} & \overset{\left(c\right)}{=}h\left(u_{n}^{1}\right)-h\left(\Phi_{n}u_{n}^{1}\right), \end{array}$$

where (a) holds because conditioning does not increase entropy, (b) is from the fact that $u_{n}^{1}⫫v_{n}^{1}$, and (c) follows from the chain rule of entropy. Substituting this result into (A14), dividing by *n*, taking the limit as n→∞, and recalling that $u_{1}^{\infty }$ is entropy balanced, we conclude that $\lim_{n\to \infty }\frac{1}{n}\left(h\left(\Phi_{n}w_{n}^{1}\right)-h\left(w_{n}^{1}\right)\right)\leq 0$. The opposite bound over $h(y_{\nu }^{1}|y_{n}^{\nu +1})$ can be obtained from

(A21) $$\begin{array}{c} h(y_{\nu }^{1}|y_{n}^{\nu +1})=h\left(\Psi_{n}u_{n}^{1}+\Psi_{n}v_{n}^{1}\;|\;\Phi_{n}u_{n}^{1}+\Phi_{n}v_{n}^{1}\right)\leq h\left(\Psi_{n}u_{n}^{1}+\Psi_{n}v_{n}^{1}\right)\leq h\left(\Psi_{n}{\left(w_{G}\right)}_{n}^{1}\right), \end{array}$$

where ${\left(w_{G}\right)}_{n}^{1}$ is a jointly Gaussian sequence with the same second-order moment as $w_{n}^{1}$. Therefore, $h\left(\Psi_{n}{\left(w_{G}\right)}_{n}^{1}\right)=\frac{1}{2}\log\left({\left(2\pi e^{}\right)}^{\nu }\det\left(\Psi_{n}K_{w_{n}^{1}}\Psi_{n}^{T}\right)\right)\leq \frac{\nu }{2}\log\left(2\pi e^{}\lambda_{\max}\left(K_{w_{n}^{1}}\right)\right)$. But $w_{n}^{1}$ satisfies the assumptions of Proposition A2; thus, $\lim_{n\to \infty }n^{-1}\log\left(\lambda_{\max}\left(K_{w_{n}^{1}}\right)\right)=0$. Therefore, $\lim_{n\to \infty }n^{-1}h\left(\Psi_{n}{\left(w_{G}\right)}_{n}^{1}\right)\leq 0$, which substituted in (A14) yields

$$\lim_{n\to \infty }-\frac{1}{n}h\left(y_{\nu }^{1}|y_{n}^{\nu +1}\right)=\lim_{n\to \infty }\frac{1}{n}\left(h\left(\Phi_{n}w_{n}^{1}\right)-h\left(w_{n}^{1}\right)\right)\geq 0.$$

Hence, $w_{1}^{\infty }$ satisfies Condition ii) of Definition 2. Since $w_{1}^{\infty }$ also satisfies Condition i) of Definition 2, it follows that $w_{1}^{\infty }$ is entropy balanced, completing the proof.   □

**Proof** **of Lemma 4.** Pick any ν∈N and let $y_{n}^{1}≜{\left[\Phi_{n}^{T}|\Psi_{n}^{T}\right]}^{T}w_{n}^{1}$ where ${\left[\Phi_{n}^{T}|\Psi_{n}^{T}\right]}^{T}\in R^{n\times n}$ is a unitary matrix and the matrices $\Psi_{n}\in R^{\nu \times n}$ and $\Phi_{n}\in R^{(n-\nu )\times n}$ have orthonormal rows. Since $w_{n}^{1}=G_{n}u_{n}^{1}$, we have that

(A22) $$\begin{array}{c} \Phi_{n}w_{n}^{1}=\Phi_{n}G_{n}u_{n}^{1}. \end{array}$$

Let $\Phi_{n}G_{n}=A_{n}\Sigma_{n}B_{n}$ be the SVD of $\Phi_{n}G_{n}$, where $A_{n}\in R^{\left(n-\nu )\times (n-\nu \right)}$ is an orthogonal matrix, $B_{n}\in R^{(n-\nu )\times n}$ has orthonormal rows and $\Sigma_{n}\in R^{\left(n-\nu )\times (n-\nu \right)}$ is a diagonal matrix with the singular values of $\Phi_{n}G_{n}$. Hence

(A23) $$\begin{array}{c} h\left(\Phi_{n}w_{n}^{1}\right)=h\left(\Phi_{n}G_{n}u_{n}^{1}\right)=h\left(A_{n}\Sigma_{n}B_{n}u_{n}^{1}\right)=\log\det\left(\Sigma_{n}\right)+h\left(B_{n}u_{n}^{1}\right). \end{array}$$

The singular values of $\Phi_{n}G_{n}$ are $\sigma_{i}\left(\Phi_{n}G_{n}\right)=\sqrt{\lambda_{i}\left(\Phi_{n}G_{n}G_{n}^{T}\Phi_{n}^{T}\right)}$, i=1,2,…,n−ν. Now, notice that

(A24) $$\begin{array}{c} \lambda_{i}\left({\left[\Phi_{n}^{T}|\Psi_{n}^{T}\right]}^{T}GG^{T}\left[\Phi_{n}^{T}|\Psi_{n}^{T}\right]\right)=\lambda_{i}\left(GG^{T}\right) \end{array}$$

and that

(A25) $$\begin{array}{c} {\left[\Phi_{n}^{T}|\Psi_{n}^{T}\right]}^{T}GG^{T}\left[\Phi_{n}^{T}|\Psi_{n}^{T}\right]=\left(\begin{array}{cc} \Phi_{n}GG^{T}\Phi_{n}^{T} & \Phi_{n}GG^{T}\Psi_{n}^{T}\\ \Psi_{n}GG^{T}\Phi_{n}^{T} & \Psi_{n}GG^{T}\Psi_{n}^{T} \end{array}\right). \end{array}$$

Thus, from (A24) and the Cauchy eigenvalue interlacing theorem [30],

(A26) $$\begin{array}{c} \lambda_{i}\left(G_{n}G_{n}^{T}\right)\leq \lambda_{i}\left(\Phi_{n}GG^{T}\Phi_{n}^{T}\right)\leq \lambda_{i+\nu }\left(G_{n}G_{n}^{T}\right),\;\;\;\;i=1,\ldots ,n-\nu . \end{array}$$

Hence,

(A27) $$-\frac{\nu }{2n}\log\left(\lambda_{\max}\left(G_{n}G_{n}^{T}\right)\right)\leq \frac{1}{n}\log\left(\det(\Sigma_{n})\right)-\frac{1}{n}\log\left|\det(G_{n})\right|\leq -\frac{\nu }{2n}\log\left(\lambda_{\min}\left(G_{n}G_{n}^{T}\right)\right).$$

Recalling that *G* is minimum phase (which guarantees that its singular values change at most polynomially with *n*, due to Lemma 7), we conclude that

(A28) $$\begin{array}{c} \lim_{n\to \infty }\frac{1}{n}\log\left(\det(\Sigma_{n})\right)=\frac{1}{n}\log\left|\det\left(G_{n}\right)\right|. \end{array}$$

Substituting back into (A23), we arrive to

(A29) $$\begin{array}{c} \lim_{n\to \infty }\frac{1}{n}h\left(\Phi_{n}w_{n}^{1}\right)\overset{\left(a\right)}{=}\lim_{n\to \infty }\frac{1}{n}\log\left|\det\left(G_{n}\right)\right|+\lim_{n\to \infty }\frac{1}{n}h\left(u_{1}^{n}\right)=\lim_{n\to \infty }\frac{1}{n}h\left(w_{1}^{n}\right), \end{array}$$

where (a) holds because $u_{1}^{\infty }$ is entropy balanced. This completes the proof.   □

**Proof** **of Lemma 5.** Let ${\left\{\Psi_{n}\right\}}_{n=1}^{\infty }$ be a sequence of matrices, each $\Psi_{n}\in R^{\kappa \times n}$ with orthonormal rows spanning a subspace of $R^{n}$ that contains the span of the columns of ${\left[\Phi \right]}_{n}^{1}$. For each n∈N, let ${\bar{\Psi }}_{n}\in R^{(n-\kappa )\times n}$ be such that $H_{n}≜{\left[\Psi_{n}^{T}\;|\;{\bar{\Psi }}_{n}^{T}\right]}^{T}$ is a unitary matrix. Then,

(A30) $$\begin{array}{c} h\left({\bar{\Psi }}_{n}y_{n}^{1}\right)=h\left({\bar{\Psi }}_{n}u_{n}^{1}\right). \end{array}$$

Thus,

(A31) $$\begin{array}{c} \lim_{n\to \infty }\frac{1}{n}\left(h\left(y_{n}^{1}\right)-h\left(u_{n}^{1}\right)\right)=\lim_{n\to \infty }\frac{1}{n}\left(\left(h\left(y_{n}^{1}\right)-h\left({\bar{\Psi }}_{n}y_{n}^{1}\right)\right)-\left(h\left(u_{n}^{1}\right)-h\left({\bar{\Psi }}_{n}u_{n}^{1}\right)\right)\right)=0, \end{array}$$

where the last equality holds because $u_{1}^{\infty }$ is entropy balanced and $y_{1}^{\infty }$ is entropy balanced (from Lemma 3). This completes the proof.   □

**Proof** **of Lemma 6.** Since $Q_{n}$ is unitary, we have that

(A32) $$\begin{array}{c} h\left(y_{n}^{1}\right)=h\left(Q_{n}y_{n}^{1}\right)=h\left(\overset{w_{n}^{1}}{\overbrace{\underset{v_{n}^{1}}{\underbrace{D_{n}R_{n}u_{n}^{1}}}+\underset{{\bar{z}}_{n}^{1}}{\underbrace{Q_{n}z_{n}^{1}}}}}\right)=h\left(w_{n}^{1}\right), \end{array}$$

where

(A33) $$\begin{array}{c} w_{n}^{1}≜Q_{n}y_{n}^{1}=v_{n}^{1}+{\bar{z}}_{n}^{1}, \end{array}$$

(A34) $$\begin{array}{c} v_{n}^{1}≜D_{n}R_{n}u_{n}^{1}, \end{array}$$

(A35) $$\begin{array}{c} {\bar{z}}_{n}^{1}≜Q_{n}z_{n}^{1}. \end{array}$$

Thus,

(A36) $$\begin{array}{c} h(y_{n}^{1})=h\left(w_{1}^{n}\right)\overset{\left(a\right)}{=}h\left(w_{1}^{m}\right)+h\left(w_{m+1}^{n}|w_{1}^{m}\right)=h\left({\left[D_{n}\right]}_{m}^{1}R_{n}u_{n}^{1}+{\left[Q_{n}\right]}_{m}^{1}z_{n}^{1}\right)+h\left(w_{m+1}^{n}|w_{1}^{m}\right), \end{array}$$

where (a) follows from the chain rule of differential entropy. It only remains to show that the limit of $\left(1/n\right)h\left(w_{m+1}^{n}|w_{1}^{m}\right)$ as n→∞ equals the entropy rate of $u_{1}^{\infty }$. We will do this by deriving a lower and an upper bounds which converge to the same expression as n→∞. A lower bound for $h(w_{m+1}^{n}|w_{1}^{m})$ can be obtained by noticing that

(A37) $$\begin{array}{c} h(w_{m+1}^{n}|w_{1}^{m})=h(v_{m+1}^{n}+\;{\bar{z}}_{m+1}^{n}|v_{1}^{m}+\;{\bar{z}}_{1}^{m}) \end{array}$$

(A38) $$\begin{array}{cc} \; & \overset{\left(a\right)}{\geq }h\left(v_{m+1}^{n}+\;{\bar{z}}_{m+1}^{n}|v_{1}^{m},\;{\bar{z}}_{1}^{n}\right) \end{array}$$

(A39) $$\begin{array}{cc} \; & \overset{\left(b\right)}{=}h\left(v_{m+1}^{n}|v_{1}^{m},\;{\bar{z}}_{1}^{n}\right) \end{array}$$

(A40) $$\begin{array}{cc} \; & \overset{\left(c\right)}{=}h\left(v_{m+1}^{n}|v_{1}^{m}\right) \end{array}$$

(A41) $$\begin{array}{cc} \; & \overset{\left(d\right)}{=}h\left(v_{1}^{n}\right)-h\left(v_{1}^{m}\right) \end{array}$$

(A42) $$\begin{array}{cc} \; & \overset{\left(e\right)}{=}h\left(u_{1}^{n}\right)-h\left(v_{1}^{m}\right), \end{array}$$

where (a) follows from the fact that conditioning on more information does not increase differential entropy, (b) is due to the fact that h(x+a)=h(x), for any constant *a*, (c) holds because ${\bar{z}}_{1}^{\infty }⫫v_{1}^{\infty }$, (d) is a direct application of the chain rule of differential entropy, and (e) stems from (A34) and the fact that $\det(D_{n}R_{n})=1$. On the other hand,

(A43) $$\begin{array}{c} h\left(v_{1}^{m}\right)=h\left({\left[D_{n}\right]}_{m}^{1}R_{n}u_{n}^{1}\right)=\sum_{i=1}^{m}\log d_{n,i}+h\left({\left[R_{n}\right]}_{m}^{1}u_{n}^{1}\right). \end{array}$$

Then, by inserting (A43) and (A42) in (A37), dividing by *n*, and taking the limit n→∞, we obtain

(A44) $$\begin{array}{c} \lim_{n\to \infty }\frac{1}{n}h\left(w_{m+1}^{n}|w_{1}^{m}\right)\geq \lim_{n\to \infty }\frac{1}{n}\left(h\left(u_{1}^{n}\right)-\sum_{i=1}^{m}\log d_{n,i}-h\left({\left[R_{n}\right]}_{m}^{1}u_{n}^{1}\right)\right) \end{array}$$

(A45) $$\begin{array}{cc} \; & =\bar{h}\left(u_{1}^{\infty }\right)-\lim_{n\to \infty }\frac{1}{n}\sum_{i=1}^{m}\log d_{n,i}, \end{array}$$

where the last equality is a consequence of the fact that $u_{1}^{\infty }$ is entropy balanced (specifically, from Proposition A3). We now derive an upper bound for $h(w_{m+1}^{n}|w_{1}^{m})$. Defining the random vector

$$x_{n}^{m+1}≜{\left[R_{n}\right]}_{n}^{m+1}u_{n}^{1},$$

and since $D_{n}$ is diagonal, we can write

(A46) $$\begin{array}{c} v_{m}^{1}={\left[D_{n}\right]}_{n}^{m+1}R_{n}u_{n}^{1}={}^{m+1}\;{\left[D_{n}\right]}_{n}x_{n}^{m+1}, \end{array}$$

where

(A47) $$\begin{array}{c} {}^{m+1}\;{\left[D_{n}\right]}_{n}≜\mathrm{diag}\left\{d_{n,m+1},d_{n,m+2},\ldots ,d_{n,n}\right\}. \end{array}$$

Therefore,

(A48) $$\begin{array}{c} h(w_{m+1}^{n}|w_{1}^{m})\leq h\left(w_{n}^{m+1}\right)=h\left({}^{m+1}\;{\left[D_{n}\right]}_{n}x_{n}^{m+1}+{\bar{z}}_{n}^{m+1}\right) \end{array}$$

(A49) $$\begin{array}{cc} \; & =\log\det\left({}^{m+1}\;{\left[D_{n}\right]}_{n}\right)+h\left(x_{n}^{m+1}+{\left({}^{m+1}\;{\left[D_{n}\right]}_{n}\right)}^{-1}{\bar{z}}_{n}^{m+1}\right). \end{array}$$

Notice that, by Assumption 2, ${\bar{z}}_{n}^{m+1}={\left[Q_{n}\right]}_{n}^{m+1}z_{n}^{1}={\left[Q_{n}\right]}_{n}^{m+1}{\left[\Phi \right]}_{n}^{1}s_{\kappa }^{1}$ and, thus, is restricted to the span of ${\left[Q_{n}\right]}_{n}^{m+1}{\left[\Phi \right]}_{n}^{1}$ of dimension $\kappa_{n}\leq \kappa$, for all n≥m+κ. Then, for every $n>m+\kappa_{n}$, one can construct a unitary matrix $H_{n}≜{\left(A_{n}^{T}|B_{n}^{T}\right)}^{T}\in R^{\left(n-m)\times (n-m\right)}$, with $A_{n}\in R^{\kappa \times (n-m)}$ and $B_{n}\in R^{\left(n-m-\kappa )\times (n-m\right)}$, such that the rows of $A_{n}$ span the space spanned by the columns of ${\left({}^{m+1}\;{\left[D_{n}\right]}_{n}\right)}^{-1}{\left[Q_{n}\right]}_{n}^{m+1}{\left[\Phi \right]}_{n}^{1}$ and such that $B_{n}{\left({}^{m+1}\;{\left[D_{n}\right]}_{n}\right)}^{-1}{\left[Q_{n}\right]}_{n}^{m+1}$${\left[\Phi \right]}_{n}^{1}=0$. Therefore, from (A49),

$$\begin{array}{cc} h(w_{m+1}^{n}|w_{1}^{m}) & \leq \log\det\left({}^{m+1}\;{\left[D_{n}\right]}_{n}\right)+h\left(H_{n}x_{n}^{m+1}+H_{n}{\left({}^{m+1}\;{\left[D_{n}\right]}_{n}\right)}^{-1}{\bar{z}}_{n}^{m+1}\right)\\ \; & =\log\det\left({}^{m+1}\;{\left[D_{n}\right]}_{n}\right)+h\left(B_{n}x_{n}^{m+1}\right)+h\left(A_{n}x_{n}^{m+1}+A_{n}{\left({}^{m+1}\;{\left[D_{n}\right]}_{n}\right)}^{-1}{\bar{z}}_{n}^{m+1}|B_{n}x_{n}^{m+1}\right)\\ \; & \leq \log\det\left({}^{m+1}\;{\left[D_{n}\right]}_{n}\right)+h\left(B_{n}x_{n}^{m+1}\right)+h\left(A_{n}x_{n}^{m+1}+A_{n}{\left({}^{m+1}\;{\left[D_{n}\right]}_{n}\right)}^{-1}{\bar{z}}_{n}^{m+1}\right)\\ \; & \leq \log\det\left({}^{m+1}\;{\left[D_{n}\right]}_{n}\right)+h\left(B_{n}x_{n}^{m+1}\right)+\frac{1}{2}\log\left({\left(2\pi e^{}\right)}^{\kappa }\det\left(K_{A_{n}x_{n}^{m+1}}+K_{A_{n}{\left({}^{m+1}{\left[D_{n}\right]}_{n}\right)}^{-1}{\bar{z}}_{n}^{m+1}}\right)\right)\\ \; & \leq \log\det\left({}^{m+1}\;{\left[D_{n}\right]}_{n}\right)+h\left(B_{n}x_{n}^{m+1}\right)+\frac{1}{2}\log\left({\left(2\pi e^{}\right)}^{\kappa }{\left[\lambda_{\max}\left(K_{x_{n}^{m+1}}\right)+\frac{\lambda_{\max}\left(K_{{\bar{z}}_{n}^{m+1}}\right)}{\lambda_{\min}{\left({}^{m+1}\;{\left[D_{n}\right]}_{n}\right)}^{2}}\right]}^{\kappa }\right), \end{array}$$

where $K_{A_{n}x_{n}^{m+1}}$ and $K_{A_{n}{\left({}^{m+1}{\left[D_{n}\right]}_{n}\right)}^{-1}{\bar{z}}_{n}^{m+1}}$ are the covariance matrices of $A_{n}x_{n}^{m+1}$ and $A_{n}{\left({}^{m+1}{\left[D_{n}\right]}_{n}\right)}^{-1}{\bar{z}}_{n}^{m+1}$, respectively, and where the last inequality follows from [31]. The fact that $\lambda_{\max}\left(K_{x_{n}^{m+1}}\right)$ and $\lambda_{\max}\left(K_{{\bar{z}}_{n}^{m+1}}\right)$ are upper bounded for all *n*, and the fact that $\lambda_{\min}\left({}^{m+1}\;{\left[D_{n}\right]}_{n}\right)$ either grows with *n* or decreases sub-exponentially (from Lemma 7), imply that

(A50) $$\begin{array}{c} \lim_{n\to \infty }\frac{1}{n}h\left(w_{m+1}^{n}|w_{1}^{m}\right)\leq \lim_{n\to \infty }\frac{1}{n}\log\det\left({}^{m+1}\;{\left[D_{n}\right]}_{n}\right)+\lim_{n\to \infty }\frac{1}{n}h\left(B_{n}x_{n}^{m+1}\right). \end{array}$$

But the fact that $\det D_{n}=1$ implies that $\log\det\left({}^{m+1}\;{\left[D_{n}\right]}_{n}\right)=-\sum_{i=1}^{m}\log d_{n,i}$. On the other hand, recalling that $x_{n}^{m+1}={\left[R_{n}\right]}_{n}^{m+1}u_{n}^{1}$ and noting that $B_{n}{\left[R_{n}\right]}_{n}^{m+1}$ has orthonormal rows, reveals that $\lim_{n\to \infty }\frac{1}{n}h\left(B_{n}x_{n}^{m+1}\right)=\bar{h}\left(u_{1}^{\infty }\right)$ (from the assumption that $u_{1}^{\infty }$ is entropy balanced). Therefore,

(A51) $$\begin{array}{c} \lim_{n\to \infty }\frac{1}{n}h\left(w_{m+1}^{n}|w_{1}^{m}\right)\leq \bar{h}\left(u_{1}^{\infty }\right)-\lim_{n\to \infty }\frac{1}{n}\sum_{i=1}^{m}\log d_{n,i}, \end{array}$$

which coincides with the lower bound found in (A45), completing the proof.   □

**Proof** **of Lemma 7.** The transfer function G(z) can be factored as $G\left(z\right)=\tilde{G}\left(z\right)F\left(z\right)$, where $\tilde{G}\left(z\right)$ is stable and minimum phase and F(z) is stable with all the non-minimum phase zeros of G(z), both being biproper rational functions. From Lemma A2 (in Appendix C), in the limit as n→∞, the eigenvalues of ${\tilde{G}}_{n}^{T}{\tilde{G}}_{n}$ are lower and upper bounded by $\lambda_{\min}\left({\tilde{G}}^{T}\tilde{G}\right)$ and $\lambda_{\max}\left({\tilde{G}}^{T}\tilde{G}\right)$, respectively, where $0<\lambda_{\min}\left({\tilde{G}}^{T}\tilde{G}\right)\leq \lambda_{\max}\left({\tilde{G}}^{T}\tilde{G}\right)<\infty$. Let ${\tilde{G}}_{n}={\tilde{Q}}_{n}^{T}{\tilde{D}}_{n}{\tilde{R}}_{n}$ and $F_{n}=Q_{n}^{T}D_{n}R_{n}$ be the SVDs of ${\tilde{G}}_{n}$ and $F_{n}$, respectively, with ${\tilde{d}}_{n,1}\leq {\tilde{d}}_{n,2}\leq \cdots \leq {\tilde{d}}_{n,n}$ and $d_{n,1}\leq d_{n,2}\leq \cdots \leq d_{n,n}$ being the diagonal entries of the diagonal matrices ${\tilde{D}}_{n}$, $D_{n}$, respectively. Then,

(A52) $$\begin{array}{c} G_{n}^{T}G_{n}=F_{n}^{T}{\tilde{G}}_{n}^{T}{\tilde{G}}_{n}F_{n}={\left({\tilde{D}}_{n}{\tilde{R}}_{n}Q_{n}^{T}D_{n}R_{n}\right)}^{T}{\tilde{D}}_{n}{\tilde{R}}_{n}Q_{n}^{T}D_{n}R_{n}. \end{array}$$

Denoting the *i*-th row of $R_{n}$ by $r_{n,i}^{T}$ be, we have that, from the Courant-Fischer theorem [30] that

(A53) $$\begin{array}{c} \lambda_{i}\left(G_{n}^{T}G_{n}\right)\leq \max_{v\in \mathrm{span}{\left\{r_{n,k}\right\}}_{k=1}^{i}\;:\;∥v∥=1}{∥Gv∥}^{2} \end{array}$$

(A54) $$\begin{array}{cc} & =\max_{v\in \mathrm{span}{\left\{r_{n,k}\right\}}_{k=1}^{i}\;:\;∥v∥=1}{∥{\tilde{D}}_{n}{\tilde{R}}_{n}^{T}Q_{n}^{T}D_{n}R_{n}v∥}^{2} \end{array}$$

(A55) $$\begin{array}{cc} & \leq d_{n,i}^{2}{\tilde{d}}_{n,n}^{2}. \end{array}$$

Likewise,

(A56) $$\begin{array}{c} \lambda_{i}\left(G_{n}^{T}G_{n}\right)\geq \min_{v\in \mathrm{span}{\left\{r_{n,k}\right\}}_{k=i}^{n}\;:\;∥v∥=1}∥Gv∥ \end{array}$$

(A57) $$\begin{array}{cc} & =\min_{v\in \mathrm{span}{\left\{r_{n,k}\right\}}_{k=i}^{n}\;:\;∥v∥=1}{∥{\tilde{D}}_{n}{\tilde{R}}_{n}^{T}Q_{n}^{T}D_{n}R_{n}v∥}^{2} \end{array}$$

(A58) $$\begin{array}{cc} & \geq d_{n,i}^{2}{\tilde{d}}_{n,1}^{2}. \end{array}$$

Thus,

(A59) $$\begin{array}{c} \lim_{n\to \infty }\frac{\lambda_{i}\left(G_{n}^{T}G_{n}\right)}{d_{n,i}^{2}}\in \left(\lambda_{\min}\left({\tilde{G}}^{T}\tilde{G}\right)\;,\;\lambda_{\max}\left({\tilde{G}}^{T}\tilde{G}\right)\right). \end{array}$$

The result now follows directly from Lemma A3 (in Appendix C).   □

**Proof** **of Theorem 6.** To begin with, the entropy power inequality [1] gives $h\left(y_{n}^{1}\right)=h\left(G_{n}u_{n}^{1}+z_{n}^{1}\right)\geq h\left(G_{n}u_{n}^{1}\right)=h\left(y_{n}^{1}\right)$, proving the lower bound in (70). To obtain the other bounds on the entropy gain of $G_{n}$, we will use Lemma 6. Recalling the structure of $z_{1}^{\infty }$ specified in Assumption 2, the random vector whose differential entropy appears on the RHS of (64) takes the form

(A60) $$\begin{array}{c} {\left[D_{n}\right]}_{m}^{1}R_{n}u_{n}^{1}+{\left[Q_{n}\right]}_{m}^{1}z_{n}^{1}={\left[D_{n}\right]}_{m}^{1}R_{n}u_{n}^{1}+{\left[Q_{n}\right]}_{m}^{1}{\left[\Phi \right]}_{n}^{1}s_{\kappa }^{1}. \end{array}$$

Notice that, for every n≥κ, the columns of the matrix ${\left[Q_{n}\right]}_{m}^{1}{\left[\Phi \right]}_{n}^{1}\in R^{m\times \kappa }$ span a space of dimension $\kappa_{n}\in \left\{0,1,\ldots ,\bar{\kappa }\right\}$, with $\bar{\kappa }≜\min\left\{m,\kappa \right\}$. If $\kappa_{n}=0$ (i.e., ${\left[Q_{n}\right]}_{m}^{1}{\left[\Phi \right]}_{n}^{1}=0$), then

(A61) $$\begin{array}{c} h\left({\left[D_{n}\right]}_{m}^{1}R_{n}u_{n}^{1}+{\left[Q_{n}\right]}_{m}^{1}z_{n}^{1}\right)=h\left({\left[D_{n}\right]}_{m}^{1}R_{n}u_{n}^{1}\right). \end{array}$$

If that is that case for every n≥κ, the lower bound in (70) is reached by inserting the latter expression into (64) and invoking Lemma 7. Let ${\left[Q_{n}\right]}_{m}^{1}{\left[\Phi \right]}_{n}^{1}=A_{n}^{T}T_{n}B_{n}$ be an SVD for ${\left[Q_{n}\right]}_{m}^{1}{\left[\Phi \right]}_{n}^{1}$, where $A_{n}\in R^{\kappa_{n}\times m}$ has orthonormal rows,

$$T_{n}=\mathrm{diag}\left\{t_{1}\left(n\right),t_{2}\left(n\right),\ldots ,t_{\kappa_{n}}\left(n\right)\right\},$$

where $0<t_{1}\left(n\right)\leq t_{2}\left(n\right)\leq \cdots \leq t_{\kappa_{n}}\left(n\right)\leq 1$ are the singular values of ${\left[Q_{n}\right]}_{m}^{1}{\left[\Phi \right]}_{n}^{1}$, and $B_{n}\in R^{\kappa_{n}\times \kappa }$ has orthonormal rows. Construct a unitary matrix $H_{n}\in R^{m\times m}$ such that

(A62) $$\begin{array}{c} H_{n}≜\left(\begin{array}{c} A_{n}\\ {\bar{A}}_{n} \end{array}\right), \end{array}$$

where $A_{n}\in R^{\kappa_{n}\times m}$ is as before, and ${\bar{A}}_{n}\in R^{(m-\kappa_{n})\times m}$ has orthonormal rows, and its row span is the orthogonal complement of that of $A_{n}$. Thus,

(A63) $$\begin{array}{c} H_{n}{\left[Q_{n}\right]}_{m}^{1}{\left[\Phi \right]}_{n}^{1}=\left(\begin{array}{c} A_{n}{\left[Q_{n}\right]}_{m}^{1}{\left[\Phi \right]}_{n}^{1}\\ 0_{(m-\kappa_{n})\times \kappa } \end{array}\right),\;\;\;\;n\geq N. \end{array}$$

From (A63) and (A60), we obtain

(A64) $$\begin{array}{c} h\left({\left[D_{n}\right]}_{m}^{1}R_{n}u_{n}^{1}+{\left[Q_{n}\right]}_{m}^{1}z_{n}^{1}\right)=h\left({\left[D_{n}\right]}_{m}^{1}R_{n}u_{n}^{1}+{\left[Q_{n}\right]}_{m}^{1}{\left[\Phi \right]}_{n}^{1}s_{\kappa }^{1}\right) \end{array}$$

(A65) $$\begin{array}{cc} \; & =h\left(H_{n}\left({\left[D_{n}\right]}_{m}^{1}R_{n}u_{n}^{1}+{\left[Q_{n}\right]}_{m}^{1}{\left[\Phi \right]}_{n}^{1}s_{\kappa }^{1}\right)\right) \end{array}$$

(A66) $$\begin{array}{cc} \; & =h\left(A_{n}{\left[D_{n}\right]}_{m}^{1}R_{n}u_{n}^{1}+A_{n}{\left[Q_{n}\right]}_{m}^{1}{\left[\Phi \right]}_{n}^{1}s_{\kappa }^{1}\;|\;{\bar{A}}_{n}{\left[D_{n}\right]}_{m}^{1}R_{n}u_{n}^{1}\right)+\left(1-1_{\left\{m\right\}}\left(\kappa_{n}\right)\right)h\left({\bar{A}}_{n}{\left[D_{n}\right]}_{m}^{1}R_{n}u_{n}^{1}\right), \end{array}$$

where the indicator function $1_{\left\{m\right\}}\left(\kappa_{n}\right)=1$ if $\kappa_{n}=m$ and 0 otherwise. The first differential entropy on the RHS of (A66) can be lower bounded as

(A67) $$\begin{array}{c} h\left(A_{n}{\left[D_{n}\right]}_{m}^{1}R_{n}u_{n}^{1}+A_{n}{\left[Q_{n}\right]}_{m}^{1}{\left[\Phi \right]}_{n}^{1}s_{\kappa }^{1}\;|\;{\bar{A}}_{n}{\left[D_{n}\right]}_{m}^{1}R_{n}u_{n}^{1}\right)\overset{\left(a\right)}{\geq }h\left(A_{n}{\left[Q_{n}\right]}_{m}^{1}{\left[\Phi \right]}_{n}^{1}s_{\kappa }^{1}\;|\;{\bar{A}}_{n}{\left[D_{n}\right]}_{m}^{1}R_{n}u_{n}^{1}\right)\\ \overset{\left(b\right)}{=}h\left(A_{n}{\left[Q_{n}\right]}_{m}^{1}{\left[\Phi \right]}_{n}^{1}s_{\kappa }^{1}\right)=h\left(T_{n}B_{n}s_{\kappa }^{1}\right)\overset{\left(c\right)}{\geq }\kappa_{n}\log\left(t_{1}\left(n\right)\right)+h\left(s_{\kappa }^{1}\right)-\frac{\kappa -\kappa_{n}}{2}\log\left(\lambda_{\max}\left(K_{s_{\kappa }^{1}}\right)\right), \end{array}$$

where (a) is from the entropy power inequality [1], (b) holds because $s_{\kappa }^{1}⫫u_{n}^{1}$ and (c) is from Proposition A1. An upper bound can be obtained as

(A68) $$\begin{array}{c} h\left(A_{n}{\left[D_{n}\right]}_{m}^{1}R_{n}u_{n}^{1}+A_{n}{\left[Q_{n}\right]}_{m}^{1}{\left[\Phi \right]}_{n}^{1}s_{\kappa }^{1}\;|\;{\bar{A}}_{n}{\left[D_{n}\right]}_{m}^{1}R_{n}u_{n}^{1}\right)\overset{\left(a\right)}{\leq }h\left(A_{n}{\left[D_{n}\right]}_{m}^{1}R_{n}u_{n}^{1}+T_{n}B_{n}s_{\kappa }^{1}\right)\\ \overset{\left(b\right)}{\leq }\frac{1}{2}\log\left({\left(2\pi e^{}\right)}^{m}\det\left(K_{A_{n}{\left[D_{n}\right]}_{m}^{1}R_{n}u_{n}^{1}}+K_{T_{n}B_{n}s_{\kappa }^{1}}\right)\right)\\ \overset{\left(c\right)}{\leq }\frac{\kappa_{n}}{2}\log\left(\left(2\pi e^{}\right)\left({\left(d_{m}\right)}^{2}\lambda_{\max}\left(K_{u_{n}^{1}}\right)+{\left(t_{\kappa_{n}}\left(n\right)\right)}^{2}\lambda_{\max}\left(K_{s_{\kappa }^{1}}\right)\right)\right), \end{array}$$

where (a) holds because conditioning does not increase entropy, (b) is because a Gaussian distribution maximizes the differential entropy for a given covariance matrix, and (c) is due to Reference [31]. Notice that $u_{1}^{\infty }$ satisfies the requirements of Proposition A2, implying that $\lim_{n\to \infty }n^{-1}\lambda_{\max}\left(K_{u_{n}^{1}}\right)=0$. Thus, since $t_{\kappa_{n}}\left(n\right)\leq 1$, it follows from (A67), (A68), and (A66) that

(A69) $$\begin{array}{c} \lim_{n\to \infty }\frac{1-1_{\left\{m\right\}}\left(\kappa_{n}\right)}{n}h\left({\bar{A}}_{n}{\left[D_{n}\right]}_{m}^{1}R_{n}u_{n}^{1}\right)+\lim_{n\to \infty }\frac{\kappa_{n}}{n}\log\left(t_{1}\left(n\right)\right)\\ \leq \lim_{n\to \infty }\frac{1}{n}h\left({\left[D_{n}\right]}_{m}^{1}R_{n}u_{n}^{1}+{\left[Q_{n}\right]}_{m}^{1}z_{n}^{1}\right)\leq \lim_{n\to \infty }\left(1-1_{\left\{m\right\}}\left(\kappa_{n}\right)\right)h\left({\bar{A}}_{n}{\left[D_{n}\right]}_{m}^{1}R_{n}u_{n}^{1}\right). \end{array}$$

For the last differential entropy on the RHS of (A66), notice that ${\left[D_{n}\right]}_{m}^{1}R_{n}={}^{1}\;{\left[D_{n}\right]}_{m}{\left[R_{n}\right]}_{m}^{1}$. Consider the SVD ${\bar{A}}_{n}{}^{1}\;{\left[D_{n}\right]}_{m}{\left[R_{n}\right]}_{m}^{1}=V_{n}^{T}\Sigma_{n}W_{n}$, with $V_{n}\in R^{\left(m-\kappa_{n}\right)\times \left(m-\kappa_{n}\right)}$ being unitary, $\Sigma_{n}\in R^{\left(m-\kappa_{n}\right)\times \left(m-\kappa_{n}\right)}$ being diagonal, and $W_{n}\in R^{(m-\kappa_{n})\times n}$ having orthonormal rows. We can then conclude that

(A70) $$\begin{array}{c} h\left({\bar{A}}_{n}{\left[D_{n}\right]}_{m}^{1}R_{n}u_{n}^{1}\right)=h\left(\Sigma_{n}W_{n}u_{n}^{1}\right)=\log\left|\det(\Sigma_{n})\right|+h\left(W_{n}u_{n}^{1}\right). \end{array}$$

Now, the fact that

$$\begin{array}{c} {\bar{A}}_{n}{}^{1}\;{\left[D_{n}\right]}_{m}{}^{1}\;{\left[D_{n}\right]}_{m}{\bar{A}}_{n}^{T}={\bar{A}}_{n}{}^{1}\;{\left[D_{n}\right]}_{m}{\left[R_{n}\right]}_{m}^{1}{\left({\bar{A}}_{n}{}^{1}\;{\left[D_{n}\right]}_{m}{\left[R_{n}\right]}_{m}^{1}\right)}^{T}=V^{T}\Sigma WW^{T}\Sigma^{T}V=V^{T}\Sigma \Sigma^{T}V \end{array}$$

reveals that

(A71) $$\begin{array}{c} \log\left|\det\Sigma \;\right|=\frac{1}{2}\log\left|\det\left({\bar{A}}_{n}{\left({}^{1}\;{\left[D_{n}\right]}_{m}\right)}^{2}{\bar{A}}_{n}^{T}\right)\right|. \end{array}$$

Recalling that ${\bar{A}}_{n}={\left[H_{n}\right]}_{m}^{\kappa_{n}+1}$ and that $H_{n}\in R^{m\times m}$ is unitary, it is easy to show (by using the Courant-Fischer theorem [30]) that

(A72) $$\begin{array}{c} \sum_{i=1}^{m-\kappa_{n}}\log d_{n,i}\overset{\left(a\right)}{\leq }\frac{1}{2}\log\left|\det({\bar{A}}_{n}{\left({}^{1}\;{\left[D_{n}\right]}_{m}\right)}^{2}{\bar{A}}_{n}^{T})\right|\overset{\left(b\right)}{\leq }\sum_{i=\kappa_{n}+1}^{m}\log d_{n,i}, \end{array}$$

with equality in (a) and (b) if and only if ${\bar{A}}_{n}=\left[I_{m-\kappa_{n}}\;|\;0\right]$ and ${\bar{A}}_{n}=\left[0\;|\;I_{m-\kappa_{n}}\right]$, respectively. Substituting this into (A71) and then the latter into (A70), we arrive to

(A73) $$\begin{array}{c} h\left({\left[W_{n}\right]}_{m}^{1}u_{n}^{1}\right)+\sum_{i=1}^{m-\kappa_{n}}\log d_{n,i}\leq h\left({\bar{A}}_{n}{\left[D_{n}\right]}_{m}^{1}R_{n}u_{n}^{1}\right)\leq h\left({\left[W_{n}\right]}_{m}^{1}u_{n}^{1}\right)+\sum_{i=\kappa_{n}+1}^{m}\log d_{n,i}. \end{array}$$

Substituting the upper bound from this equation and from (A68) into (A66) and the latter in (64), exploiting the fact that $u_{1}^{\infty }$ is entropy balanced (which ensures that $u_{1}^{\infty }$ satisfies Condition i) in Definition 2) and invoking Lemma 7 yields the upper bound in (70). Doing the same substitutions but with the lower bounds in (A73) and (A67), and using the assumption that $\lim_{n\to \infty }\frac{1}{n}\log\left(t_{1}\left(n\right)\right)=0$, gives the lower bound of (71). This completes the proof.   □

**Proof** **of Lemma 8.** We will consider first the case κ=m and show that $\lim_{n\to \infty }\sigma_{\min}$$({}^{1}\;{\left[Q_{n}\right]}_{m})>0$, where now $Q_{n}^{T}$ is the left unitary matrix in the SVD $F_{n}=Q_{n}^{T}D_{n}R_{n}$. We will prove that this is the case by using a contradiction argument. Thus, suppose the contrary, i.e., that

(A74) $$\begin{array}{c} \lim_{n\to \infty }\sigma_{\min}\left({}^{1}\;{\left[Q_{n}\right]}_{m}\right)=0. \end{array}$$

Then, there exists a sequence of unit-norm vectors ${\left\{v_{n}\right\}}_{n=1}^{\infty }$, with $v_{n}\in R^{m}$ for all *n*, such that

(A75) $$\begin{array}{c} \lim_{n\to \infty }∥v_{n}^{T}\;{}^{1}\;{\left[Q_{n}\right]}_{m}∥=0. \end{array}$$

For each n∈N, define the *n*-length unit-norm image vectors $t_{n}^{T}≜v_{n}^{T}{\left[Q_{n}\right]}_{m}^{1}$. Then,

(A76) $$\begin{array}{c} ∥F_{n}^{T}t_{n}∥=∥R_{n}^{T}D_{n}Q_{n}t_{n}∥=∥D_{n}Q_{n}t_{n}∥=∥{}^{1}\;{\left[D_{n}\right]}_{m}v_{n}∥, \end{array}$$

where the last equality follows from the fact that, by construction, $t_{n}^{T}$ is in the span of the first *m* rows of $Q_{n}$, together with the fact that $Q_{n}$ is unitary (which implies that ${\left[Q_{n}\right]}_{n}^{m+1}t_{n}=0$). Since the top *m* entries in $D_{n}$ decay exponentially as *n* increases, we have that

(A77) $$\begin{array}{c} ∥F_{n}^{T}t_{n}∥\leq O(\zeta_{n}|\rho_{M}{|}^{-n}), \end{array}$$

where $\zeta_{n}$ is a finite-order polynomial of *n* (from Lemma A3, in Appendix C). Now, notice that ${\left[F_{n}\right]}_{n}^{m+1}{\left({\left[F_{n}\right]}_{n}^{m+1}\right)}^{T}$ is a Toeplitz matrix with the convolution of *f* and $f^{-}$ (the impulse response of *F* and its time-reversed version, respectively) on its first row and column. It then follows from Reference [18], Lemma 4.1, that

(A78) $$\begin{array}{c} \lim_{n\to \infty }\lambda_{\min}\left({\left[F_{n}\right]}_{n}^{m+1}{\left({\left[F_{n}\right]}_{n}^{m+1}\right)}^{T}\right)=\min_{\omega :\omega \in [-\pi ,\pi ]}{\left|F\left(e^{j\omega }\right)\right|}^{2}>0 \end{array}$$

(the inequality is strict because all the zeros of F(z) are strictly outside the unit disk). Then, we conclude that

(A79) $$\begin{array}{c} \lim_{n\to \infty }\sigma_{\min}\left({\left[F_{n}\right]}_{n}^{m+1}\right)>0. \end{array}$$

Recall that $∥t_{n}∥=1$; thus, from (A75), $\lim_{n\to \infty }∥{\left[t_{n}\right]}_{m}^{1}∥=0$ and $\lim_{n\to \infty }∥{\left[t_{n}\right]}_{n}^{m+1}∥=1$, which means that $\lim_{n\to \infty }∥F_{n}^{T}t_{n}∥=\lim_{n\to \infty }∥{\left({\left[F_{n}\right]}_{n}^{m+1}\right)}^{T}{\left[t_{n}\right]}_{n}^{m+1}∥\geq \lim_{n\to \infty }\sigma_{\min}\left({\left[F_{n}\right]}_{n}^{m+1}\right)\overset{\left(79\right)}{>}0$, which contradicts (A77). Therefore,

(A80) $$\begin{array}{c} \lim_{n\to \infty }\sigma_{\min}\left({}^{1}\;{\left[Q_{n}\right]}_{m}\right)>0. \end{array}$$

Now, consider an arbitrary κ≥1. Since

(A81) $$\begin{array}{c} \sigma_{\min}\left(\overset{1\;\;\kappa }{\overset{⎴}{{\left[Q_{n}\right]}_{m}^{1}}}\right)\geq \sigma_{\min}\left({}^{1}\;{\left[Q_{n}\right]}_{m}\right), \end{array}$$

it follows from (A80) that

(A82) $$\begin{array}{c} \lim_{n\to \infty }\sigma_{\min}\left({\left[Q_{n}\right]}_{m}^{1}{\left[\Phi \right]}_{n}^{1}\right)=\lim_{n\to \infty }\sigma_{\min}\left(\overset{1\;\;\kappa }{\overset{⎴}{{\left[Q_{n}\right]}_{m}^{1}}}\right)>0; \end{array}$$

thus, $\lim_{n\to \infty }\kappa_{n}=\bar{\kappa }$. This completes the proof.   □

**Proof** **of Theorem 9.** Denote the Blaschke product [11] of A(z) as

(A83) $$\begin{array}{c} B\left(z\right)≜\frac{\prod_{i=1}^{m}\left(z-p_{i}\right)}{\prod_{i=1}^{m}p_{i}^{*}\left(z-1/p_{i}^{*}\right)}, \end{array}$$

which clearly satisfies

(A84) $$\begin{array}{cc} \left|B(e^{j\omega })\right| & =1,\;\;\;\;\;\forall \omega \in [-\pi ,\pi ], \end{array}$$

(A85) $$\begin{array}{cc} b_{0} & ≜\lim_{|z|\to \infty }B\left(z\right)=\frac{1}{\prod_{i=1}^{m}p_{i}^{*}}, \end{array}$$

where $b_{0}$ is the first sample in the impulse response of B(z). Notice that (A84) implies that $\lim_{n\to \infty }\frac{1}{n}E[∥B_{n}u_{n}^{1}{∥}^{2}]=\lim_{n\to \infty }\frac{1}{n}E[∥u_{n}^{1}{∥}^{2}]$ for every sequence of random variables $u_{1}^{\infty }$ with uniformly bounded variance. Since B(z) has only stable poles and its zeros coincide exactly with the poles of A(z), it follows that B(z)A(z) is an MP stable transfer function. Thus, the asymptotically stationary process ${\tilde{x}}_{1}^{\infty }$ defined in (139) can be constructed as

(A86) $$\begin{array}{c} {\tilde{x}}_{n}^{1}≜B_{n}x_{n}^{1}, \end{array}$$

where $B_{n}$ is a Toeplitz lower triangular matrix with its main diagonal entries equal to $b_{0}$. Since $w_{1}^{\infty }$ is entropy balanced, so is ${\tilde{x}}_{1}^{\infty }$, thanks to Lemma 4. The fact that B(z) is biproper with $b_{0}$ as in (A85) implies that, for any $u_{n}^{1}$ with finite differential entropy,

(A87) $$\begin{array}{c} h\left(B_{n}u_{n}^{1}\right)=h\left(u_{n}^{1}\right)-n\underset{≜G}{\underbrace{\sum_{i=1}^{m}\log\left|p_{i}\right|}}, \end{array}$$

which will be utilized next. For any given n≥m, suppose that C(z) is chosen and $x_{n}^{1}$ and $u_{n}^{1}$ are distributed so as to minimize $I(x_{n}^{1};C_{n}x_{n}^{1}+u_{n}^{1})$ subject to the constraint $E[∥y_{n}^{1}-x_{n}^{1}{∥}^{2}]=E[∥\left(C_{n}-I\right)x_{n}^{1}{∥}^{2}]+E[∥u_{n}^{1}{∥]}^{2}\leq D$ (i.e., $x_{n}^{1},u_{n}^{1}$ is a realization of $R_{x,n}\left(D\right)$), yielding the reconstruction

(A88) $$\begin{array}{c} y_{n}^{1}=C_{n}x_{n}^{1}+u_{n}^{1}. \end{array}$$

Since we are considering mean-squared error distortion, it follows that, for rate-distortion optimality, $u_{n}^{1}$ must be jointly Gaussian with $x_{n}^{1}$. In addition, there is no loss of rate-distortion optimality if $u_{1}^{\infty }$ is entropy balanced (otherwise, it would have a lower entropy rate than its entropy-balanced counterpart, which differs from the former only on a finite number of samples and has the same asymptotic MSE). From these vectors, define

(A89) $$\begin{array}{c} {\tilde{u}}_{n}^{1}≜B_{n}u_{n}^{1}, \end{array}$$

(A90) $$\begin{array}{c} {\tilde{y}}_{n}^{1}≜B_{n}y_{n}^{1}=B_{n}C_{n}{\left(B_{n}\right)}^{-1}{\tilde{x}}_{n}^{1}+{\tilde{u}}_{n}^{1}, \end{array}$$

(A91) $$\begin{array}{c} {\bar{y}}_{n}^{1}≜{\tilde{y}}_{n}^{1}+d_{n}^{1}=B_{n}C_{n}{\left(B_{n}\right)}^{-1}{\tilde{x}}_{n}^{1}+{\tilde{u}}_{n}^{1}+d_{n}^{1}, \end{array}$$

where $d_{n}^{1}$ is a zero-mean Gaussian vector independent of $\left({\tilde{u}}_{n}^{1},{\tilde{x}}_{n}^{1}\right)$ with finite differential entropy and finite variance such that $d_{k}=0$, ∀k>m. Then, we have that (the change of variables and the steps in this chain of equations is represented by the block diagrams shown in Figure 7)

(A92) $$\begin{array}{c} nR_{x,n}\left(D\right)=I\left(x_{n}^{1};y_{n}^{1}\right)\overset{\left(a\right)}{=}I\left(B_{n}x_{n}^{1};B_{n}y_{n}^{1}\right)=I\left({\tilde{x}}_{n}^{1};{\tilde{y}}_{n}^{1}\right) \end{array}$$

(A93) $$\begin{array}{cc} \; & =h\left({\tilde{y}}_{n}^{1}\right)-h\left({\tilde{y}}_{n}^{1}|{\tilde{x}}_{n}^{1}\right) \end{array}$$

(A94) $$\begin{array}{cc} \; & \overset{\left(b\right)}{=}h\left({\tilde{y}}_{n}^{1}\right)-h\left({\tilde{u}}_{n}^{1}|{\tilde{x}}_{n}^{1}\right) \end{array}$$

(A95) $$\begin{array}{cc} \; & \overset{\left(c\right)}{=}h\left({\tilde{y}}_{n}^{1}\right)-h\left({\tilde{u}}_{n}^{1}\right) \end{array}$$

(A96) $$\begin{array}{cc} \; & \overset{\left(d\right)}{=}h\left({\tilde{y}}_{n}^{1}\right)-\left(h\left({\tilde{u}}_{n}^{1}+d_{n}^{1}\right)+\left[h\left(u_{n}^{1}\right)-h\left({\tilde{u}}_{n}^{1}+d_{n}^{1}\right)\right]-nG\right) \end{array}$$

(A97) $$\begin{array}{cc} \; & \overset{\left(e\right)}{=}h\left({\tilde{y}}_{n}^{1}\right)-h\left({\tilde{u}}_{n}^{1}+d_{n}^{1}|{\tilde{x}}_{n}^{1}\right)+nG-\left[h\left(u_{n}^{1}\right)-h\left({\tilde{u}}_{n}^{1}+d_{n}^{1}\right)\right] \end{array}$$

(A98) $$\begin{array}{cc} \; & \overset{\left(f\right)}{=}h\left({\tilde{y}}_{n}^{1}\right)-h\left({\bar{y}}_{n}^{1}|{\bar{x}}_{n}^{1}\right)+nG-\left[h\left(u_{n}^{1}\right)-h\left({\tilde{u}}_{n}^{1}+d_{n}^{1}\right)\right] \end{array}$$

(A99) $$\begin{array}{cc} \; & =h\left({\tilde{y}}_{n}^{1}\right)-h\left({\bar{y}}_{n}^{1}\right)+I\left({\tilde{x}}_{n}^{1};{\bar{y}}_{n}^{1}\right)+nG-\left[h\left(u_{n}^{1}\right)-h\left({\tilde{u}}_{n}^{1}+d_{n}^{1}\right)\right] \end{array}$$

(A100) $$\begin{array}{cc} \; & =I\left({\tilde{x}}_{n}^{1};{\bar{y}}_{n}^{1}\right)+nG-\left[h\left(u_{n}^{1}\right)-h\left({\tilde{u}}_{n}^{1}+d_{n}^{1}\right)\right]+\left[h\left({\tilde{y}}_{n}^{1}\right)-h\left({\tilde{y}}_{n}^{1}+d_{n}^{1}\right)\right], \end{array}$$

where (a) follows from $B_{n}$ being invertible, (b) is due to the fact that ${\tilde{y}}_{n}^{1}=P_{n}{\tilde{x}}_{n}^{1}+{\tilde{u}}_{n}^{1}$, (c) holds because $u_{n}^{1}⫫x_{n}^{1}$. The equality (d) stems from $h\left({\tilde{u}}_{n}^{1}\right)=h\left(u_{n}^{1}\right)-nG$ (see (A87)). Equality holds in (e) because ${\tilde{x}}_{n}^{1}⫫\left({\tilde{u}}_{n}^{1},d_{n}^{1}\right)$ and in (f) because of (A91). But from Theorem 4 and since $u_{1}^{\infty }$ is entropy balanced, $\lim_{n\to \infty }\frac{1}{n}\left(h\left({\tilde{u}}_{n}^{1}+d_{m}^{1}\right)-h\left(u_{n}^{1}\right)\right)=0$. From Lemma 3 and because $u_{1}^{\infty }$ is entropy balanced, so is ${\tilde{y}}_{1}^{\infty }$. This guarantees, from Lemma 5, that $\lim_{n\to \infty }n^{-1}\left[h\left({\tilde{y}}_{n}^{1}\right)-h\left({\tilde{y}}_{n}^{1}+d_{n}^{1}\right)\right]=0$. Thus, $R_{x,n}\left(D\right)=\lim_{n\to \infty }\frac{1}{n}\left({\tilde{x}}_{n}^{1};{\bar{y}}_{n}^{1}\right)+G\geq R_{\tilde{x},n}\left(D\right)+G$. At the same time, the distortion for the source ${\tilde{x}}_{n}^{1}$ when reconstructed as ${\bar{y}}_{n}^{1}$ is

(A101) $$\begin{array}{c} \lim_{n\to \infty }\frac{1}{n}E\left[∥{\bar{y}}_{n}^{1}-{\tilde{x}}_{n}^{1}{∥}^{2}\right]=\lim_{n\to \infty }\frac{1}{n}\left(E\left[∥\tilde{y}-{\tilde{x}}_{n}^{1}{∥}^{2}\right]+E\left[∥d_{n}^{1}{∥}^{2}\right]\right)\overset{\left(a\right)}{=}\lim_{n\to \infty }\frac{1}{n}E\left[∥\tilde{y}-{\tilde{x}}_{n}^{1}{∥}^{2}\right] \end{array}$$

(A102) $$\begin{array}{cc} & =\lim_{n\to \infty }\frac{1}{n}E\left[∥B_{n}\left(y_{n}^{1}-x_{n}^{1}\right){∥}^{2}\right]\overset{\left(b\right)}{=}\lim_{n\to \infty }\frac{1}{n}E\left[∥y_{n}^{1}-x_{n}^{1}{∥}^{2}\right], \end{array}$$

where (a) holds because $∥d_{n}^{1}∥=∥d_{m}^{1}∥$ is bounded, and (b) is due to the fact that, in the limit, B(z) is a unitary operator. Recalling the definitions of $R_{\tilde{x}}\left(D\right)$ and $R_{\tilde{x}}\left(D\right)$, we conclude that $\lim_{n\to \infty }\frac{1}{n}\left({\tilde{x}}_{n}^{1};{\bar{y}}_{n}^{1}\right)\geq R_{\tilde{x},n}\left(D\right)$; therefore,

(A103) $$\begin{array}{c} R_{x}\left(D\right)-R_{\tilde{x}}\left(D\right)\geq \sum_{i=1}^{m}\log\left|p_{i}\right|. \end{array}$$

In order to complete the proof, it suffices to show that $R_{x}\left(D\right)-R_{\tilde{x}}\left(D\right)\leq \sum_{i=1}^{m}\log\left|p_{i}\right|$. For this purpose, consider now the (asymptotically) stationary source ${\tilde{x}}_{n}^{1}$, and suppose that ${\hat{y}}_{n}^{1}={\tilde{x}}_{n}^{1}+u_{n}^{1}$ realizes $R_{\tilde{x},n}\left(D\right)$. Again, ${\tilde{x}}_{n}^{1}$ and $u_{n}^{1}$ will be jointly Gaussian, satisfying ${\hat{y}}_{n}^{1}⫫u_{n}^{1}$ (the latter condition is required for minimum MSE optimality). From this, one can propose an alternative realization in which the error sequence is $\tilde{u}≜B_{n}u_{n}^{1}$, yielding an output ${\tilde{y}}_{n}^{1}={\tilde{x}}_{n}^{1}+{\tilde{u}}_{n}^{1}$ with ${\tilde{y}}_{n}^{1}⫫{\tilde{u}}_{n}^{1}$. Then,

(A104) $$\begin{array}{c} nR_{\tilde{x},n}\left(D\right)=I\left({\tilde{x}}_{n}^{1};{\hat{y}}_{n}^{1}\right)=h\left({\tilde{x}}_{n}^{1}\right)-h\left({\tilde{x}}_{n}^{1}|{\hat{y}}_{n}^{1}\right) \end{array}$$

(A105) $$\begin{array}{cc} & \overset{\left(a\right)}{=}h\left({\tilde{x}}_{n}^{1}\right)-h\left(u_{n}^{1}\right) \end{array}$$

(A106) $$\begin{array}{cc} & \overset{\left(b\right)}{=}h\left({\tilde{x}}_{n}^{1}\right)-h\left({\tilde{u}}_{n}^{1}\right)-nG \end{array}$$

(A107) $$\begin{array}{cc} & \overset{\left(c\right)}{=}h\left({\tilde{x}}_{n}^{1}\right)-h\left({\tilde{u}}_{n}^{1}|{\tilde{y}}_{n}^{1}\right)-nG \end{array}$$

(A108) $$\begin{array}{cc} & \overset{\left(d\right)}{=}h\left({\tilde{x}}_{n}^{1}\right)-h\left({\tilde{x}}_{n}^{1}|{\tilde{y}}_{n}^{1}\right)-nG \end{array}$$

(A109) =(a)I(x˜n1;y˜n1)−nG

(A110) =(a)I(Bnxn1;Bnyn1)−nG

(A111) $$\begin{array}{cc} & \overset{\left(e\right)}{=}I\left(x_{n}^{1};y_{n}^{1}\right)-nG, \end{array}$$

where (a) follows by recalling that ${\hat{y}}_{n}^{1}={\tilde{x}}_{n}^{1}+u_{n}^{1}$ and because ${\hat{y}}_{n}^{1}⫫u_{n}^{1}$, (b) stems from (A87), (c) is a consequence of ${\tilde{y}}_{n}^{1}⫫{\tilde{u}}_{n}^{1}$, (d) follows from the fact that ${\tilde{y}}_{n}^{1}={\tilde{x}}_{n}^{1}+{\tilde{u}}_{n}^{1}$. Finally, (e) holds because $B_{n}$ is invertible for all *n*. Since, asymptotically as n→∞, the distortion yielded by $y_{n}^{1}$ for the non-stationary source $x_{n}^{1}$ is the same which is obtained when ${\tilde{x}}_{n}^{1}$ is reconstructed as ${\hat{y}}_{n}^{1}$ (recall (A84)), we conclude that $R_{x}\left(D\right)-R_{\tilde{x}}\left(D\right)\leq \sum_{i=1}^{M}\log\left|p_{i}\right|$, completing the proof.   □

## Appendix C. Technical Lemmas and Propositions

**Proposition** **A1.**
*Let the random vector $s_{\kappa }^{1}$ have finite differential entropy, and suppose its covariance matrix $K_{s_{\kappa }^{1}}$ satisfies $\lambda_{\max}\left(K_{s_{\kappa }^{1}}\right)<\infty$. Then, for any unitary matrix $A\in R^{\kappa \times \kappa }$ and i=1,2,…,κ*

(A112) $$\begin{array}{c} h\left(s_{\kappa }^{1}\right)-\frac{\kappa -i}{2}\log\left(2\pi e^{}\lambda_{\max}\left(K_{s_{\kappa }^{1}}\right)\right)\leq h\left({\left[A\right]}_{i}^{1}s_{\kappa }^{1}\right)\leq \frac{i}{2}\log\left(2\pi e^{}\lambda_{\max}\left(K_{s_{\kappa }^{1}}\right)\right). \end{array}$$

**Proof.** Define $r_{\kappa }^{1}≜As_{\kappa }^{1}$. Since A is unitary, it follows that $h\left(r_{\kappa }^{1}\right)=h\left(s_{\kappa }^{1}\right)$ and that $K_{r_{\kappa }^{1}}$ and $K_{s_{\kappa }^{1}}$ have the same eigenvalues. Therefore,

(A113) $$\begin{array}{c} h\left({\left[A\right]}_{i}^{1}s_{\kappa }^{1}\right)=h\left(r_{i}^{1}\right)\overset{\left(a\right)}{\leq }\frac{1}{2}\log\left({\left(2\pi e^{}\right)}^{i}\det\left(K_{r_{i}^{1}}\right)\right)\overset{\left(b\right)}{\leq }\frac{i}{2}\log\left(2\pi e^{}\lambda_{\max}\left(K_{r_{i}^{1}}\right)\right)\overset{\left(c\right)}{\leq }\frac{i}{2}\log\left(2\pi e^{}\lambda_{\max}\left(K_{r_{\kappa }^{1}}\right)\right)\\ =\frac{i}{2}\log\left(2\pi e^{}\lambda_{\max}\left(K_{s_{\kappa }^{1}}\right)\right), \end{array}$$

where (a) holds because a Gaussian distribution yields the largest differential entropy for a given covariance matrix, (b) is from the fact that $\det\left(K_{s_{i}^{1}}\right)=\prod_{k=1}^{i}\lambda_{k}\left(K_{s_{i}^{1}}\right)$ and (c) is due to the Cauchy interlacing theorem [30]. This proves the upper bound in (A112). For the lower bound, we have

(A114) $$\begin{array}{c} h\left(r_{i}^{1}\right)\overset{\left(a\right)}{\geq }h\left(r_{\kappa }^{1}\right)-h\left(r_{i\kappa }^{i+1}\right)\overset{\left(b\right)}{\geq }h\left(r_{\kappa }^{1}\right)-\frac{\kappa -i}{2}\log\left(2\pi e^{}\lambda_{\max}\left(K_{s_{\kappa }^{1}}\right)\right), \end{array}$$

where (a) stems from the fact that h(a,b)≤h(a)+h(b) and (b) follows from (A113). This completes the proof.   □

**Proposition** **A2.**
*Let $u_{1}^{\infty }$ be a random process such that the variance of u(n), $\sigma_{u(n)}^{2}<\infty$ for finite n, and*

(A115) $$\begin{array}{c} \lim_{n\to \infty }\frac{1}{n}\log\left(\sigma_{u(n)}^{2}\right)=0. \end{array}$$

*Then, $\lim_{n\to \infty }n^{-1}\log\left(\lambda_{\max}\left(K_{u_{n}^{1}}\right)\right)=0$.          ▲*

**Proof.** The assumptions on $u_{1}^{\infty }$ imply that, for every ϵ>0, there exists a finite $N_{\epsilon }$ such that, for every $n\geq N_{\epsilon }$, $\sigma_{u(n)}^{2}<e^{n\epsilon }$ and $S\left(N_{\epsilon }\right)≜\max\left\{\sigma_{u(1)}^{2},\sigma_{u(2)}^{2},\ldots ,\sigma_{u(N_{\epsilon })}^{2}\right\}<\infty$. Then,

$$\begin{array}{c} \frac{1}{n}\ln\left(\lambda_{\max}\left(K_{u_{n}^{1}}\right)\right)\overset{\left(a\right)}{\leq }\frac{1}{n}\ln\left(\sum_{k=1}^{n}\sigma_{u(k)}^{2}\right)<\frac{1}{n}\ln\left(N_{\epsilon }S\left(N_{\epsilon }\right)+\left(n-N_{\epsilon }\right)e^{n\epsilon }\right)\overset{\left(b\right)}{<}\frac{1}{n}\ln\left(\left(n-N_{\epsilon }\right)e^{n\epsilon }\right)+\frac{N_{\epsilon }S\left(N_{\epsilon }\right)}{n\left(n-N_{\epsilon }\right)e^{n\epsilon }}, \end{array}$$

where (a) holds because $\sum_{k=1}^{n}\lambda_{k}\left(K_{u_{n}^{1}}\right)=\mathrm{tr}\left\{K_{u_{n}^{1}}\right\}$, while (b) stems from the fact that, for every x,y>0, ln(x+y)<ln(x)+y/x. Thus, for every ϵ>0, $\lim_{n\to \infty }n^{-1}\log\left(\lambda_{\max}\left(K_{u_{n}^{1}}\right)\right)<\epsilon$, which means that $\lim_{n\to \infty }n^{-1}\log\left(\lambda_{\max}\left(K_{u_{n}^{1}}\right)\right)=0$, completing the proof.   □

**Proposition** **A3.**
*Let $v_{1}^{\infty }$ be an entropy-balanced random process. Then, for each ν∈N and for every sequence of matrices ${\left\{\Psi_{n}\right\}}_{n=\nu }^{\infty }$, $\Psi_{n}\in R^{\nu \times n}$ with orthonormal rows,*

(A116) $$\begin{array}{c} \lim_{n\to \infty }\frac{1}{n}h(\Psi_{n}v_{n}^{1})=0. \end{array}$$

**Proof** **of Proposition A3.** We will first show that

(A117) $$\begin{array}{c} \lim_{n\to \infty }\frac{1}{n}h\left(\Psi_{n}v_{n}^{1}\right)\geq 0. \end{array}$$

To see this, notice that, for every $\Psi_{n}\in R^{\nu \times n}$ with orthonormal rows, there exists a matrix $\Phi_{n}\in R^{(n-\nu )\times n}$ with orthonormal rows which are also orthogonal to those of $\Psi_{n}$. This means that the matrix ${\left[\Psi_{n}^{T}|\Phi_{n}^{T}\right]}^{T}\in R^{n\times n}$ is unitary; thus,

(A118) $$\begin{array}{c} h\left(v_{n}^{1}\right)=h\left({\left[\Psi_{n}^{T}|\Phi_{n}^{T}\right]}^{T}v_{n}^{1}\right)\overset{\left(a\right)}{=}h\left(\Phi_{n}v_{n}^{1}\right)+h\left(\Psi_{n}v_{n}^{1}|\Phi_{n}v_{n}^{1}\right)\overset{\left(b\right)}{\leq }h\left(\Phi_{n}v_{n}^{1}\right)+h\left(\Psi_{n}v_{n}^{1}\right), \end{array}$$

where (a) holds due to the chain-rule of differential entropy and (b) follows because conditioning does not increase differential entropy. Therefore, $h\left(\Psi_{n}v_{n}^{1}\right)\geq h\left(v_{n}^{1}\right)-h\left(\Phi_{n}v_{n}^{1}\right)$. Dividing this by *n*, taking the limit when n→∞ and recalling that $v_{1}^{\infty }$ satisfies (17) yields (A117). We will now prove that $\lim_{n\to \infty }\frac{1}{n}h\left(\Psi_{n}v_{n}^{1}\right)\leq 0$. For this purpose, let ${\tilde{v}}_{1}^{\infty }$ be a jointly Gaussian random process with the same second-order statistics as $v_{1}^{\infty }$. Then,

(A119) $$\begin{array}{c} h\left(\Psi_{n}v_{n}^{1}\right)\leq h\left(\Psi_{n}{\tilde{v}}_{n}^{1}\right)=\frac{1}{2}\log\left({\left(2\pi e^{}\right)}^{\nu }\det\left(\Psi_{n}K_{{\tilde{v}}_{n}^{1}}\Psi_{n}^{T}\right)\right)\leq \frac{1}{2}\log\left({\left(2\pi e^{}\right)}^{\nu }\lambda_{\max}{\left(K_{{\tilde{v}}_{n}^{1}}\right)}^{\nu }\right), \end{array}$$

with the inequality due to the fact that $\Psi_{n}$ has orthonormal rows. But $v_{1}^{\infty }$ meets the requirements of Proposition A2; thus, $\lim_{n\to \infty }\frac{1}{n}h\left(\Psi_{n}v_{n}^{1}\right)\leq \lim_{n\to \infty }\frac{\nu }{2n}\left(2\pi e^{}\lambda_{\max}\left(K_{{\tilde{v}}_{n}^{1}}\right)\right)=0$. The proof is completed by combining this result with (A117).   □

**Lemma** **A1.**
*Let $u_{1}^{\infty }$ be a random process with independent elements, and where each element $u_{i}$ is uniformly distributed over possible different intervals $\left[-\frac{a_{i}}{2},\frac{a_{i}}{2}\right]$, such that $a_{\max}>\left|a_{i}\right|>a_{\min}>0,\forall i\in N$, for some positive and bounded $a_{\min}<a_{\max}$. Then, $u_{1}^{\infty }$ is entropy balanced.*

**Proof.** Without loss of generality, we can assume that $a_{i}\geq 1$, for all *i* (otherwise, we could scale the input by $1/a_{\min}$, which would scale the output by the same proportion, increasing the input entropy by $n\log(1/a_{\min})$ and the output entropy by $\left(n-\nu \right)\log\left(1/a_{\min}\right)$, without changing the result). The input vector $u_{n}^{1}$ is confined to an *n*-box $U_{n}$ (the support of $u_{1}^{n}$) of volume $V_{n}\left(U_{n}\right)=\prod_{i=1}^{n}a_{i}$ and has entropy $\log(\prod_{i=1}^{n}a_{i})$. This support is an *n*-box which contains nk2n−k*k*-boxes of different *k*-volume. Each of these *k*-boxes is determined by fixing n−k entries in $u_{n}^{1}$ to $\pm a_{i}/2$, and letting the remaining *k* entries sweep freely over $\left[-\frac{a_{i}}{2},\frac{a_{i}}{2}\right]$. Thus, the *k*-volume of each *k*-box is the product of the *k* support sizes $a_{i}$ of the associated selected free-sweeping entries. But recalling that $a_{i}>1$ for all *i*, the volume of each *k*-box can be upper bounded by $\prod_{i=1}^{n}a_{i}$. With this, the added volume of all the *k*-boxes contained in the original *n*-box can be upper bounded as

(A120) Vk□(Un)≤nk2n−k∏i=1nai.

We now use this result to upper bound the entropy rate of $y_{n}^{\nu +1}$. Let $y_{n}^{1}≜{\left[\Psi_{n}^{T}|\Phi_{n}^{T}\right]}^{T}u_{n}^{1}$ where ${\left[\Psi_{n}^{T}|\Phi_{n}^{T}\right]}^{T}\in R^{n\times n}$ is a unitary matrix and where $\Psi_{n}\in R^{\nu \times n}$ and $\Phi_{n}\in R^{(n-\nu )\times n}$ have orthonormal rows. From this definition, $y_{n}^{\nu +1}$ will distribute over a finite region $Y_{n}^{\nu +1}\subseteq R^{n-\nu }$, corresponding to the projection onto the (n−ν)-dimensional span of the rows of $\Phi_{n}$. Hence, $h(y_{n}^{\nu +1})$ is upper bounded by the entropy of a uniformly distributed vector over the same support, i.e., by $\log V_{n-\nu }\left(Y_{n}^{\nu +1}\right)$, where $V_{n-\nu }\left(Y_{n}^{\nu +1}\right)$ is the (n−ν)-dimensional volume of this support. In turn, $V_{n-\nu }\left(Y_{n}^{\nu +1}\right)$ is upper bounded by the sum of the volume of all (ν−k)-dimensional boxes contained in the *n*-box in which $u_{n}^{1}$ is confined, which we already denoted by $V_{n-\nu }^{□}\left(U_{n}\right)$, and which is upper bounded as in (A120). Therefore,

(A121) $$\begin{array}{c} h(y_{n}^{1+\nu })\leq \log V_{n-\nu }\left(Y_{n}^{\nu +1}\right)\leq \log V_{n-\nu }^{□}\left(U_{n}\right)\leq \log\left(\frac{n!}{(n-\nu )!\nu !}2^{\nu }\prod_{i=1}^{n}a_{i}\right) \end{array}$$

(A122) $$\begin{array}{cc} & =\log\left(n^{\nu }2^{\nu }\right)+\log\left(\frac{n!}{\left(n-\nu \right)!n^{\nu }\nu !}\right)+\log\left(\prod_{i=1}^{n}a_{i}\right). \end{array}$$

Recalling that $h\left(u_{n}^{1}\right)=\log\left(\prod_{i=1}^{n}a_{i}\right)$, dividing by *n* and taking the limit as n→∞ yields

(A123) $$\begin{array}{c} \lim_{n\to \infty }\frac{1}{n}\left(h\left(y_{n}^{\nu +1}\right)-h\left(u_{n}^{1}\right)\right)\leq 0. \end{array}$$

On the other hand,

(A124) $$\begin{array}{c} h\left(y_{n}^{\nu +1}\right)=h\left(y_{n}^{1}\right)-h\left(y_{\nu }^{1}|y_{n}^{\nu +1}\right)\overset{\left(a\right)}{=}h\left(u_{n}^{1}\right)-h\left(y_{\nu }^{1}|y_{n}^{\nu +1}\right)\geq h\left(u_{n}^{1}\right)-h\left(y_{\nu }^{1}\right), \end{array}$$

where (a) follows because ${\left[\Psi_{n}^{T}|\Phi_{n}^{T}\right]}^{T}$ is an orthogonal matrix. Letting ${\left(y_{G}\right)}_{\nu }^{1}$ correspond to the jointly Gaussian sequence with the same second-order moments as $y_{\nu }^{1}$, and recalling that the Gaussian distribution maximizes differential entropy for a given covariance, we obtain the upper bound

(A125) $$h\left(y_{\nu }^{1}\right)\leq h\left({\left(y_{G}\right)}_{\nu }^{1}\right)\overset{\left(a\right)}{=}\frac{1}{2}\log\left({\left(2\pi e^{}\right)}^{\nu }\det\left(\Psi_{n}\mathrm{diag}{\left\{\sigma_{u_{i}}^{2}\right\}}_{i=1}^{n}\Psi_{n}^{T}\right)\right)\overset{\left(b\right)}{\leq }\frac{\nu }{2}\log\left(2\pi e^{}\max{\left\{\sigma_{u_{i}}^{2}\right\}}_{i=1}^{n}\right),$$

where (a) follows since the ${\left\{u_{i}\right\}}_{i=1}^{n}$ are independent, and (b) stems from the fact that $\Psi_{n}\in R^{\nu \times n}$ has orthonormal rows. Since $\max{\left\{\sigma_{u_{i}}^{2}\right\}}_{i=1}^{n}$ is bounded for all *n*, we obtain by substituting (A125) into (A124) that $\lim_{n\to \infty }\frac{1}{n}\left(h\left(y_{n}^{\nu +1}\right)-h\left(u_{n}^{1}\right)\right)\geq 0$. The combination of this with (A123) yields $\lim_{n\to \infty }\frac{1}{n}\left(h\left(y_{n}^{\nu +1}\right)-h\left(u_{n}^{1}\right)\right)=0$, satisfying Condition ii) in Definition 2. From this, the proof is completed by noting that $u_{1}^{\infty }$ satisfies Condition i) in Definition 2. This completes the proof.   □

**Lemma** **A2.**
*Let A(z) be a causal, finite-order, stable and strictly minimum-phase rational transfer function with impulse response $a_{0},a_{1},\ldots$ such that $a_{0}=1$. Then, $\lim_{n\to \infty }\lambda_{1}\left(A_{n}A_{n}^{T}\right)>0$ and $\lim_{n\to \infty }\lambda_{n}\left(A_{n}A_{n}^{T}\right)<\infty$.*

**Proof** **of Lemma A2.** The fact that $\lim_{n\to \infty }\lambda_{n}\left(A_{n}A_{n}^{T}\right)$ is upper bounded follows directly from the fact that A(z) is a stable transfer function. On the other hand, $A_{n}A_{n}^{T}$ is positive definite, with $\lim_{n\to \infty }\lambda_{1}\left(A_{n}A_{n}^{T}\right)\geq 0$. Suppose that $\lim_{n\to \infty }\lambda_{1}\left(A_{n}A_{n}^{T}\right)=0$. If this were true, then it would hold that $\lim_{n\to \infty }\lambda_{n}\left(A_{n}^{-1}A_{n}^{-T}\right)=\infty$. But $A_{n}^{-1}$ is the lower triangular Toeplitz matrix associated with $A^{-1}\left(z\right)$, which is stable (since A(z) is minimum phase), implying that $\lim_{n\to \infty }\lambda_{n}\left(A_{n}^{-1}A_{1}^{-T}\right)<\infty$, thus leading to a contradiction. This completes the proof.   □

We re-state here (for completeness and convenience) the unnumbered lemma in the proof of Reference [16], Theorem 1, as follows:

**Lemma** **A3.**
*Let the transfer function G(z) satisfy Assumption 1 and suppose it has no poles. Then,*

(A126) $$\begin{array}{c} \lambda_{l}\left(G_{n}G_{n}^{T}\right)=\left\{\begin{array}{cc} \alpha_{n,l}^{2}{\left(\rho_{l}\right)}^{-2n} & ,\mathrm{if}\;l\leq m,\\ \alpha_{n,l}^{2} & ,otherwise\;, \end{array}\right. \end{array}$$

*where the elements in the sequence $\left\{\alpha_{n,l}\right\}$ are positive and increase or decrease at most polynomially with n.*

**Lemma** **A4.**
*Let A,B be matrices with the same dimensions. Then,*

(A127) $$\begin{array}{c} \lambda_{\min}\left(\left(A+B\right){\left(A+B\right)}^{T}\right)\geq \lambda_{\min}\left(AA^{T}\right)+\lambda_{\min}\left(BB^{T}\right)-2\sigma_{\max}\left(A\right)\sigma_{\max}\left(B\right). \end{array}$$

**Proof.** For every x such that ∥x∥=1,

(A128) $$\begin{array}{c} x^{T}\left(A+B\right){\left(A+B\right)}^{T}x=x^{T}AA^{T}x+x^{T}BB^{T}x+x^{T}AB^{T}x+x^{T}BA^{T}x\\ \geq \lambda_{\min}\left(AA^{T}\right)+\lambda_{\min}\left(BB^{T}\right)-2\sigma_{\max}\left(A\right)\sigma_{\max}\left(B\right), \end{array}$$

where the last inequality holds because $AA^{T}$ and $BB^{T}$ are symmetric and because of the Cauchy-Schwartz inequality. The proof is completed by noting that (A128) holds for the x that minimizes $x^{T}\left(A+B\right){\left(A+B\right)}^{T}x$, and $\lambda_{\min}\left(\left(A+B\right){\left(A+B\right)}^{T}\right)=\min_{x:∥x∥=1}x^{T}\left(A+B\right){\left(A+B\right)}^{T}x$.   □
